# Dissecting genetic architecture of grape proanthocyanidin composition through quantitative trait locus mapping

**DOI:** 10.1186/1471-2229-12-30

**Published:** 2012-02-27

**Authors:** Yung-Fen Huang, Agnès Doligez, Alexandre Fournier-Level, Loïc Le Cunff, Yves Bertrand, Aurélie Canaguier, Cécile Morel, Valérie Miralles, Frédéric Veran, Jean-Marc Souquet, Véronique Cheynier, Nancy Terrier, Patrice This

**Affiliations:** 1 UMR AGAP, INRA, 2, place Viala, 34060 Montpellier, France; 2 INRA, UMR1083 SPO, 2, place, Viala, 34060 Montpellier, France; 3 UMT Geno-Vigne®, IFV, 2, place Viala, 34060 Montpellier, France; 4 UMR Génomique Végétale, INRA UEVE ERL CNRS, 2, rue Gaston Crémieux, 91057 Evry, France; 5 Department of Ecology and Evolutionary Biology, Brown University, 80 Waterman Street, Box G-W, Providence, RI 02912, USA

## Abstract

**Background:**

Proanthocyanidins (PAs), or condensed tannins, are flavonoid polymers, widespread throughout the plant kingdom, which provide protection against herbivores while conferring organoleptic and nutritive values to plant-derived foods, such as wine. However, the genetic basis of qualitative and quantitative PA composition variation is still poorly understood. To elucidate the genetic architecture of the complex grape PA composition, we first carried out quantitative trait locus (QTL) analysis on a 191-individual pseudo-F1 progeny. Three categories of PA variables were assessed: total content, percentages of constitutive subunits and composite ratio variables. For nine functional candidate genes, among which eight co-located with QTLs, we performed association analyses using a diversity panel of 141 grapevine cultivars in order to identify causal SNPs.

**Results:**

Multiple QTL analysis revealed a total of 103 and 43 QTLs, respectively for seed and skin PA variables. Loci were mainly of additive effect while some loci were primarily of dominant effect. Results also showed a large involvement of pairwise epistatic interactions in shaping PA composition. QTLs for PA variables in skin and seeds differed in number, position, involvement of epistatic interaction and allelic effect, thus revealing different genetic determinisms for grape PA composition in seeds and skin. Association results were consistent with QTL analyses in most cases: four out of nine tested candidate genes (*VvLAR1*, *VvMYBPA2*, *VvCHI1*, *VvMYBPA1*) showed at least one significant association with PA variables, especially *VvLAR1 *revealed as of great interest for further functional investigation. Some SNP-phenotype associations were observed only in the diversity panel.

**Conclusions:**

This study presents the first QTL analysis on grape berry PA composition with a comparison between skin and seeds, together with an association study. Our results suggest a complex genetic control for PA traits and different genetic architectures for grape PA composition between berry skin and seeds. This work also uncovers novel genomic regions for further investigation in order to increase our knowledge of the genetic basis of PA composition.

## Background

**Figure 1:**
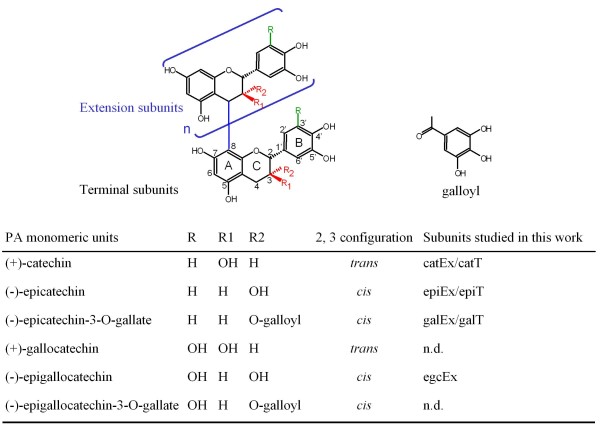
**Structures of proanthocyanidins and monomeric subunits**. A generic structure of proanthocyanidin is shown and the possible configurations are highlighted in colour. "n" indicates the number of extension units, variable according to plant species and tissues. The general chemical structure of PA monomeric subunit includes a C6-C3-C6 skeleton which is called the A-C-B rings. The carbon nomenclature is indicated as numbers next to the corresponding carbon. The B-ring generally bears two or three hydroxyl groups. According to the stereochemistry of carbons 2 and 3 on the C-ring, the PA monomeric subunit could be in 2,3-*trans *(e.g. (+)-catechin) or in 2,3-*cis *configuration (e.g.(-)-epicatechin). The structure of galloyl is shown next to PA generic structure. The right column denotes the subunits studied in this work where "Ex" indicates "extension" units and "T", terminal subunits/monomers. n.d., not detected.

Proanthocyanidins (PAs), or condensed tannins, are flavonoid polymers widespread throughout the plant kingdom. They accumulate in many organs and tissues to provide protection against pests [1]. They are also determinant in food quality and their beneficial effects on human health are increasingly investigated [1,2]. These diverse qualities are directly linked to PA chemical structures. As polymers, PA structure varies depending on the degree of polymerisation and the nature of building blocks, the flavan-3-ols (differences in stereochemistry, hydroxylation pattern on the B-ring and presence/absence of a galloyl group, Figure 1). Our understanding of PA biosynthesis has been significantly improved through the isolation of two genes coding for leucoanthocyanidin reductase (LAR, [3]) and anthocyanidin reductase (ANR, [4,5]), two specific enzymes for the formation of flavan-3-ols, respectively (+)-(gallo)catechin and (-)-epi(gallo)catechin. However, several issues concerning PA composition require further study, such as the synthesis of galloylated units, the genetic mechanism of polymerisation, and the origin of extension units, since all flavonoid intermediates are believed to assume a 2,3-*trans *configuration, similar to the 2,3-configuration of (+)-(gallo)catechin (Figure 1), while major PA extension blocks assume a 2,3-*cis *configuration (e.g. (-)-epicatechin, Figure 1). Moreover, few studies are available on the genetic basis of PA composition quantitative variation [6,7].

It is of great interest to understand PA genetics in grape since PAs are involved in grapevine self-defence mechanisms and are responsible for major organoleptic properties of red wine [8-10]. Because of its rich PA composition and the multiple genetic and genomic tools available for this species, such as the whole genome sequence [11,12], grape could represent also an interesting model for PA genetic study. Indeed, in Arabidopsis, a major model for PA studies, PAs are only detected in the seed coat with the presence of (-)-epicatechin as sole building block. By contrast, PAs are present in different organs of grapevine and are composed of four major building blocks: (+)-catechin, (-)-epicatechin, (-)-epigallocatechin and (-)-epicatechin-3-*O*-gallate [13-16] while (+)-gallocatechin and (-)-epigallocatechin-3-*O*-gallate are present in trace amounts only [15]. PAs are abundant in grape berries with drastic differences in composition between skin and seeds: total content is usually higher in seeds while polymer size is much larger in skin [15,16]. In terms of constitutive building blocks, (-)-epigallocatechin is a major component of grape skin PAs [15] while it is not detectable in seeds [16]; (-)-epicatechin-3-*O*-gallate is present in large proportion as both extension and terminal subunits in seeds while it is present only in small amounts in skin [15,16]. Advances in understanding grape PA synthesis have been mainly obtained through homologous cloning [14,17-21]. However, the complex PA composition within a tissue and the contrasted composition between tissues suggest a complex interaction of many factors in the determinism of grape PA composition.

One way to assess genetic determinism of trait variation without *a priori *knowledge is quantitative trait loci (QTL) mapping. QTL mapping makes use of segregating populations and gives global insights into the genetic architecture of the target phenotype, *i.e*. the number, position and effects of genomic regions [22]. Among all available mapping methods, the multiple-QTL approach is particularly suitable for complex trait analysis since it uses simultaneously multiple marker intervals with possible inclusion of epistasis terms in QTL mapping model [23]. Instead of creating segregating populations, one can also explore the existing diversity through association mapping to identify loci involved in phenotypic variation [24]. According to the genetic architecture of target traits, one can build appropriate breeding strategy and/or develop further gene function studies.

The aim of this work was to investigate the genetic determinism of PA composition variation in both skin and seeds of grape. For this purpose, we first characterised skin and seed grape PA composition in a pseudo-F1 progeny derived from a cross between Syrah and Grenache cultivars. Three categories of PA variables were constructed in order to capture the complex PA profiles: total content variables, percentages of constitutive subunits, which assessed the biosynthesis efficiency among building blocks, and composite ratio variables, which included estimation of polymer size and metabolite flux between building blocks. We then applied a multiple-QTL genome scan to identify main effect QTLs and pairwise epistatic interactions for PA variables. Nine functional candidate genes, among which eight co-located with QTLs, were sequenced and their SNP-phenotype associations were investigated in a grapevine diversity panel. We present here the first extensive study of genetic architecture of PA composition in grape and confirm the involvement of some candidate genes in PA composition variation.

## Methods

### Plant material

The two grapevine populations used in this study have been previously described [25-27]. Briefly, the QTL mapping population (S × G) consisted of a pseudo-F1 progeny of 191 individuals from a cross between two wine grape cultivars, Syrah (S) and Grenache (G) and was maintained under classical local training system (3300 plants/ha plant density) at Montpellier SupAgro Domaine du Chapitre (Hérault, France). The S × G population was planted in two blocks. Each individual from the progeny was planted in two elementary plots (one per block) comprising five plants each. Parental cultivars were also planted in each block with nine and 43 elementary plants for Syrah and Grenache, respectively. The association mapping population (CC) consisted of a core-collection of 141 cultivars maximising agro-morphological diversity for 50 quantitative and qualitative traits, and maintained at INRA Domaine de Vassal (Hérault, France) [26].

### DNA extraction, marker genotyping and gene sequencing

DNA extraction and marker genotyping were already described in Adam-Blondon et al. [28]. In order to densify a previous 97-SSR linkage map of the S × G population [25], additional SSR markers, heterozygous both in Syrah and Grenache, were chosen from recent grapevine reference maps [29]. Based on the 12X grapevine reference sequence (Grape Genome Browser http://www.genoscope.cns.fr), we designed primers for candidate gene amplification using Primer3 (http://www.bioinformatics.nl/cgi-bin/primer3plus/primer3plus.cgi) with default parameters. Primers used in this study are listed in Additional file 1. For SNP analysis, gene fragments were amplified, sequenced and analysed as described in [30].

### Linkage map construction

Framework maps were constructed based on the 97-marker linkage map of [25] with 56 additional SSRs. All 153 markers had a genotypic error rate lower than 1.5%, using Tmap as check [31]. Linkage maps were constructed using CarthaGène 0.999R [32] as described in [33] with Haldane mapping function. The "Syrah" and "Grenache" framework maps were composed of 121 SSRs (total length 1118.8 cM), and 133 SSRs (1349.4 cM total length) respectively. The "Consensus" framework map spanned 1256.4 cM based on 153 SSRs among which over 70% allowed segregation in four genotypic classes in the pseudo F1 progeny (ab × ac and ab × cd). Marker order reliability was ensured at LOD 2 threshold. Segregation distortion on genotypic classes was verified by a *χ*^2 ^test according to the segregating type of each marker for the different maps (e.g. for markers segregating as ab × cd in the consensus map, the H_0 _hypothesis was ac:bc:ad:bd = 1:1:1:1). Twenty-five markers out of 153 exhibited distorted segregation (*P *< 0.05) and were mainly grouped on chromosomes 3 (4 markers), 4 (6 markers) and 10 (5 markers). Markers on chromosome 4 exhibited the most significant allelic deviation (*P *< 0.001) due to segregating distortion between Syrah alleles (aa:ab ~2/3:1/3).

### Phenotyping and PA variable construction

Grapes were harvested at maturity (20° Brix). For each genotype, eight representative berry clusters were harvested from the five plants of the elementary plot. Sample homogenisation was based on the accumulation of total solutes (principally sucrose), a major marker of berry development during ripening. Berry density was assessed by floatation in salt solutions [34]. Twenty-five berries with a density between 130 and 160 g NaCl/L were randomly selected. In the present study, we focused on the analysis of berry skin and seeds since PA concentration is quite low in flesh, flesh PAs accounting for only 2-6% of the total berry PA content [35]. Berry skin and seeds were separated, ground in liquid nitrogen and stored at-80°C until analysis. PAs were extracted and analysed by high performance liquid chromatography (HPLC) after acid-catalysed cleavage in the presence of phloroglucinol according to [36]. For the S × G population, both skin and seeds were analysed for 2 consecutive years: skin was analysed in 2005 (1 block) and 2006 (2 blocks) while seeds were analysed in 2006 (2 blocks) and 2007 (2 blocks). For the CC population, skin was analysed in 2005 and 2006 and seeds in 2006.

In order to obtain an exhaustive view of PA composition, three categories of PA variables were studied in this work: total PA content, subunit percentage and composite ratio variables. For total content variables, concP (mg/g fresh weight) reflects the biosynthesis intensity in each tissue, concB (mg/berry) brings total content to single berry level by taking into account berry size while concK (mg/kg berries) is a common enological measurement taking into account yield-related traits. Since all PA building blocks are derived from the same intermediate structure, naringenin chalcone, we used the percentage of each PA subunit to total subunit quantity to assess partitioning efficiency between PA building blocks. Our PA characterisation method did not distinguish between terminal units of polymers and flavan-3-ol monomers. The notation ending with "T" corresponds thus to the sum of terminal subunits and monomers. We also constructed composite ratio variables, including mean degree of polymerisation (mDP), ratio of 2,3-*trans*- to 2,3-*cis*-subunits in extension position (Ftranscis_Ex), terminal position (Ftranscis_T) and overall subunits (Ftranscis_all) and the ratio of B-ring di-hydroxylated to B-ring tri-hydroxylated subunits (F3pr35). Variables studied in this work are summarised in Table 1.

**Table 1:** PA variables used in this study and their description

PA traits	Skin/Seed^a^	Definition	Biological/biochemical significance
Total content			
concP	+/+	mg/g fresh tissue	Biosynthesis intensity per gram of tissue
concB	+/+	mg/berry	Taking berry size into account
concK	+/+	mg/Kg berries	Taking yield related-trait into account
Subunit percentage		100·(subunit content)/(total content)^b^	Assessment of partitioning efficiency of
catEx	+/+	(+)-catechin Extension subunit	PA biosynthesis among different subunits
epiEx	+/+	(-)-epicatechin Extension subunit	
galEx	+/+	(-)-epicatechin-3-O-gallate Extension subunit	
egcEx	+/-	(-)-epigallocatechin Extension subunit	
catT	+/+	(+)-catechin Terminal subunit/monomer	
epiT	+/+	(-)-epicatechin Terminal subunit/monomer	
galT	+/-	(-)-epicatechin-3-O-gallate Terminal subunit/monomer	
Composite variables			
mDP	+/+	mean Degree of Polymerisation(Total number of extension and terminal/monomer subunits)/(Number of terminal subunit/monomer)	Assessment of PA polymer size
F3pr35^c^	+/-	(catEx + epiEx + galEx + catT + epiT)/(egcEx)	Assessment of flux between B-ring di-OH and tri-OH subunits
Ftranscis_Ex^c^	+/+	Skin:catEx/(epiEx + galEx + egcEx) Seed: catEx/(epiEx + galEx)	Assessment of flux between 2,3-trans subunit and 2,3-cis subunit in extension part
Ftranscis_T^c^	+/+	Skin: catT/epiT Seed: catT/(epiT + galT)	Assessment of flux between 2,3-trans subunit and 2,3-cis subunit in terminal part
Ftrancis_all^c^	+/+	Skin: (catEx + catT)/(epiEx + galEx + egcEx + epiT) Seed: (catEx + catT)/(epiEx + galEx + epiT + galT)	Global assessment of flux between 2,3-trans subunit and 2,3-cis subunit(extension + terminal/monomer)

^a ^Presence/Absence (indicated by +/-) of a given trait in grape berry tissues.

^b ^based on PA content expressed in mg/g fresh tissue

^c ^F for Flux.

### Phenotypic data analysis

All statistical analyses were performed with R software [37]. We identified the best-fit mixed model for each PA variable through Bayesian information criterion (BIC) in order to extract the best linear unbiased predictors (BLUPs) for genotypic values and to estimate the broad sense heritability (*H^2^*). Mixed model fit was performed with lme4 package [38]. The mixed model assumption of normality of residual and BLUPs was checked after model fitting by quantile-quantile plot comparing the distribution of residual and random effect predictors to a theoretical normal distribution (Additional file 2). No data transformation was performed for PA variables measured in the two populations. More information regarding phenotypic data analysis and best-fit model for each PA variable is in Additional file 3.

### QTL analysis

QTL analysis was performed on the genotypic BLUPs with R/qtl package [39]. Multiple QTL regression was carried out with "stepwiseqtl" function. This approach uses forward/backward selection to identify a multiple-QTL model with inclusion of both main effect QTLs and pairwise interactions. Maximum QTL number was set to 10 for forward selection (max.qtl = 10). Model choice was made via a penalized LOD score (pLOD) which is the LOD score for the model (the log likelihood ratio comparing the full model to the null model without QTL) with penalties on the number of QTLs and pairwise QTL × QTL interactions [40]. For each PA variable, specific penalties for main effect and digenic pairwise interaction terms were derived from 1000 permutations of two-dimensional scan (the "scantwo" function, method = "hk", n.perm = 1000) and penalties at genome-wide error rate of 0.05 were used for multiple-QTL model fitting. The QTL model with the largest pLOD was identified as the most probable one. Once determined the multiple QTL model, we refined QTL position ("refineqtl" function) and estimate *R^2 ^*for the whole model and each term of the model, the individual LOD score of each term and the genotypic effect ("fitqtl" function). The "lodint" function was used to derive LOD-1 QTL location confidence interval. Allelic effects for consensus QTLs were estimated as described by Segura *et al*. [41]. Genome scan was performed with a 1 cM step.

### Association analysis

Nine candidate genes were selected for association test according to their function and co-localisation with QTLs. Prior to association test, we used R kinship package [42] to perform model comparison among different nested models according to [43] in order to select the best fitted model for association test for each PA variable. Ancestry structure and kinship matrix were estimated based on 20 SSR markers located throughout the whole genome as described in [25].

After model comparisons, we used TASSEL package to perform association tests [44]. Two models were used: one accounting for ancestry structure effect (with General Linear Model, or GLM in TASSEL) the other for both ancestry structure effect and random genetic background effect (with Mixed Linear Model, or MLM in TASSEL). Association tests were performed on BLUPs for skin variables and raw data for seed variables since seed data were available for 2006 only. For GLM analyses, tests were run with 1000 permutations allowing the determination of site-wise *P *value, whi ch is the probability of a greater *F *value under the null hypothesis that polymorphism was independent of the phenotype. The adjusted *P *value (called p_adj_Marker in TASSEL), is the site-wise *P *value adjusted for multiple tests which takes into account the dependence between SNPs due to linkage disequilibrium. Because each gene was tested independently, we used an additional Bonferroni correction to correct for the number of studied genes (nine) which led to a threshold of 0.0056 for the adjusted *P *value. As the permutation method is not available for MLM, we used the threshold proposed by Benjamini and Hochberg [45] with *q *equal to 0.05 which led to a threshold of 0.0039. The effect of minor genotypic frequency and non-normality of observed trait distribution was checked (details in Additional file 4).

## Results

### Phenotype analysis

#### PA variable distribution and heritability

**Figure 2:**
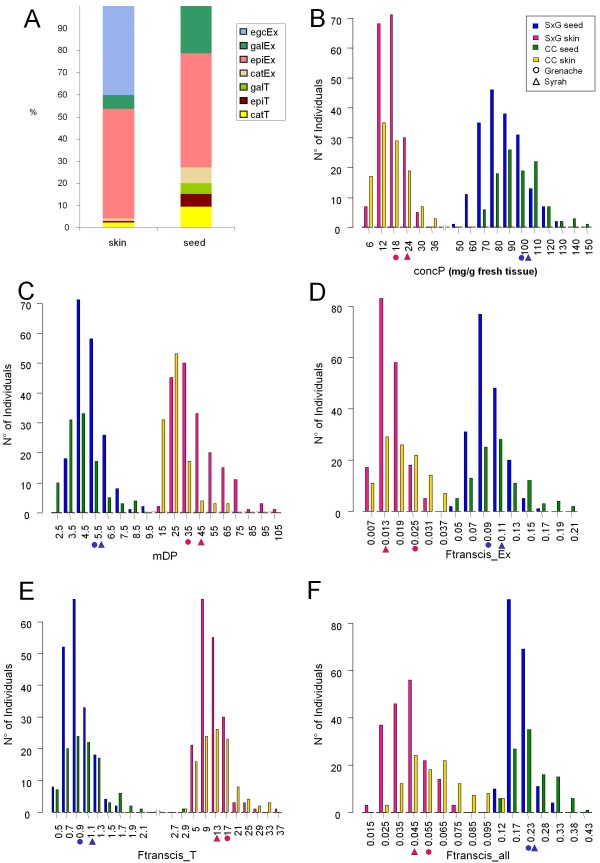
**Comparative composition of skin and seed PA in 2006 (A) and distribution of PA variables of S × G and CC populations in 2006 for concP (B), mDP (C), Ftranscis_Ex (D) Ftranscis_T (E) and Ftranscis_all (F)**. (**A**) PA composition in skin and seeds based on the S × G offspring average is shown. Each building block is presented as the offspring average percentage of total content. (**B**-**F**) Distribution of PA variables in S × G and CC populations in 2006. Upper limits of data interval are indicated under the x-axis. Full symbols near x-axis show mean parental values for S × G population, circle for Grenache and triangle for Syrah, in pink for skin values and in blue for seed value as for the offspring histograms.

For the S × G population, all PA variables showed continuous distribution and transgressive segregation and variation extent was equivalent in the S × G and CC populations (Figure 2, Additional file 5). In agreement with previous studies [15,16,46], samples taken in 2006, for which both berry skin and seeds were analysed, displayed different PA composition between tissues as illustrated in Figure 2A with the mean values in the S × G population. For both S × G and CC populations, (-)-epicatechin (epiEx) was the predominant extension subunit in all tissues while (-)-epigallocatechin (egcEx) was only detected in skin. (+)-catechin (catT) was the predominant terminal subunit/monomer in both skin and seed while galloylated units (galEx and galT) were more abundant in seed PA. Each subunit exhibited large variation according to genotype. For instance in skin, (-)-epigallocatechin (instead of (-)-epicatechin) could be the predominant subunit in the extension position (67.9%) (Additional file 5).

PA content variables (concP, concB, concK) reached higher values in seeds than in skin regardless of the unit, as illustrated for concP (Figure 2B), which exhibited the largest difference between tissues. Comparison of composite PA ratio variables showed different range of variation between skin and seed. PAs were on average 8-times shorter in seeds than in skin with wide variation in skin (Figure 2C). All three ratio variables assessing the flux between 2,3-*trans *and 2,3-*cis *forms (Ftrancis series) pointed to different kinetics for extension and terminal positions: *trans *subunits were more abundant in skin for terminal units/monomers (Ftranscis_T) while they were much reduced in seeds when considering extension positions alone (Ftranscis_Ex) or extension plus terminal subunits/monomers (Ftranscis_all, Figure 2D-F). Since the major extension blocks were in *cis*-configuration, i.e. (-)-epi(gallo)catechin, Ftranscis_Ex was always less than 1 both in skin and seeds. The higher Ftranscis_T in skin conformed to the fact that (+)-catechin was the predominant terminal subunits/monomer in skin. (Figure 2B). Means of each PA variable measured in both skin and seeds were systematically different (paired *t*-test, *P *< 0.001, data not shown).

For S × G population, average *H^2 ^*of PA variables was 0.56 (from 0.24 to 0.82) and 0.44 (from 0.26 to 0.54) in skin and seeds, respectively. No significant difference in *H^2 ^*magnitude was detected between skin and seeds (*t*-test, *P *= 0.053). Nevertheless, higher *H^2 ^*were observed for skin variables, especially catT and mDP (0.76 and 0.82, respectively, Additional file 5). A high *H^2 ^*value was also found for these two traits in CC (0.86 and 0.72 for catT and mDP, respectively, Additional file 5).

#### PA variable correlation

**Figure 3:**
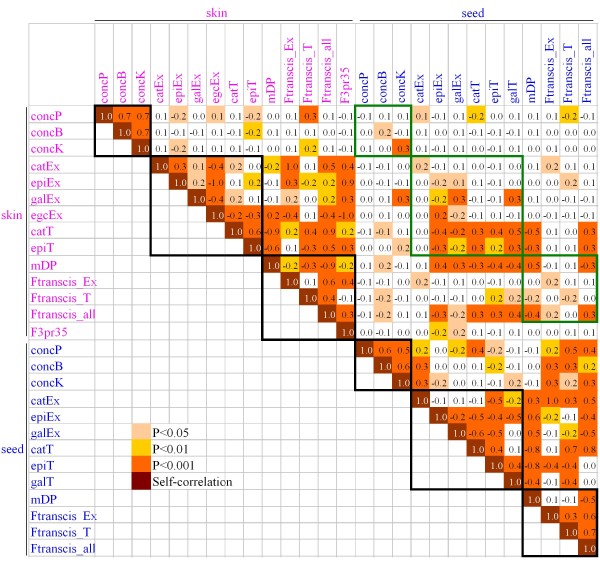
**PA variable correlation based on genotypic BLUP of S × G population**. The Pearson pairwise correlation coefficient (*ρ*) is shown and colour codes give the significance of correlation tests. Skin variables are indicated in pink and seed variables are indicated in blue. The bold black lines delimit the pairwise correlation inside a tissue for a given variable category, *i.e*. total content, subunit percentage and composite variables. The bold green lines delimit the pairwise correlation between tissues for a given variable category.

We performed PA variable correlation on genotypic values (BLUPs) from S × G population because the two-year data available both in skin and seeds allowed us to work with a much reduced environmental effect. All three total content variables were highly correlated within a given tissue while significant correlations between tissues were only observed for concB and concK (Figure 3). Among subunit percentage variables in skin (Figure 3), the most noticeable features were the significant negative correlation between egcEx (B-ring tri-hydroxylated subunit) and all other units (B-ring di-hydoxylated subunits) and the positive correlation between (-)-epicatechin and (+)-catechin either in extension position (Ex) or in terminal/monomer position (T). In seeds, the most noticeable feature was the significant correlation of epiT with all other variables (negative with extension units and positive with terminal units) while epiEx was negatively correlated with other subunits. For the same subunit in a given tissue, no highly significant correlation was observed between extension and terminal position, except the negative correlation between epiEx and epiT in seeds (*P *< 0.001). Significant correlation between subunit percentage variable and composite variables inside a tissue reflected the variable construction (Table 1). Between tissues, significant positive correlations were observed for concK, most of terminal subunits/monomers pairs, galEx and also mDP and Ftranscis_all.

### QTL analysis

#### Global features of PA QTLs

**Figure 4:**
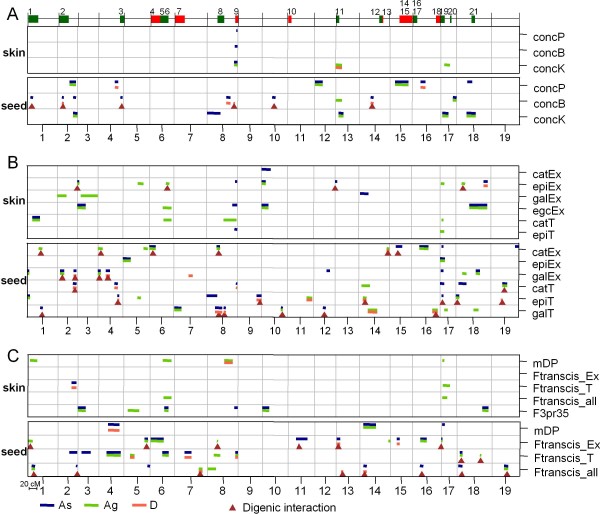
**Overview of skin and seed PA QTLs identified on the consensus map for total content (A), subunit percentage (B) and composite variables (C)**. For each variable category, two panels are shown: the upper one for QTLs in skin and the lower one for seeds. The x-axis of each panel spans the whole genome where chromosome sizes are proportional to genetic distance of consensus map and the chromosome numbers are indicated under the x-axis of lower panels. QTLs are indicated by horizontal lines with width corresponding to LOD-1 confidence interval. As, Ag and D respectively indicate additive effect from Syrah alleles, additive effect from Grenache alleles and dominance effect which were estimated according to [41]. Color codes correspond to major effects for each QTL, estimated as (|As| or |Ag| or|D|)/(|As|+|Ag|+|D|) > 0.30. Triangles indicate loci involved in digenic pairwise interactions. Grape candidate genes for PA synthesis are indicated on the upper black line of (A) where bar size is proportional to the flanking marker interval of the gene. Green bars are for genes coding for synthetic enzymes while red bars are for genes coding for transcription factors. The number above the flanking marker interval indicates the corresponding candidate gene: 1, *VvLAR1 *(leucoanthocyanidin reductase) [14]; 2, *VvLDOX *(leucoanthocyanidin dioxygenase) [17]; 3, *VvF3H *(flavanone 3-hydroxylase) [17]; 4, *VvMYB5b *[47]; 5, *VvC4H *(cinnamate 4-hydroxylase); 6, *VvF3'5'H*s (flavonoid 3'-5' hydroxylases) [18-20]; 7, *VvMYC1 *[48], 8, *VvPAL *(phenylalanine ammonia-lyse) [17]; 9, *VvMYB5a *[49]; 10, *VvMYBPA2 *[50]; 11, *VvCHIs *(chalcone isomerases) [17,21]; 12, *VvCHS *(chalcone synthase) [17]; 13, *VvWDR2 *[51]; 14, *VvMYBPA1 *[52]; 15, *VvMYCA1 *[51]; 16, *VvPAL *(phenylalanine ammonia-lyse) [17]; 17, *Vv4CL *(4-coumaroyl CoA ligase); 18, *VvWDR1 *[51]; 19, *VvLAR2 *(leucoanthocyanidin reductase) [14]; 20, *VvF3'H*s (flavonoid 3'-hydroxylases) [18-20], 21, *VvDFR *(dihydroflavonol reductase) [17]. Detailed genetic maps with marker names are available in Additional file 7.

**Figure 5:**
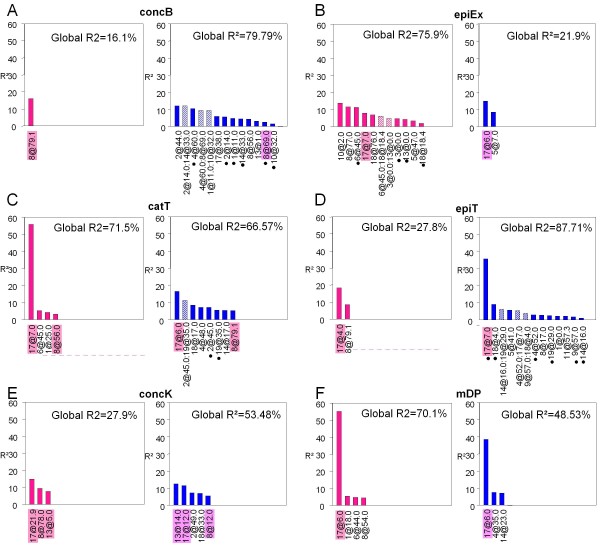
***R^2 ^*distribution of skin and seed PA QTLs identified on the consensus map for concB (A), epiEx (B), catT (C), epiT (D), concK (E) and mDP (F)**. *R^2 ^*of main effect QTLs (solid bar) and *R^2 ^*of digenic epistatic interaction (hatched bars) are sorted according to their magnitude. Skin variables are indicated in darkpink and seed variables in blue. Locus names are indicated on the x-axis and should be read as chromosome@position_on_the_chromosome. Locus names are highlighted in pink for loci identified in both skin and seed for the same variable; we considered loci as "common" loci when their LOD-1 confidence interval overlapped. Loci involved in digenic epistasis are indicated by a dark dot under the locus names for which *R^2 ^*was estimated without inclusion of the associated interaction.

We performed QTL detection with genotypic BLUPs both on consensus and parental maps. In total, 103 *vs *43 QTLs and 24 *vs *2 digenic epistatic interactions were identified on the consensus map for seed and skin PA variables, respectively (Figure 4). QTLs detected on parental maps were generally also detected on the consensus map except for skin concP, concK, catEx, galEx, epiT, F3pr35 and seed concP where additional QTLs were identified through parental detection (Additional file 6). More QTLs and digenic pairwise interactions were identified on the consensus map than on parental maps, allowing some QTL models to explain more than 80% of the BLUP variance in consensus mapping (Additional file 6), as illustrated in the case of epiT in seeds (Figure 5D). Some loci were involved in phenotypic variation almost exclusively through digenic epistasis such as locus 10@32 for seed concB or locus 14@16.0 for seed epiT (Figure 5). Loci were mainly of additive effect while dominance was predominant at some loci for concK, epiEx, mDP and Ftranscis_T in skin and galEx, epiT, Ftranscis_T and Ftranscis_all in seeds (Figure 4). Among all detected QTLs, only 10 main effect loci overlapped for the same variable in both tissues: 1 for concB, 2 for concK, 1 for epiEx, 1 for galEx, 2 for catT, 1 for epiT, 1 for mDP and 1 for Ftranscis_T (see Figure 5 for some examples). Parental alleles contribution to these common loci was not always consistent across tissues (Additional file 6), which could be an indication of tissue-specific genetic mechanisms. Different genetic architectures were observed for the same PA variable between berry skin and seeds as illustrated in Figure 5: few moderate QTLs (< 3) or no QTL in skin *vs *several (> 5) small to moderate QTLs with possible involvement of epistasis in seeds (for concP, concB, catEx, galEx, Ftranscis_Ex and Ftranscis_T, illustrated by concB in Figure 5A); many QTLs with involvement of epistasis in skin *vs *a small number (2) of main effect QTLs in seeds (epiEx in Figure 5B); a major QTL (*R^2 ^*> 50%) and some QTLs of moderate effect in skin *v.s*. many QTLs of small to moderate effect in seeds (for catT and Ftranscis_all, illustrated by catT in Figure 5C), or few moderate QTLs in skin *vs *many moderate QTLs in the presence of a QTL of large effect and epistasis in seeds (epiT, Figure 5D). Conversely, similar genetic architecture between skin and seeds was observed for concK (only moderate main effect QTLs, Figure 5E) and mDP (a major QTL and a few QTLs of moderate effect, Figure 5F). Details regarding position, major allelic effect, LOD score, LOD-1 confidence interval and percentage of explained variation (*R^2^*) for each QTL are given in Additional file 6.

#### PA total content

In skin, 1, 1 and 3 QTLs were identified on the consensus map for concP, concB and concK, respectively. One additional QTL for concP was identified through parental detection on the Syrah map. Conversely, for concK, all QTLs exhibited a major Grenache allelic effect and one additional QTL was identified on chromosome 9 on the Grenache map.

In seeds, 6, 10 and 5 QTLs were identified for concP, concB, and concK, respectively. The locus positioned at 40-50 cM on chromosome 2 was identified for all three variables. Epistasis was strongly involved in genetic architecture of concB and in total accounted for around 30% of the BLUP variance (Figure 1).

In summary, fewer total content QTLs were detected in skin than in seeds. Common loci between skin and seeds for concB and concK were identified on chromosomes 8, 13, and 17. For each tissue, one locus was identified to be common to the three total content variables: the QTL on chromosome 8 for skin and the QTL on chromosome 2 for seeds.

#### Simple variables: percentage of constitutive units

In skin, 1, 9, 3 and 5 QTLs respectively were identified on consensus map for catEx, epiEx, galEx, and egcEx, with several overlapping QTLs (Figure 4). For catEx, two additional QTLs on chromosomes 14 and 18, were specifically identified on the Grenache map. Pairwise interactions were identified for epiEx. For terminal subunits/monomers, 2 and 4 consensus QTLs were detected for epiT and catT, respectively. These two traits had co-locating loci on chromosomes 8 and 17 with an especially large *R^2 ^*for the locus on chromosome 17 (55.8% for catT).

In seeds, 2 QTLs for epiEx were identified on the consensus map while two additional loci on chromosomes 8 and 12 were identified solely on Grenache and Syrah maps, respectively (Figure 4, Additional file 6). For all other simple variables, at least 7 QTLs and 1 pairwise interaction were involved in multiple-QTL models on the consensus map. For galEx, additional loci were identified on chromosomes 3, 5 and 9 through parental detection.

Joint consideration of the results obtained from both tissues showed that different QTLs were identified for subunits of the same nature but with different positions in the polymer (e.g. epiEx and epiT). The locus positioned at approx. 7 cM on chromosome 17 was identified for all PA simple variables in both tissues except for egcEx, galEx and catEx in skin.

#### Composite ratio variables

In skin, the best QTL model for mDP, Ftranscis_T and Ftranscis_all included only a few main effect QTLs (2 to 4 QTL, Additional file 6) without digenic interaction. The major locus on chromosome 17 was also identified for these 3 variables: in the case of mDP, it explained more than 50% of total BLUP variance (Figure 5F). Six QTLs were detected for F3pr35 on the consensus map while one additional Syrah-specific QTL was identified through parental detections on chromosome 13. For Ftranscis_Ex, QTLs were solely identified through parental detections (1 QTL for Syrah map and 2 QTLs for Grenache map).

In seeds, a three-additive-QTL model was identified for mDP while models with 10 QTLs and one to four digenic interactions were the best ones for Ftranscis_Ex, Ftranscis_T and Ftranscis_all. In addition, digenic interaction accounted for about 30% of BLUP variance for Ftranscis_Ex and Ftranscis_all (Figure 5 and Additional file 6).

In summary, different QTLs were identified for the same variables, depending on berry tissues. Among all composite variables, only the large effect QTL for mDP and Ftranscis_T on chromosome 17 was common to skin and seeds. Comparison of multiple-QTL models between both tissues showed that more digenic interactions were involved in seed variables than in skin variables.

### Association analyses on candidate genes

We positioned 21 known grape PA functional candidate genes on the genetic map using their relative position to SSR markers on the grape genome ([11], http://www.genoscope.cns.fr, Figure 4, see Additional file 7 for the names of flanking markers of candidate genes). Association tests were performed for nine functional candidate genes, eight of them co-locating with QTLs. Among candidates, there were both genes encoding flavonoid pathway enzymes and putative regulators. Genes were partially to totally sequenced (gene coverage from 25 to 100%), mainly in exons (Table 2). Two models were used for association studies since model comparison showed an equivalent fit (Additional file 8): one accounted for fixed ancestry structure effect (GLM in TASSEL), the other for both fixed ancestry structure effect and random genetic background effect (MLM in TASSEL). four out of nine genes showed at least one significant association with PA variables with consistent results between GLM and MLM (Table 3). Seventy-eight percent of significant tests (21 out of 27 tests) were common to GLM and MLM while 6 additional associations were only significant with MLM model. The reason for this discrepancy is probably that the adjusted *P*-value in GLM was estimated by taking into account dependence between tests due to linkage disequilibrium [44] while in MLM, each SNP is tested under an hypothesis of independence. Association results were consistent with QTL analyses for following gene-phenotype pairs: *VvLAR1*-skin catT and *VvLAR1*-skin mDP (Table 3). In particular, several SNPs in linkage disequilibrium for *VvLAR1 *(data not shown) were significantly associated to catT and mDP in skin while the confidence interval of the QTLs for these two variables overlapped. Conversely, we observed some SNP-phenotype associations only in the diversity panel: *VvLAR1*-skin Ftranscis_all, *VvMYBPA2*-skin concP, *VvMYBPA2*-skin concK, *VvMYBPA2*-skin mDP, *VvMYBPA2*-seed galT, *VvCHI1*-skin concP, *VvMYBPA1*-skin epiT and *VvMYBPA1*-seed Ftranscis_T.

**Table 2:** Summary of candidate genes for association tests

			Sequence (size and localisation)					Number of SNPs					
**chr**	**Gene**	**References**	**5'-UTR**	**Exon**	**Intron**	**3'-UTR**	**seq/gene size**	**5'-UTR**	**Exon**	**Intron**	**3'-UTR**	**total**	**QTL**

1	*VvLAR1*	[14]	-	1008	412	65	1420/2980	-	21	9	-	30	**Skin**: catT, mDP. **Seed**: concB, galEx, catT, epiT, Ftranscis_Ex, Ftranscis_all

6	*VvF3'5'H 1.1*	[19,20]	-	1296	50	-	1346/2325	-	4	-	-	4	**Skin**: epiEx, egcEx, catT, mDP, F3pr35, Ftranscis_all

6	*VvF3'5'H 2.1*	[19,20]	-	630	12	24	642/1932	-	3	0	0	3	**Skin**: epiEx, egcEx, catT, mDP, F3pr35, Ftranscis_all

8	*VvMYB5a*	[49]	66	687	382	36	1069/1069	1	12	2	1	16	**Skin**: concP, concB, concK, epiEx, egcEx, epiT, F3pr35. **Seed**: concB, catEx, catT, Ftranscis_T

11	*VvMYBPA2*	[50]	93	855	263	37	1148/1479	-	14	5	-	19	No QTL

13	*VvCHI1*	[17,21]	-	440	216	54	656/1486	-	3	5	1	9	**Skin**: concK, epiEx. **Seed**: concK, catT, Ftranscis_Ex, Ftranscis_all

13	*VvCHI2*	[17,21]	-	206	452	46	658/2524	-	1	6	-	7	**Skin**: concK, epiEx. **Seed**: concK, catT, Ftranscis_Ex, Ftranscis_all

15	*VvMYBPA1*	[52]	384	861	87	1 0	948/948	8	11	-	-	19	**Seed**: concP, catEx, Ftranscis_Ex

18	*VvDFR*	[17]	-	425	194	111	619/2469	-	1	-	2	3	**Skin**: egcEx. **Seed**: concP, concK, epiT

Chr, chromosome; seq/gene size, total length of sequenced exons and introns/predicted gene size.

**Table 3:** Results of the association study: significant SNP-phenotype associations along with the co-located QTL

Chr	Gene	Marker	Position	Syn/Ns	Tissue	Trait	n.obs	p.MLM	p.adj.GLM	QTL
1	*VvLAR1*	int2687	intron 4		skin	catT	112	**2.92E-04**	**9.99E-04**	Yes
		e5-2734	exon 5	Syn	skin	catT	112	**1.22E-05**	**9.99E-04**	Yes
		e1-82	exon 1	Ns (Ala ↔ Thr)	skin	mDP	115	7.08E-04	0.025	Yes
		e1-132	exon 1	Syn	skin	mDP	113	**5.44E-04**	**9.99E-04**	Yes
		e1-138	exon 1	Syn	skin	mDP	105	0.0013	0.0021	Yes
		e1-156	exon 1	Ns (Asn ↔ Lys)	skin	mDP	111	**5.19E-04**	**9.99E-04**	Yes
		e3-665	exon 3	Syn	skin	mDP	117	**3.41E-04**	**9.99E-04**	Yes
		e3-734	exon 3	Syn	skin	mDP	110	**5.66E-04**	**9.99E-04**	Yes
		int2405	intron 3		skin	mDP	94	**1.85E-04**	**9.99E-04**	Yes
		e4-2524	exon 4	Syn	skin	mDP	103	**5.58E-04**	**9.99E-04**	Yes
		int2636	intron 4		skin	mDP	104	**6.49E-04**	**9.99E-04**	Yes
		e5-2722	exon 5	Syn	skin	mDP	107	**4.82E-04**	**9.99E-04**	Yes
		e5-2776	exon 5	Syn	skin	mDP	107	**4.82E-04**	**9.99E-04**	Yes
		e5-2779	exon 5	Syn	skin	mDP	107	**5.61E-04**	**9.99E-04**	Yes
		e5-2785	exon 5	Syn	skin	mDP	107	**5.61E-04**	**9.99E-04**	Yes
		e5-2872	exon 5	Ns (Ile ↔ Met)	skin	mDP	104	**6.37E-04**	**9.99E-04**	Yes
		e5-2896	exon 5	Syn	skin	mDP	104	**6.37E-04**	**9.99E-04**	Yes
		e5-2902	exon 5	Syn	skin	mDP	104	**6.37E-04**	**9.99E-04**	Yes
		e1-156	exon 1	Ns (Asn ↔ Lys)	skin	Ftranscis_all	109	0.0032	0.0509	No
		int2687	intron 4		skin	Ftranscis_all	114	0.0025	0.042	No
		e5-2734	exon 5	Syn	skin	Ftranscis_all	114	**1.84E-04**	**9.99E-04**	No
11	*VvMYBPA2*	intron06Y	intron		skin	concP	117	**9.77E-04**	**9.99E-04**	No
		p19_GA	promoter		skin	concK	55	**4.37E-04**	**9.99E-04**	No
		p18	promoter		skin	mDP	54	**1.76E-04**	**9.99E-04**	No
		p19_GA	promoter		skin	mDP	55	**3.36E-06**	**9.99E-04**	No
		intron05M	intron		seed	galT	82	0.0015	0.3377	No
		1293 W	exon 3	Syn	seed	galT	93	0.0014	0.3067	No
		1322 W	exon 3	Ns (Leu ↔ His)	seed	galT	92	0.0013	0.3387	No
		1398Y	exon 3	Syn	seed	galT	92	0.0029	0.6773	No
		1473Y	exon 3	Syn	seed	galT	93	0.0026	0.5135	No
13	*VvCHI1*	Y183	exon 4	Syn	skin	concP	108	0.0025	0.049	No
15	*VvMYBPA1*	p277R	promoter		skin	epiT	125	0.0018	0.03	No
		702W	exon 2	Ns (Ser ↔ Thr)	seed	Ftranscis_T	68	0.0036	0.6783	No

p.MLM, *p*-value from mixed model, *p*.adj.GLM, adjusted *p*-value from GLM. Bold cases indicate significant associations in both MLM and GLM results. QTL, the candidate genes were under QTLs of the same PA variables as those associated with SNP.

## Discussion

### PA variation extent compared to previous studies

A first characterisation of PA composition in a grapevine pseudo-F1 population was provided by Hernandez-Jimenez and co-workers [46]. Their population was composed of 42 offsprings, derived from a cross between Syrah and Monastrell. In all tissues, the subunit percentage in extension position and Ftranscis-series variables of the Syrah × Monastrell population were of a magnitude and extent equivalent to those of the present study. More divergent results were observed for 1) epiT, which is more abundant in the Syrah × Monastrell population; 2) mDP, which is higher in our study and 3) total content variables, for which the population mean and variation extent was three-to two-fold larger in the present study than in [46]. Syrah, the common parent, behaved similarly in both studies although we observed 10-fold and two-fold higher total PA contents in our study for skin and seeds, respectively. The difference observed in offsprings may result from the fact that the two populations differed by one parent but environmental differences as well as PA extraction and quantification methods might also have affected PA variables, as suggested by the different PA composition of the same parental cultivar. Indeed, mixed model fit suggested that year had a major effect on PA-related variables for both tissues, which was consistent with a previous study where PA content and composition were measured in two cultivars for two consecutive years [53]. The large quantitative variation in PA variables in S × G, of equivalent extent in the CC diversity panel, underlines the interest to implement a quantitative genetic approach on a F1 population for grape PA studies.

Two studies only have characterised PA composition in different grape cultivars [54,55] with at most a 37-cultivar sample [54]. Different biochemical analyses did not allow for result comparison between this latter study and the present work. Nevertheless, CC in our work was composed of 141 grapevine cultivars of broad geographical origin (from East to West Europe) and was initially defined to maximise the diversity of 50 agro-morphological traits [26]. The PA composition variation in the diversity panel provides thus the potential to refine PA QTLs in a population of larger genetic background.

### Multiple QTL mapping in a pseudo-F1 population for grape PA composition

To our knowledge, this study presents the first QTL analysis on grape PA composition with comparisons between skin and seeds of grape berry. This is also the first work on grape using multiple QTL models taking into account both main effects and digenic epistasis during the mapping procedure. QTL mapping in animals has shown that epistasis effects are often large enough to be detected and thus merit a systematic scan regardless of population size, although larger populations (> 500 individuals) allow a more powerful epistasis detection [56]. By employing the multiple QTL mapping approach, we actually showed the important involvement of epistatic interaction in shaping PA composition variation; indeed, some loci were involved in phenotypic variation almost exclusively through pairwise interaction. Our mapping population is of sufficient size (191 individuals) to allow identification of small effect QTLs. However, one should keep in mind that the *R^2 ^*estimate of individual QTLs is usually overestimated [57] and may have a wide confidence interval [58]. Some of the identified QTLs may therefore be of smaller effect in reality. One should thus be cautious in result interpretation and further identification of causal polymorphism although we did check initially the genome-wide first type error rate.

Allele contribution to individual QTL was mainly due to additive effects between Syrah and/or Grenache alleles (Additional file 6). Parental detection allowed the identification of smaller additive QTLs because of a greater power due to more individuals in each genotypic class compared to consensus detection (i.e., 2 and 4 genotypic classes for parental and consensus detection, respectively). On the other hand, QTL detection on the consensus map allowed us to estimate QTL dominance effect s, i.e. the interaction between allelic classes, but not necessarily with the assumption of a dominant-recessive relationship [59]. In the present work, 9.8% and 30% of QTLs detected in skin and seeds, respectively, had dominance as the major allelic effect (D in Figure 4 and Additional file 6). For example, the locus 8@69 of concB in seed was involved in phenotypic variation almost exclusively through dominance (Additional file 6) and this information would have been overlooked if parental mapping only had been performed.

### Genetic architecture of grape PA composition

#### PA total content

QTL results were consistent with the results of PA variable correlation: no significant correlation was observed for concP between tissues and no co-locating QTL was identified for this variable between skin and seeds, while common QTLs were identified for both concB and concK across tissues. Since concB and concK take into account berry size- and yield-related traits, these co-located QTLs for concB and concK may be involved indirectly in PA total content through alteration of berry development or yield-related traits. Indeed, several QTLs for concK co-located with QTLs for yield-related traits, especially loci on chromosomes 8, 13, 17 and 18 where QTLs for yield related and berry size related-trais were also identified in the same S × G population (Doligez et al., unpublished data). Unlike these yield-related loci, the loci identified for concP, which were also identified for concB and concK could be specific targets for a better understanding of the contrasting PA content in berry compartments.

Association tests were in accordance with the involvement of *VvMYBPA2 *in PA content variation, as suggested by a previous study [50]. Two *VvMYBPA2 *SNPs were significantly associated with skin PA content variables. (Table 3). *VvMYBPA2 *is mainly expressed in berry skin at green stage and its overexpression in grape hairy root significantly increased PA production [50]. The significant associations of *VvMYBPA2 *were positioned in promoter and intron and might be involved either in transcription level alteration or through linkage disequilibrium with other causal mutations. A minor association was identified between a non-synonymous polymorphism of *VvCHI1 *and skin concP (Table 3). *VvCHI *encodes an upstream enzyme in the grape flavonoid pathway. This gene may be involved in PA content variation through the control of the flux of intermediate substrate. However, precise involvement of *VvCHI1 *in PA content variation needs further genetic and functional confirmation.

Among tested candidate genes, *VvMYB5a*, located on chromosome 8, is under several QTLs, especially total content-related QTLs. A previous physiological study showed that ectopic expression of *VvMYB5a *in tobacco induced expression of flavonoid genes and significantly increased both PA and anthocyanin production [49]. The authors therefore proposed *VvMYB5a *as an upstream regulator of flavonoid pathway. In our work, no significant association was found for *VvMYB5a *while the whole gene was sequenced (Table 2). Further investigations would be necessary to figure out if this gene is involved in grape PA content variation.

#### PA subunit synthesis: the hydroxylation patterns of flavan-3-ols

All flavonoids carry a hydroxyl group at the 4' position of B-ring (Figure 1). The flavonoid hydroxylation patterns of B-ring were first studied in ornamental plants for colour engineering because it is a major colour determinant for anthocyanins, another class of flavonoids sharing a similar C6-C3-C6 skeleton with PA monomeric subunits [60,61]. Links between *F3'H *and *F3'5'H *gene activities and their relative flavan-3-ols are less obvious due to the lack of easily assessed reporters. Our results for grape skin variables showed that five genomic regions (on chromosomes 3, 6, 8, 10 and 18) harboured co-located QTLs for epiEx, egcEx and F3pr35. This co-localisation is not surprising since epiEx and egcEx were the major components for F3pr35 variable construction and were therefore highly correlated (Figure 2). These loci are probably involved in the flux between di-hydroxylated and tri-hydroxylated PA building blocks. An interesting point is that the QTL on chromosome 6 for both egcEx and F3pr35 co-located with a genomic region corresponding to the *F3'5'H *gene family.

However, no significant association was detected between the two tested *VvF3'5'H *isogenes and hydroxylation pattern variables in this work. *F3'5'H *is present as a multigenic family in the grapevine genome in which at least 15 isogenes have been identified [62]. For duplicated genes, neofunctionalisation and/or subfunctionalisation could conduct to specialisation of each isoform in a spatio-temporal manner [63-65]. Actually, the isogene *VvF3'5'H 1.1 *(or *VvF3'5'H n *in [62]) was shown to be expressed only in vegetative organs, while *VvF3'5'H 2.1 *(or *VvF3'5'H f *in [62]) is expressed in berry skin. The assessment of the polymorphisms of all isogenes may give more insights for links between *F3'5'H *and hydroxylation variation.

#### PA subunit synthesis: the galloylated flavan-3-ols

To date, the underlying genetic determinism for the production of PA galloylated building blocks is still unclear. Our results in both grape berry skin and seeds showed that the quantitative variation of (-)-epicatechin-3-*O*-gallate was probably under the control of many genomic regions and digenic epistatic interactions (Figures 4 and Additional file 6). Additional information was provided by association tests which revealed 4 weak but significant associations between galT in seeds and SNPs of *VvMYBPA2*. These associated SNPs are located in introns or in C-terminal of the proteins which could contain protein-protein interaction domains [66] (Table 2). This result suggests that the associated SNPs might lead to alteration of transcriptional complex recruitment or interaction with other proteins. [50]. Glucosyltransferases were recently identified as putative candidates involved in the first enzymatic step of PA galloylation [67]. Since they are located on chromosome 3 where QTLs for galEx in skin and seed are positioned (Figure 4 and Additional file 7), they may be good candidates to be tested by association genetics in the next future.

#### PA subunit synthesis: the trans- and cis- subunits

Synthesis of PA *trans*-and *cis*-subunits is tightly related to PA polymerisation since intermediate substrates in the flavonoid pathway are assumed to take up a *trans*-configuration while major extension subunits assume a *cis*-configuration (e.g. (-)-epicatechin) [68]. Major advances in understanding PA subunits biosynthesis were made through the isolation of two genes coding for specific enzyme activities for the formation of terminal/monomers: 2,3-*trans*-(gallo)catechin and 2,3-*cis*-epi(gallo)catechin [3-5]. Recently, another dynamic view of the flux between *trans-*and *cis-*terminal units/monomers was provided by Gargouri and co-workers who demonstrated the ability of grape ANR to epimerise (+)-catechin to (-)-epicatechin [69]. Our results seem to be in accordance with this work since a major locus on chromosome 17 was identified for catT and epiT, the two chiral flavan-3-ols, which was also the major locus for Ftranscis_T and Ftranscis_all in skin. This locus furthermore co-located with *VvLAR2*, an isogene of LAR, which belongs to the Reductase-Epimerase-Dehydrogenase (RED) family, as ANR, and thus might display both epimerase and reductase activity. Similarly, three *VvLAR1 *SNPs were significantly associated to Ftranscis_all in skin and therefore merit further functional investigation to understand its involvement in *in vivo *PA subunit synthesis.

On the other hand, the origin of extension subunits is still uncertain: are extension subunits derived from intermediate substrates in the pathway or from end products such as (-)-epicatechin and (+)-catechin? [1,68,70]. In our work, significant correlation was not systematically observed for subunits of the same nature but differing in position in the polymer, and few QTLs co-located. In addition, the QTLs for flux between *trans-*and *cis-*subunits were most often different between extension position (Ftranscis_Ex) and terminal subunits/monomes (Ftranscis_T). All these results argue in favour of the involvement of different loci in PA building blocks synthesis and in the control of flux between *trans*-and *cis*-subunits according to their position in the polymer. Stafford *et al*. already suggested from radioactive labelling experiments that upper and lower units arise from different steps of the pathway rather than from the condensation of similar units [71].

#### PA polymerisation

An aspect of the debate about PA polymerisation concerns the enzymatic or nonenzymatic polymerisation (reviewed by [1,70]). The existence of a polymerase is supported by the barley PA mutant *ant26*, containing amounts of (+)-catechin equivalent to wild-type content but only trace amounts of PAs [72]. On the other hand, *in vitro *chemical synthesis of PAs has also been reported [73] and these authors observed a modulation of polymer size through the modulation of the relative amounts of extension unit intermediates and monomers. One can thus hypothesise that instead of a polymerase, the *ant26 *mutation could directly affect the suitable conditions for spontaneous PA polymerisation, such as appropriate pH [70]. Further investigation of the QTLs identified in this study would bring more insights into this polymerisation issue. Indeed, in the case of skin mDP, *H^2 ^*was high (0.82) and the multiple QTL model accounted for 70% of the genotypic variance, corresponding to 57% of the total phenotypic variance. The largest QTL on chromosome 17 explained alone 55% of genotypic variance and was also the major locus for seed mDP (Figure 5F). This QTL is therefore an interesting target for mDP genetic mechanism investigation. Another mDP QTL located on chromosome 1 might also be an interesting target for understanding PA polymerisation. This QTL co-located with a gene encoding a PA-specific synthetic enzyme, *VvLAR1*, for which several SNPs in linkage disequilibrium (data not presented) were significantly associated to mDP and catT in skin, consistent with the corresponding QTL. Interestingly, *VvLAR1 *is highly polymorphic in Grenache while almost homozygous in Syrah (1 SNP in the coding region, data not presented), in accordance with the fact that the QTL for mDP in this region is mainly due to a Grenache allelic effect.

### Tissue-specific genetic architecture for PA composition

In accordance with a previous study which demonstrated the tissue specificity of transcriptional profiles in grape berry [74], the present work illustrates different genetic mechanisms for grape PA composition between skin and seeds: QTLs differed in terms of number, position, *R^2 ^*and allelic effects. For total content variables, the major QTLs differed in skin and seeds. For subunit percentage and composite variables, important differences between tissues were also observed. Globally, for the same PA subunit percentage variables in both tissues, only 5 QTLs among 74 had overlapping intervals between skin and seeds. Another contrasting feature was observed: in skin, 54% of all QTLs accounted for Syrah additive effect and 78% for Grenache effect whereas in seeds, 74% of all QTLs accounted for Syrah additive effect and 53% for Grenache effect (Additional file 6 and Figure 4). Even for loci identified in both tissues for a given variable, major allelic effect and *R^2 ^*differed (e.g. the QTL in chromosome 17 for catT, Additional file 6). Our results suggest that seed PA variation is controlled by QTLs of moderate and equivalent magnitude with involvement of epistasis. On the other hand, skin variables are mainly under the genetic control of a few large effect loci with a fluctuating variance unexplained by QTLs.

The different genetic architectures between tissues could result from divergent functional evolution of PAs in these two berry compartments. For fruits in general, ensuring protection of the embryos is essential. Because of their abundance and their ability to protect plants against biotic stresses, PAs and flavan-3-ols might be the major molecules involved in grape embryo protection. In fact, their influence in maintaining seed dormancy has been demonstrated in Arabidopsis [75] and their interaction with phytohormones has also been reported [76,77]. Therefore, to prevent biological fluctuation due to a single polymorphism mutation, a network with multiple cross-talking actors as a product of evolution without human selection could be postulated in the case of seed PAs, as suggested by the identification of numerous small effect QTLs and the involvement of epistasis. Conversely, skin is the first protective barrier of the grape berry against its environment. In plants, PAs are thought to be involved in self-defence mechanisms [1]. For berry consumers, skin PAs confer flavour to berries and are also responsible for major organoleptic qualities of wine. Consumers in turn could help the plant in seed dispersion or vegetative propagation. However, high quantities of PAs would confer to berries too much astringency and bitterness, which would lead consumers (specially humans) to reject grapevines producing such berries for direct consumption (or wine-making). Human selection in particular could therefore have narrowed down the genetic basis of skin PAs over time and consequently led to a specific genetic architecture with a few large effect QTLs for skin PA variables.

### QTL mapping and association analysis as complementary approaches for candidate locus identification

In this work, we used both QTL mapping and association analyses to identify phenotype-marker associations. For identification of phenotype-associated markers, grapevine segregating populations have a greater diversity than populations derived from inbred lines due to heterozygous parental cultivars. However, their genetic background remains relatively narrow compared to diversity panels. On the other hand, QTL mapping may reveal associations undetected in diversity panels due to low allelic frequency. The inconsistency between both approaches sometimes encountered in this work could therefore result from the fact that the available genetic polymorphisms were different between the two populations: causal polymorphisms in one population might be monomorphic in the other. In addition, our analysis focused on genes of known function co-locating with QTL while other candidates could underlie QTL intervals. Besides time-consuming fine mapping, candidate genes can also be selected by combining QTL results with other data such as transcriptomics. Nevertheless, *LAR1 *gene evoked a particular interest through association test. Complementing functional studies performed on a single cultivar [14], we provide here additional confirmation of *LAR1 *gene involvement in grape PA composition through a diversity panel study.

## Conclusions

The present work confirmed presumptions about the complex genetic architecture of PA composition in grape berries. QTLs for PA total content, PA building blocks, degree of polymerisation and ratio between building blocks were identified. Berry PA composition offers a case study for tissue-specific genetic architecture: in skin, the same major loci were involved in several PA variables while multiple and moderate QTLs with strong epistasis were the principal genetic factors for seed PA composition. These differences might be due to human selection on skin PA leading to a reduced diversity of related genes while a multi-factor network controlling seed PA synthesis would be necessary to protect grapevine embryos. Association tests confirmed the interest of *VvLAR1 *as a candidate gene in modulating catT and mDP in berry skin, as well as *VvMYBPA2 *for total content and probably for subunit galloylation. This study provides the first assessment of genetic mechanism underlying grape PA composition which opens doors for further PA genetic studies.

## Abbreviations

ANR: Anthocyanidin reductase; BLUP: Best linear unbiased predictor; 4CL: 4-coumaroyl CoA ligase; C4H: Cinnamate 4-hydroxylase; catEx: (+)-catechin extension subunits; catT: (+)-catechin terminal subunits/monomers; CHI: Chalcone isomerase; CHS: Chalcone synthasel; concP: Total content in mg/g fresh weight; concB: Total content in mg/berry; concK: Total content in mg/kg berries; DFR: Dihydroflavonol reductase; egcEx: (-)-epigallocatechin extension units; epiEx: (-)-epicatechin extension subunits; epiT: (-)-epicatechin terminal subunits/monomers; F3H: Flavanone 3-hydroxylase; F3'H: Flavonoid 3' hydroxylase; F3'5'H: Flavonoid 3'-5' hydroxylase; galEx: (-)-epicatechin-3-*O*-gallate extension subunits; galT: (-)-epicatechin-3-*O*-gallate terminal subunits/monomers; LOD: Logarithm of odds; LDOX: Leucoanthocyanidin dioxygenase; LAR: Leucoanthocyanidin reductase; PA: Proanthocyanidin; PAL: Phenylalanine ammonia-lyse; QTL: Quantitative trait locus; SNP: Single nucleotide polymorphism; SSR: Simple sequence repeat.

## Authors' contributions

YFH carried out the gene sequencing and alignment, performed data analyses, prepared tables and figures and drafted the manuscript. AD checked phenotyping and genotyping data, performed linkage map construction and first statistical analysis and participated in manuscript preparation. AFL participated in gene sequencing, sequence alignment, association test analysis and in manuscript preparation. LLC participated in upstream data analyses, data interpretation and in manuscript preparation. YB conducted field experimentation and sample collection. AC performed genotyping for linkage map construction. FV, VM, CM, and JMS carried out biochemical analysis. NT directed PA variable conception, interpreted the result and participated in manuscript preparation. VC and PT conceived the study, participated in its design, coordination, data interpretation and manuscript preparation. All authors read and approved the final manuscript.

## Supplementary Material

Additional file 1 **Primers used for the amplification and sequencing of the candidate genes**. Click here for file

Additional file 2 **Distribution of residuals and BLUPs and Quantile-quantile plot of residual and BLUPs of the best fitted model for S × G population**. For each PA variable, 4 panels are shown: distribution of residuals of the best fitted model (box-and-whisker plot, topleft), quantile-quantile plot of model residuals against a theoretical normal distribution (topright), distribution of BLUPs of the best fitted model (box-and-whisker plot, bottomleft), quantile-quantile plot of BLUPs against a theoretical normal distribution (bottomright). Click here for file

Additional file 3 **Phenotypic data analysis and best fitted models for variance component estimation**. Analysis method and effects included in the best fitted model. Click here for file

Additional file 4 **Effect of minor genotypic frequency and non-normalty of observed phenotype on the association test**. Two sections are in this file. 1. Test for the enrichment of low frequency polymorphisms among associated markers. 2. Test for the effect of the non-normalty of the trait in the association tests. Click here for file

Additional file 5 **Summary of PA variable distributions and broad sense heritability (*H^2^*) in S × G and CC populations**. Two tables inside: Table A, summary of S × G population; Table B, summary of CC population. Skin data were collected in 2005 and 2006 and seed data were collected in 2006 and 2007. Parental values are indicated as mean ± standard error. Broad sense heritability (*H^2^*) was estimated based on the best fitted model as the percentage of phenotypic variance explained by the genotypic variance. Click here for file

Additional file 6 **QTL summary for consensus and parental detection**. Summary for consensus detection and parental detection are in two separate sheets. Term: main effect QTLs and pairwise epistatic interactions. Main effect QTLs are indicated by chromosome@position of LOD peak while interaction terms are indicated by ":" linking main effect QTLs. Map (for QTL summary of parental detection): the parental map used for QTL detection. LOD score and *R^2 ^*were estimated by dropping the considered term from the full model. For loci involved in pairwise interaction, their LOD and *R^2 ^*were estimated by dropping both the main effect and the associated interaction effect. df: degree of freedom dropped for QTL effect estimation. Type III SS: type III sum of squares. CI: LOD-1 confidence interval. For consensus detection, As, Ag and D indicate additive effect from Syrah alleles, additive effect from Grenache alleles and dominance effect which were estimated according to Segura et al. [41]: As: 1/4[(μ_ad_+μ_ac_)-(μ_bd_+μ_bc_)], Ag: 1/4[(μ_ac_+μ_bc_)-(μ_ad_+μ_bd_)], D: 1/4[(μ_ac_+μ_bd_)-(μ_bc_+μ_ad_)], where μ_bd_, μ_bc _and μ_ad _are phenotypic means for corresponding genotypes relative to phenotypic mean of ac genotype. Thus here, μ_ac _= 0. effect column in QTL summary for consensus detection indicates major effects of the considered locus involved in phenotype variation, satisfying the following condition: (|As| or |Ag| or |D|)/(|As|+|Ag|+|D|) > 0.30. "Estimate" column in QTL summary for parental detection gives the allelic effect between parental alleles or the corresponding interaction effect, the sign is arbitrary. The type III sum of squares, df, LOD and *R^2 ^*of the QTL model are indicated at the last row of each variables, highlighted on yellow. The loci identified in both skin and seeds for the same variable (*i.e*. having overlapping LOD-1 confidence interval) are highlighted in pink. Click here for file

Additional file 7 **QTL maps with positioned grape PA candidate genes**. Parental and consensus maps are presented in parallel: left, Syrah map, indicated by S; centre, consensus map, indicated by C and right, Grenache map, indicated by G. QTLs are presented as vertical lines at the right side of each map: the line length corresponds to LOD-1 confidence interval and the LOD peak is indicated by a small horizontal bar in the confidence interval. Known candidate genes are positioned between flanking markers, indicated by red-filled bars, according to 12X grape genome sequence (http://www.genoscope.cns.fr). Red-filled bars indicated flanking marker interval of regulatory genes and green-filled bars for enzyme-coding genes. Click here for file

Additional file 8 **Model comparison prior to association analyses**. -2lnlikelihood is shown. Model comparison was performed by likelihood ratio comparing each model to the most complete model. Significance was assessed using the *χ^2 ^*distribution with degree of freedom as the difference in the number of parameter between two models. Significance level is indicated as *, *P *< 0.05, **, *P *< 0.01, ***, *P *< 0.001. Click here for file
