# Supplementary material for: Dissecting genetic architecture of grape proanthocyanidin composition through quantitative trait locus mapping
Source: BMC Plant Biol. 2012 Feb 27;12:30. doi: 10.1186/1471-2229-12-30 (PMC3312867; doi:10.1186/1471-2229-12-30)
Supplement: Additional file 1 — Primers used for the amplification and sequencing of the candidate genes. [file 1471-2229-12-30-S1.PDF]

Additional file 1: Primers used in this study

Amplicon	Forward primer (5'-3')	Backward primer (5'-3')	Amplicon size
CHI1	ACGACGATTTTGCCTTTGAC	CAGGAACAAGAGGAACCAACA	640 bp
CHI2	TGGCAGCAGATGACAAATATG	ACCAAAGCTCGTACAAGGACA	840 bp
DFR	CAAGCTGCATGGAAGTATGC	TTGGGCCATTCCGTTTTATTA	900 bp
F3'5'H1.1	TTGTTGGTCAATGGTCTTTA	ATAGTAGTGGTGGCAGTGGT	2850 bp
F3'5'H2.1	GCCATCCTCCCAACTTAT	TGGCTTGGTGGTAGAATG	2300 bp
LAR1_fragment1	CTCCGTGTGCTGTGTACTTG	CAACCCGTCTCCTTCTTCTG	900 bp
LAR1_fragment2	AGTTCACAGCCGAGATGCTT	CCACAAATTGCCTCCCTAGA	900 bp
Myb5a	CGGATATGACTGGCTGGATT	TTCACATACGATATTCACAC	1500 bp
MYBPA1	TTCCACATAATCCATGGTC	TTAACACAAATGTACATCGC	1620 bp
MYBPA2_fragment1	TAATGGATTTGGTGGACC	AAGCATGGACATCCCTGA	1300 bp
MYBPA2_fragment2	TATTCAACTTTCCCTGCC	CCAATCAGCCATAATGGTGTCT	850 bp
