# Supplementary material for: Dissecting genetic architecture of grape proanthocyanidin composition through quantitative trait locus mapping
Source: BMC Plant Biol. 2012 Feb 27;12:30. doi: 10.1186/1471-2229-12-30 (PMC3312867; doi:10.1186/1471-2229-12-30)
Supplement: Additional file 2 — Distribution of residuals and BLUPs and Quantile-quantile plot of residual and BLUPs of the best fitted model for S × G population. For each PA variable, 4 panels are shown: distribution of residuals of the best fitted model (box-and-whisker plot, topleft), quantile-quantile plot of model residuals against a theoretical normal distribution (topright), distribution of BLUPs of the best fitted model (box-and-whisker plot, bottomleft), quantile-quantile plot of BLUPs against a theoretical normal distribution (bottomright). [file 1471-2229-12-30-S2.PDF]

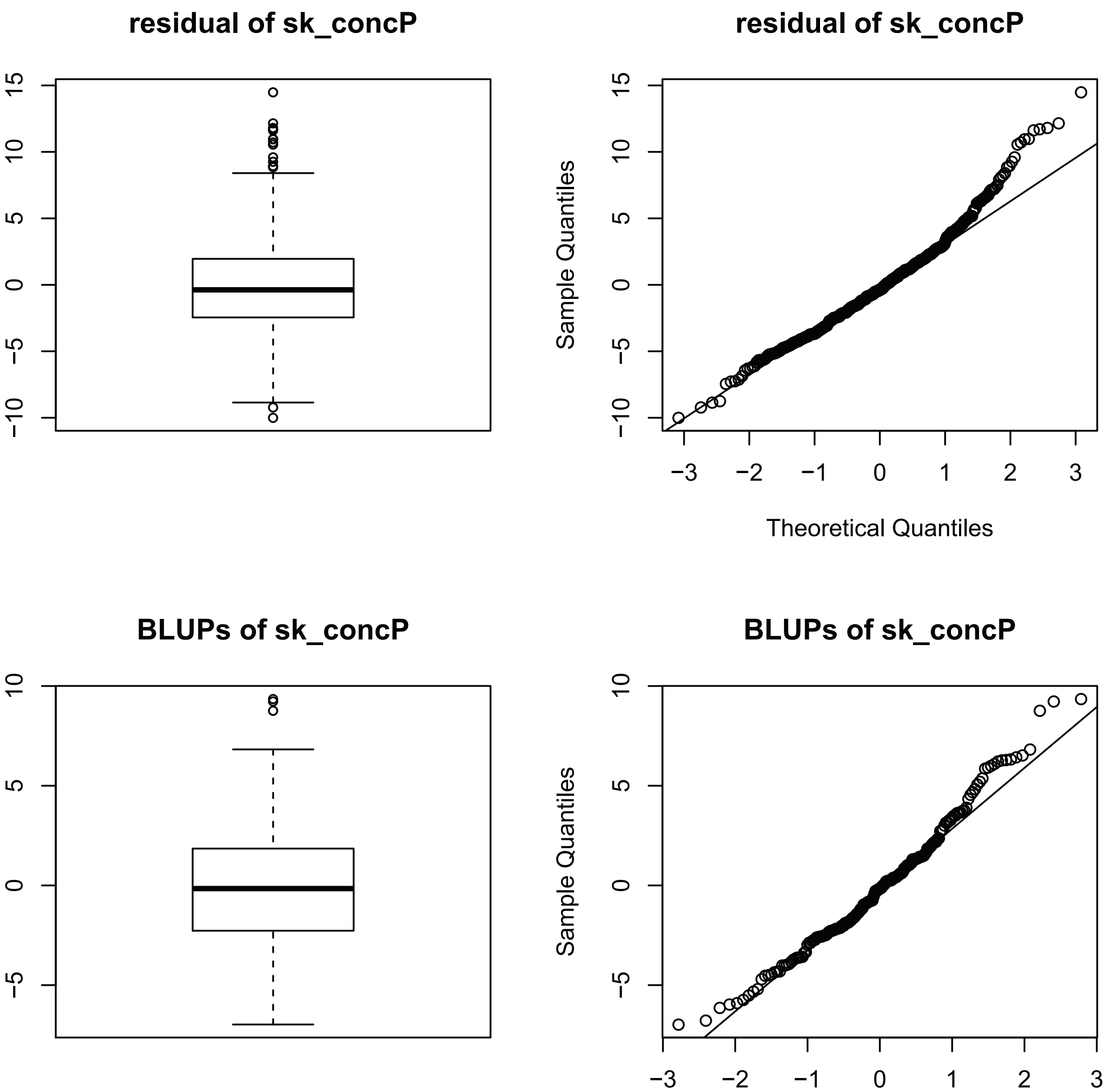

residual of sk_concP
residual of sk_concP
Sample Quantiles
Theoretical Quantiles
BLUPs of sk_concP
BLUPs of sk_concP
Sample Quantiles
Theoretical Quantiles

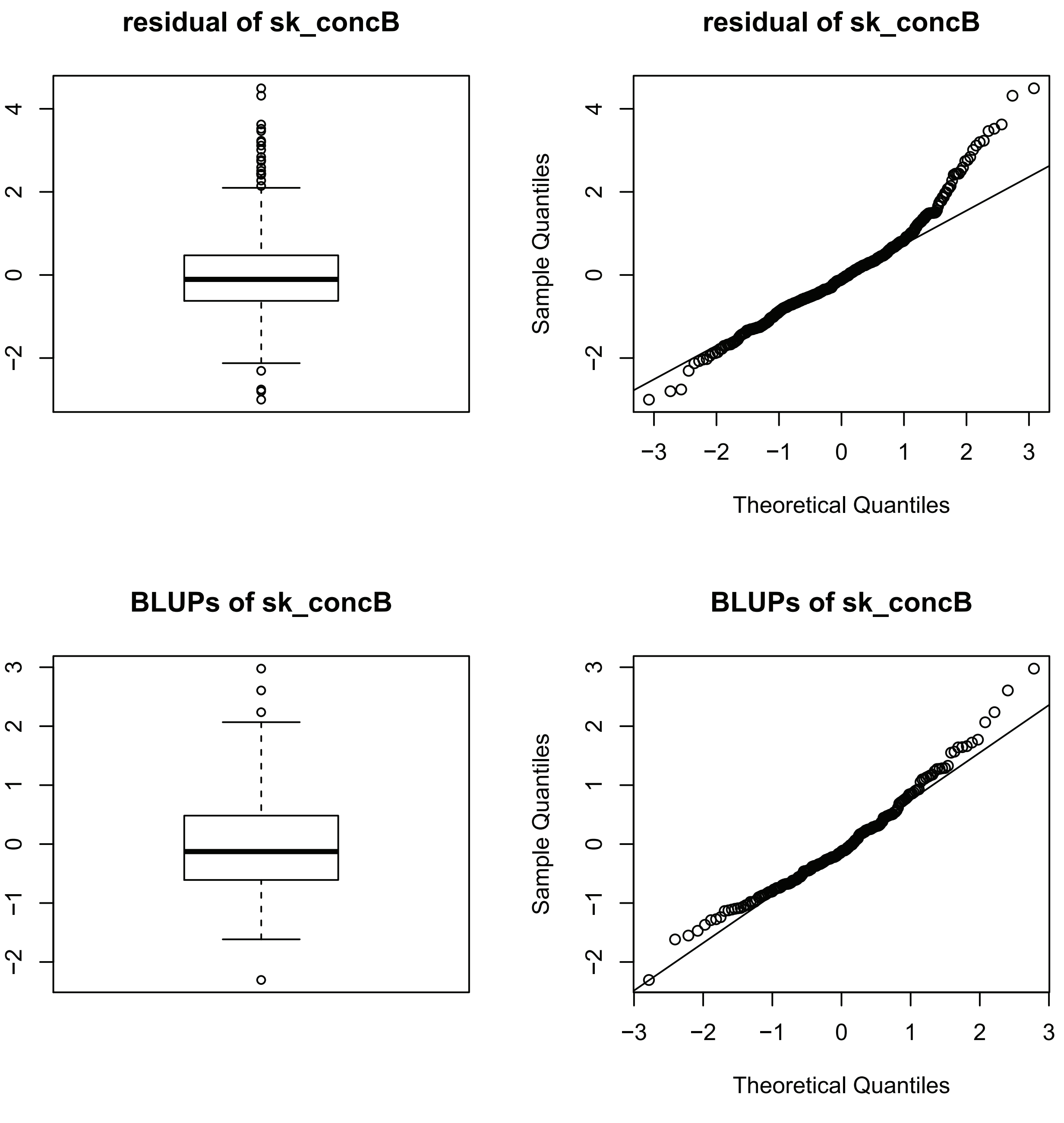
residual of sk_concB
residual of sk_concB
Sample Quantiles
Theoretical Quantiles
BLUPs of sk_concB
BLUPs of sk_concB
Sample Quantiles
Theoretical Quantiles

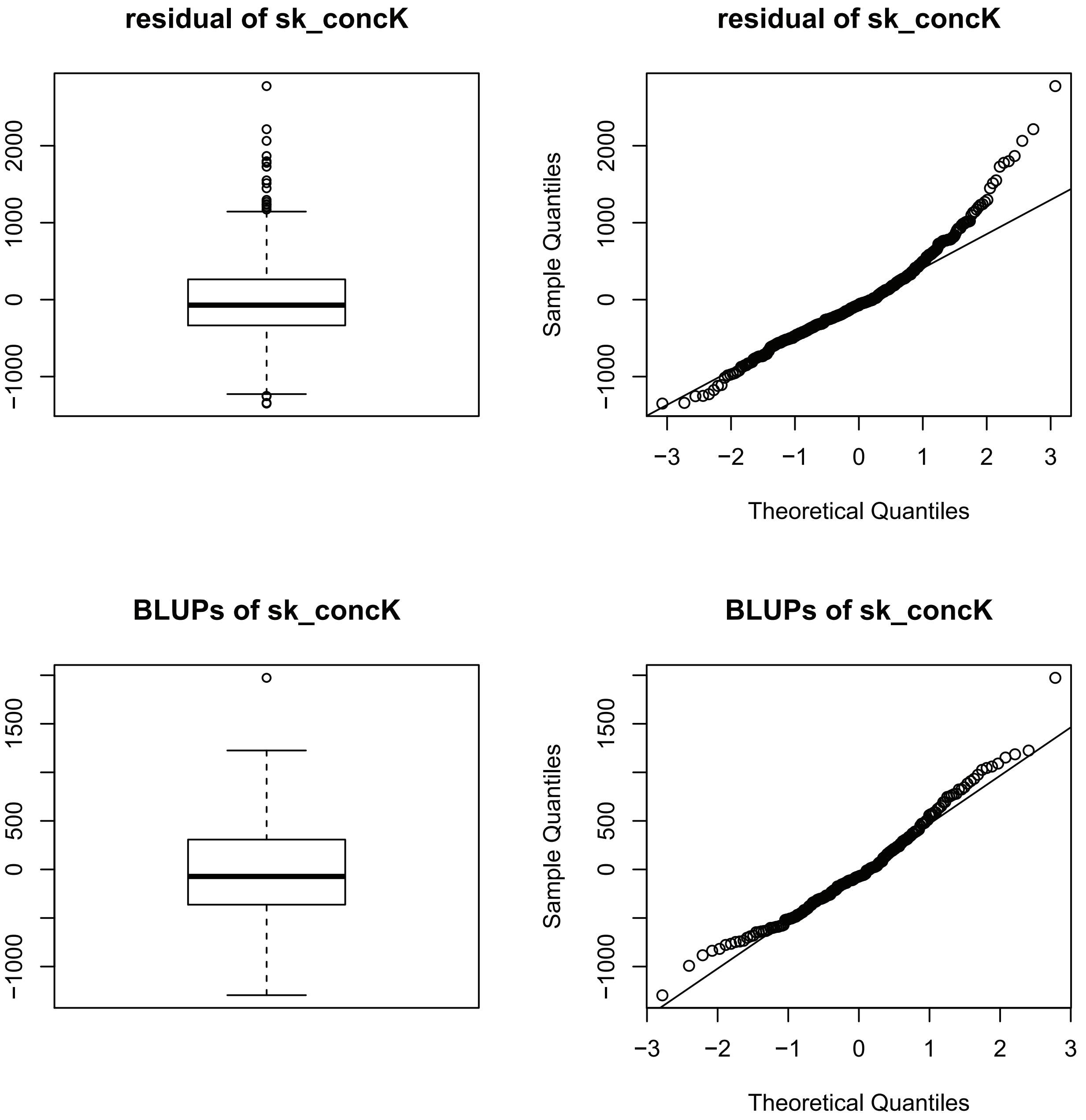
residual of sk_concK
residual of sk_concK
Sample Quantiles
Theoretical Quantiles
BLUPs of sk_concK
BLUPs of sk_concK
Sample Quantiles
Theoretical Quantiles

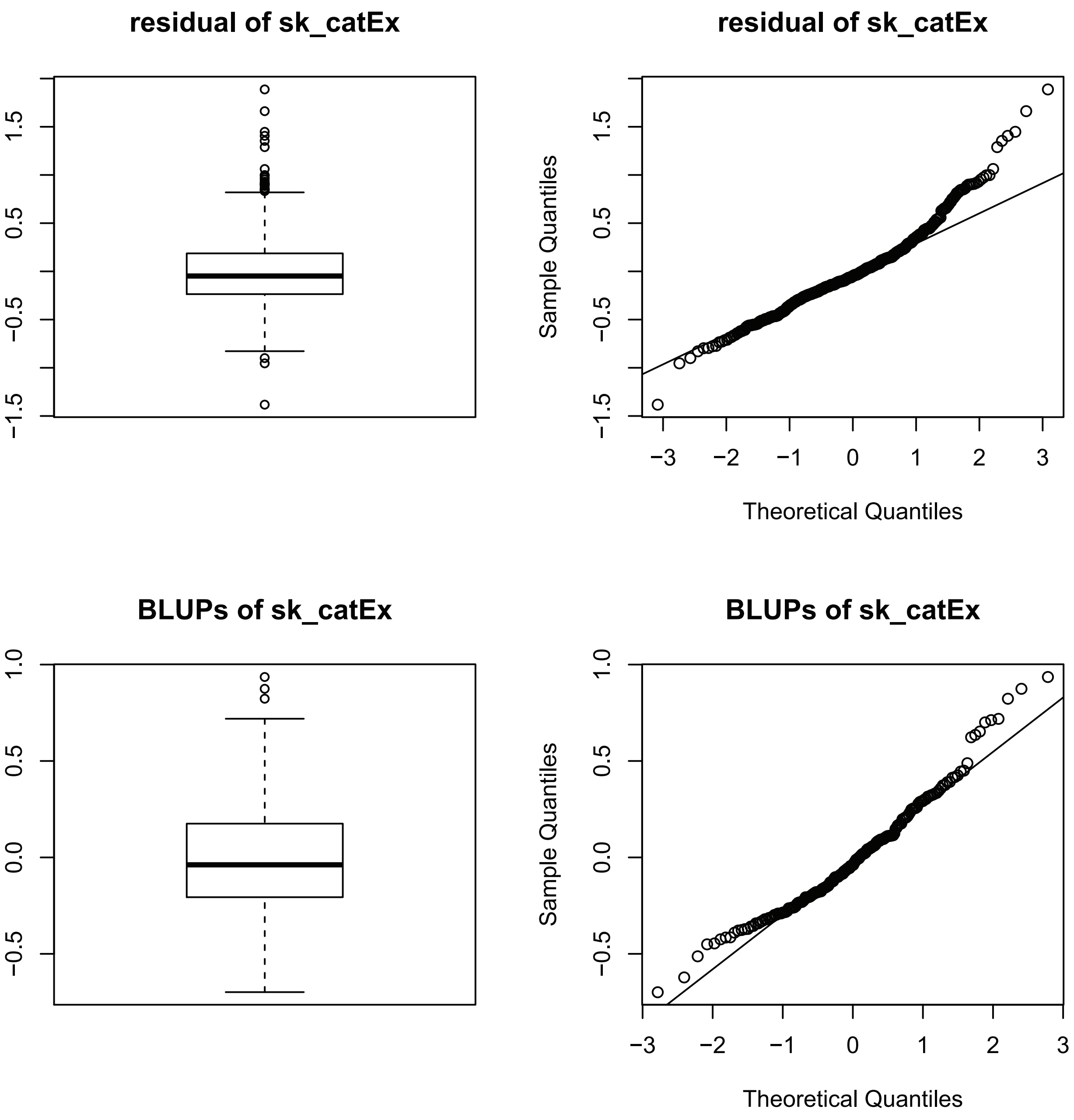
residual of sk_catEx
residual of sk_catEx
Sample Quantiles
Theoretical Quantiles
BLUPs of sk_catEx
BLUPs of sk_catEx
Sample Quantiles
Theoretical Quantiles

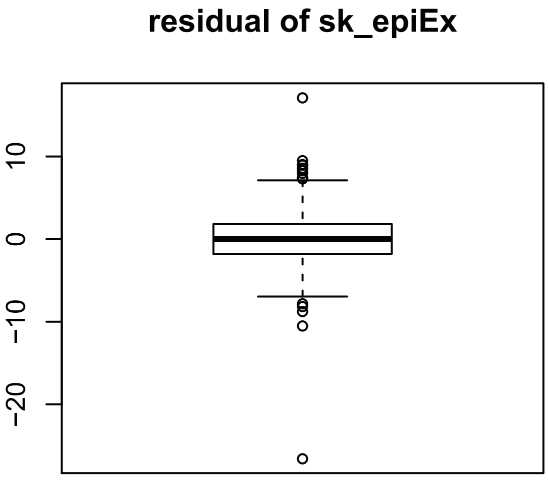
residual of sk_epiEx
10
0
−10
−20

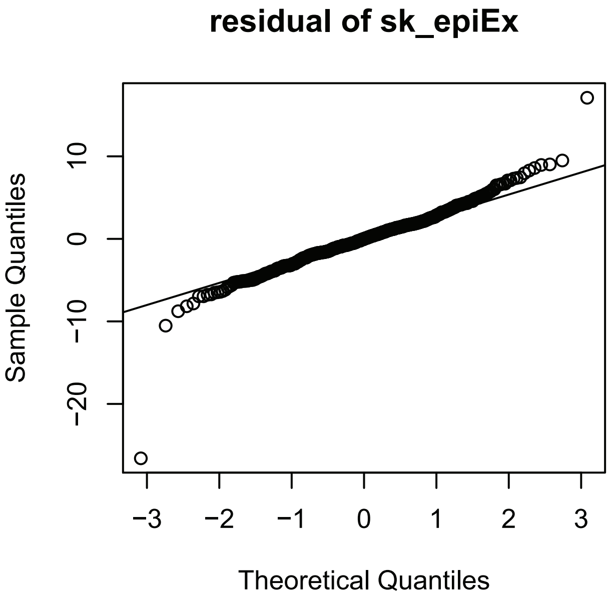
residual of sk_epiEx
Sample Quantiles
Theoretical Quantiles
10
0
−10
−20
−3
−2
−1
0
1
2
3

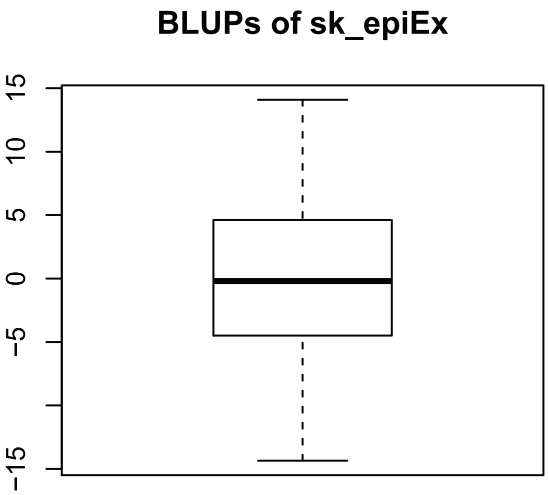
BLUPs of sk_epiEx
15
10
5
0
−5
−15

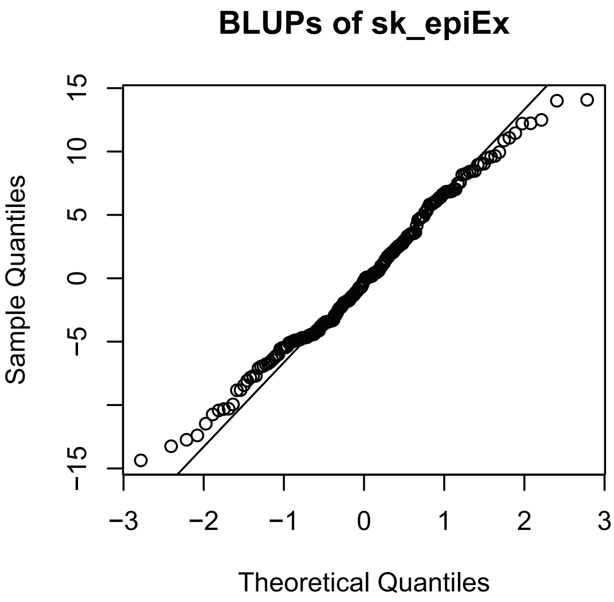
BLUPs of sk_epiEx
Sample Quantiles
Theoretical Quantiles
15
10
5
0
−5
−15
−3
−2
−1
0
1
2
3

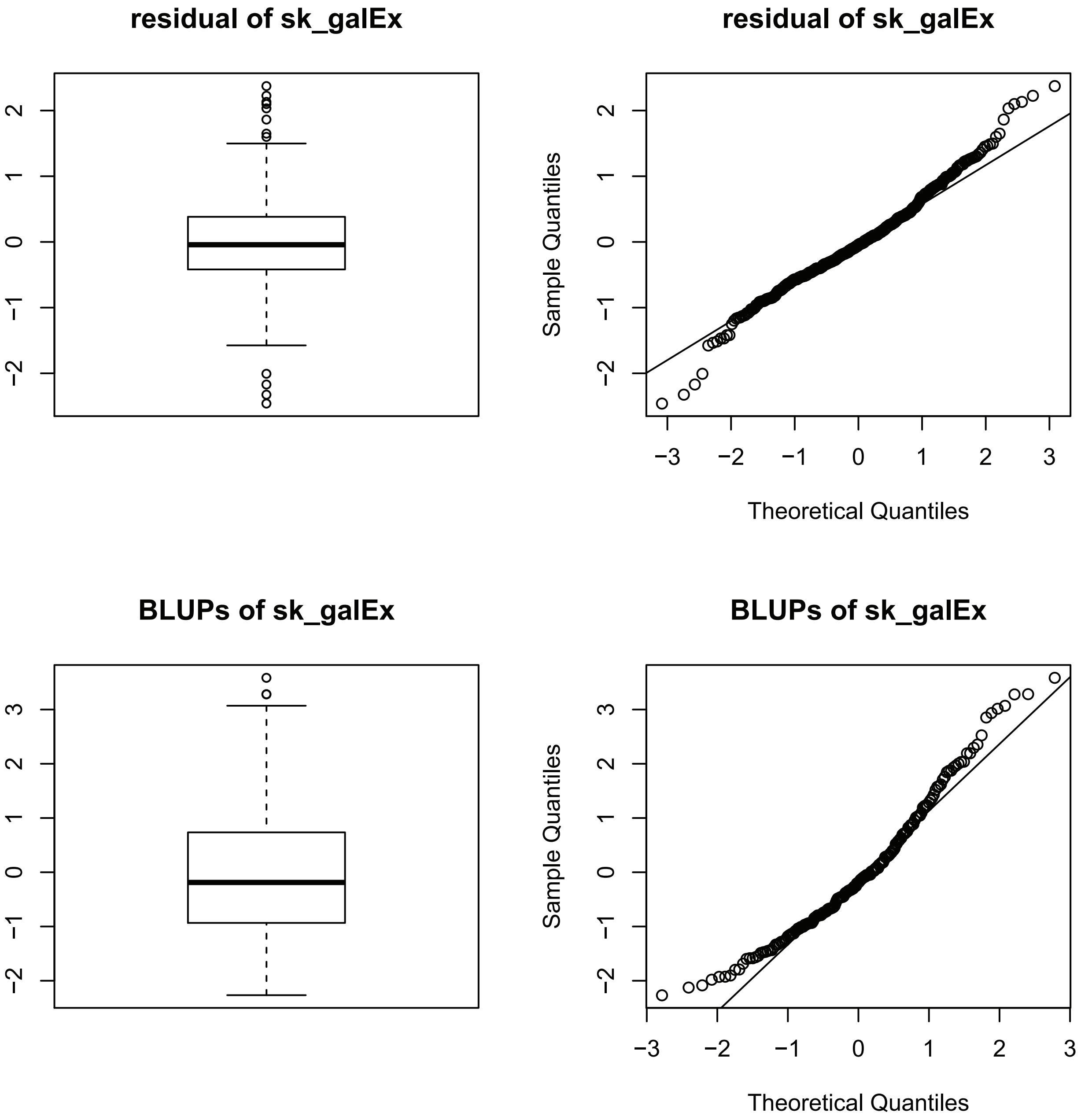
residual of sk_galEx
residual of sk_galEx
Sample Quantiles
Theoretical Quantiles
BLUPs of sk_galEx
BLUPs of sk_galEx
Sample Quantiles
Theoretical Quantiles

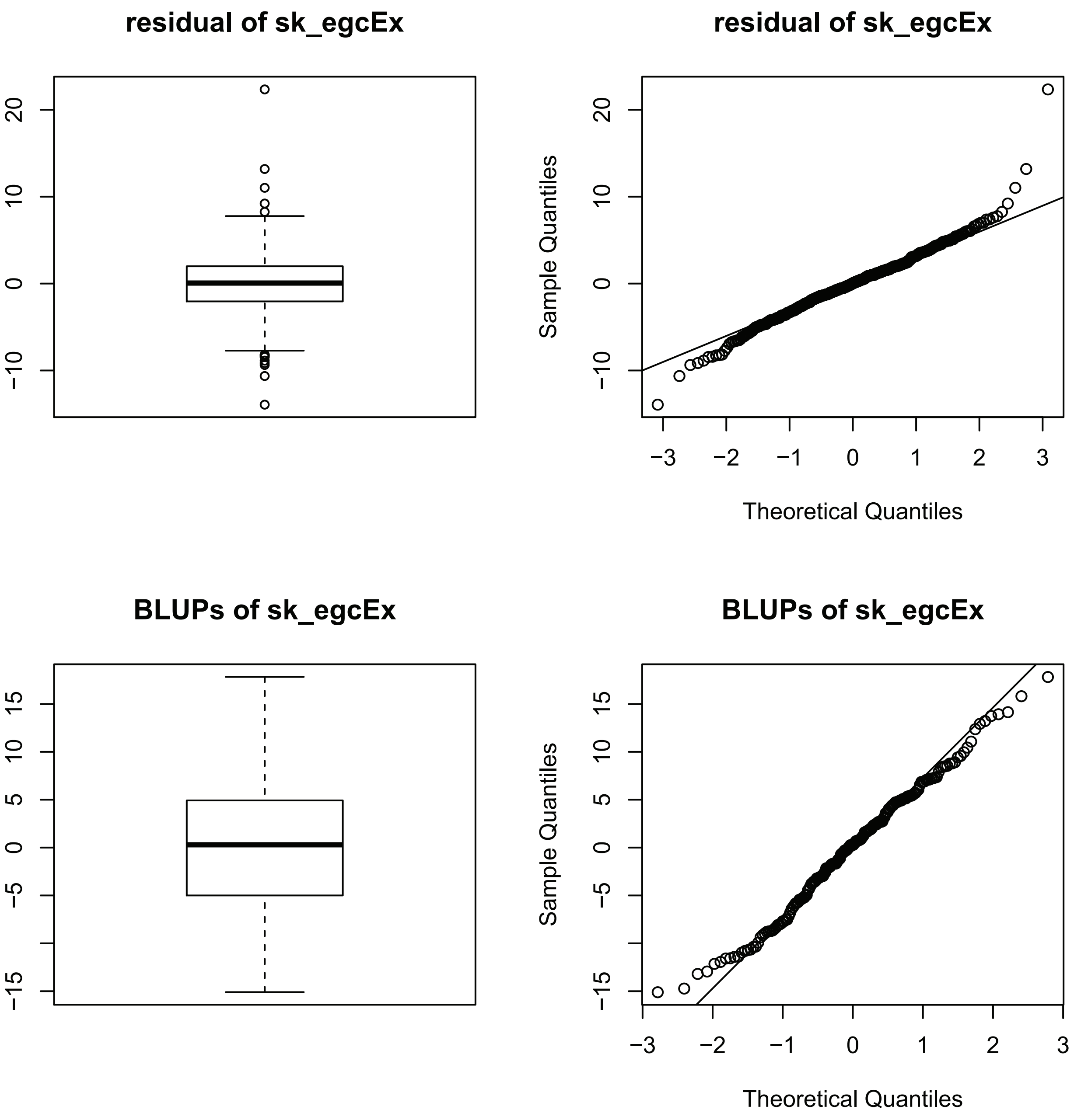

residual of sk_egcEx
residual of sk_egcEx
Sample Quantiles
Theoretical Quantiles
BLUPs of sk_egcEx
BLUPs of sk_egcEx
Sample Quantiles
Theoretical Quantiles

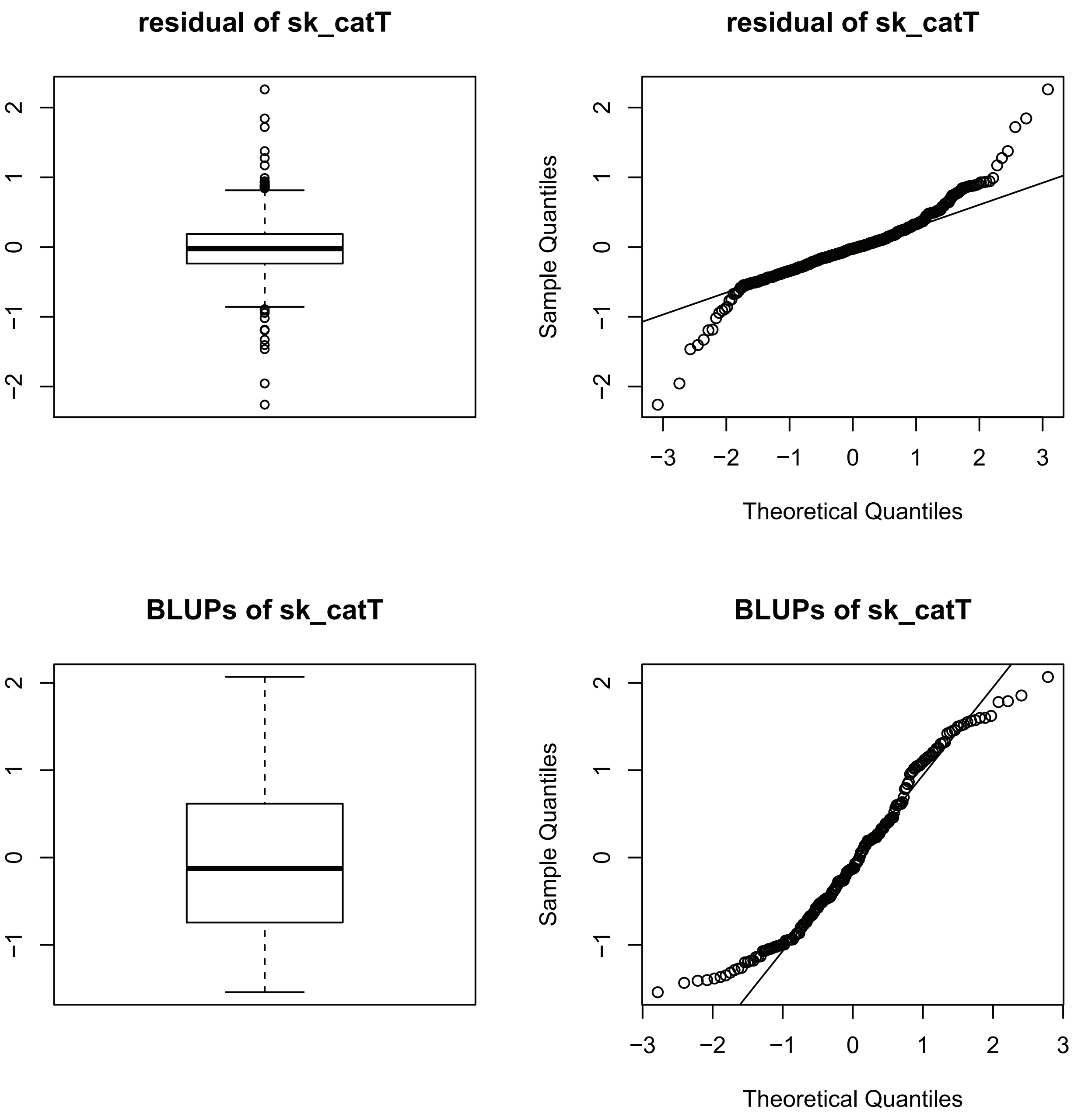

residual of sk_catT
residual of sk_catT
Sample Quantiles
Theoretical Quantiles
BLUPs of sk_catT
BLUPs of sk_catT
Sample Quantiles
Theoretical Quantiles

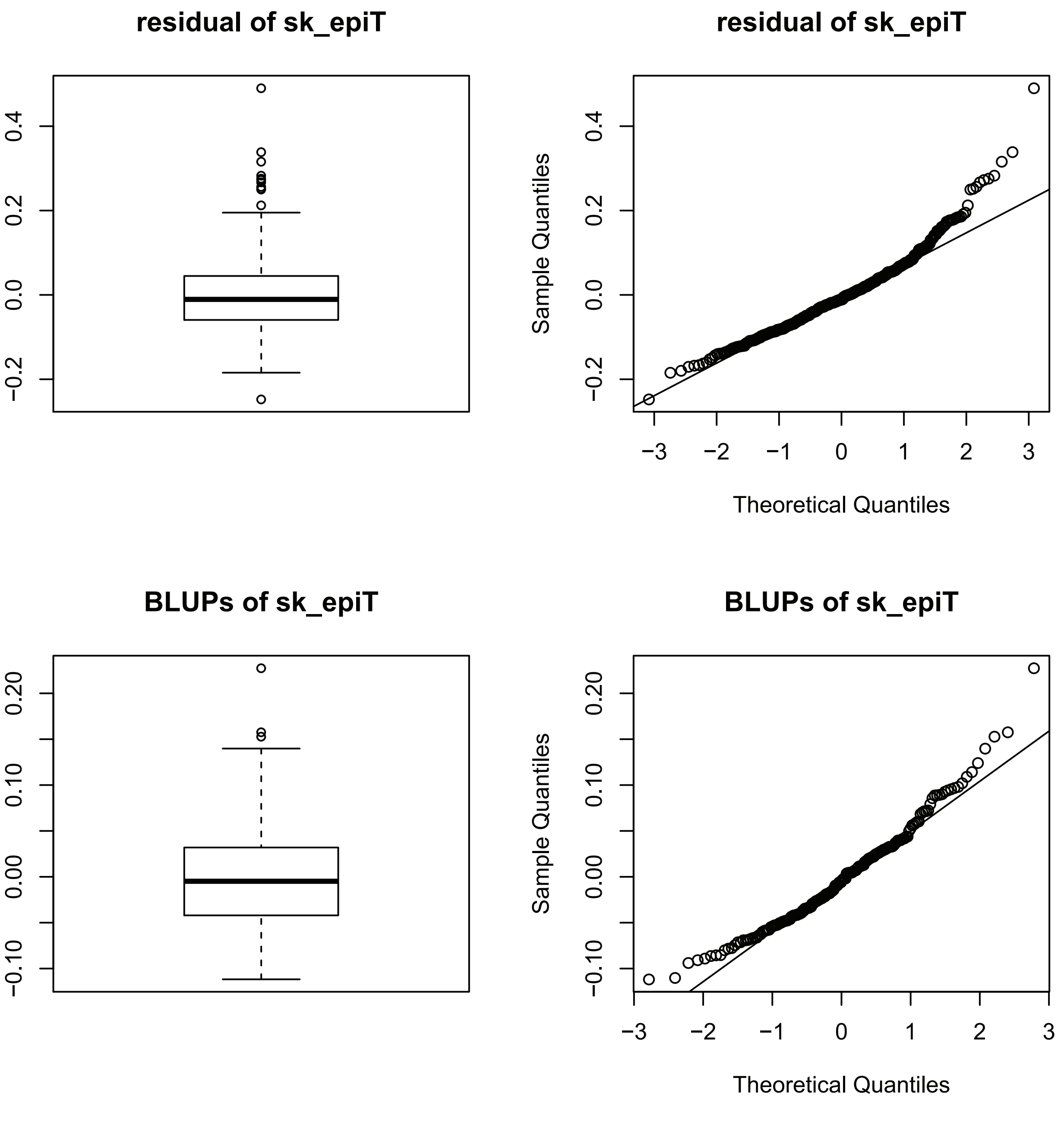
residual of sk_epiT
residual of sk_epiT
Sample Quantiles
Theoretical Quantiles
BLUPs of sk_epiT
BLUPs of sk_epiT
Sample Quantiles
Theoretical Quantiles

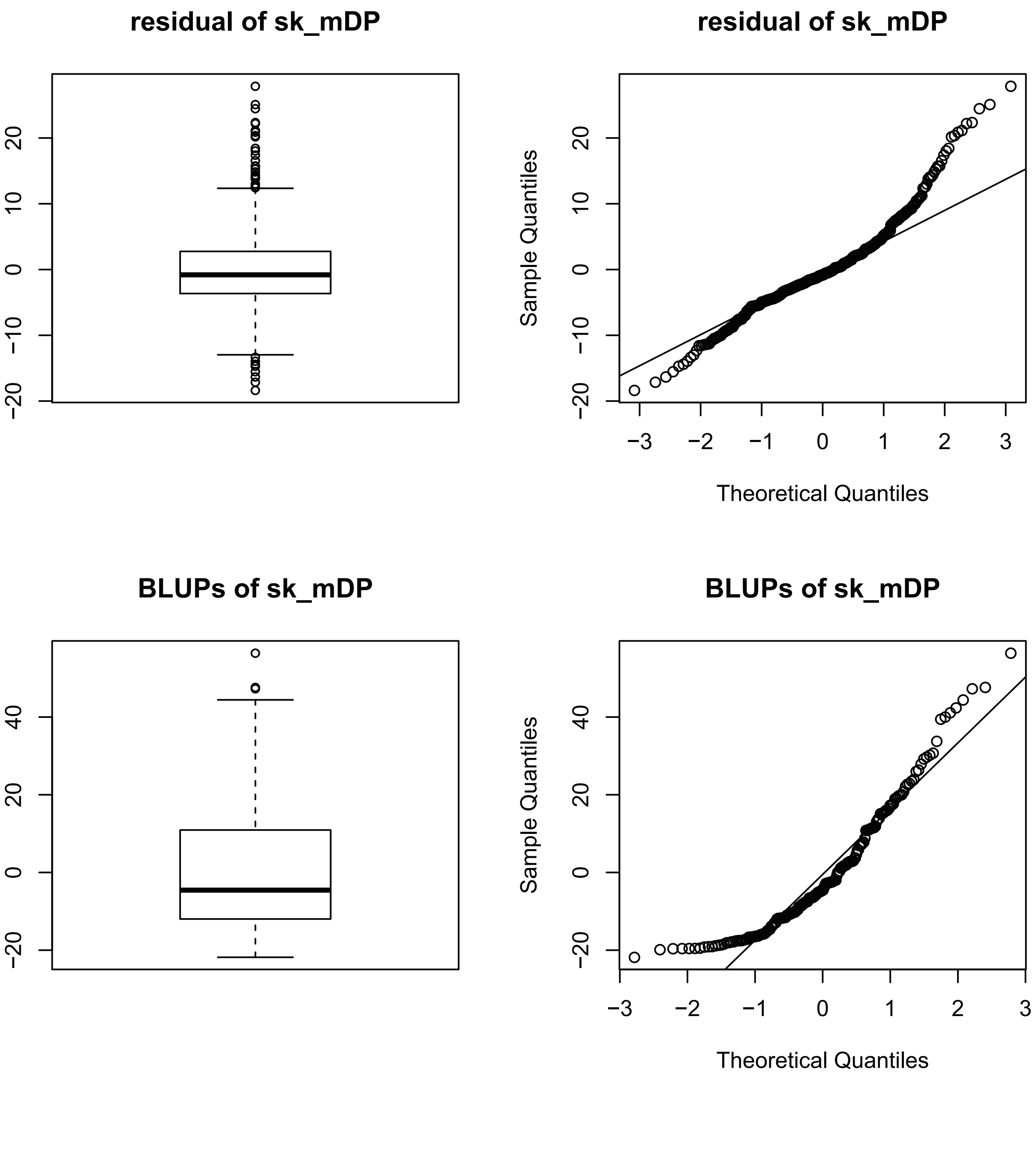
residual of sk_mDP
residual of sk_mDP
Sample Quantiles
Theoretical Quantiles
BLUPs of sk_mDP
BLUPs of sk_mDP
Sample Quantiles
Theoretical Quantiles

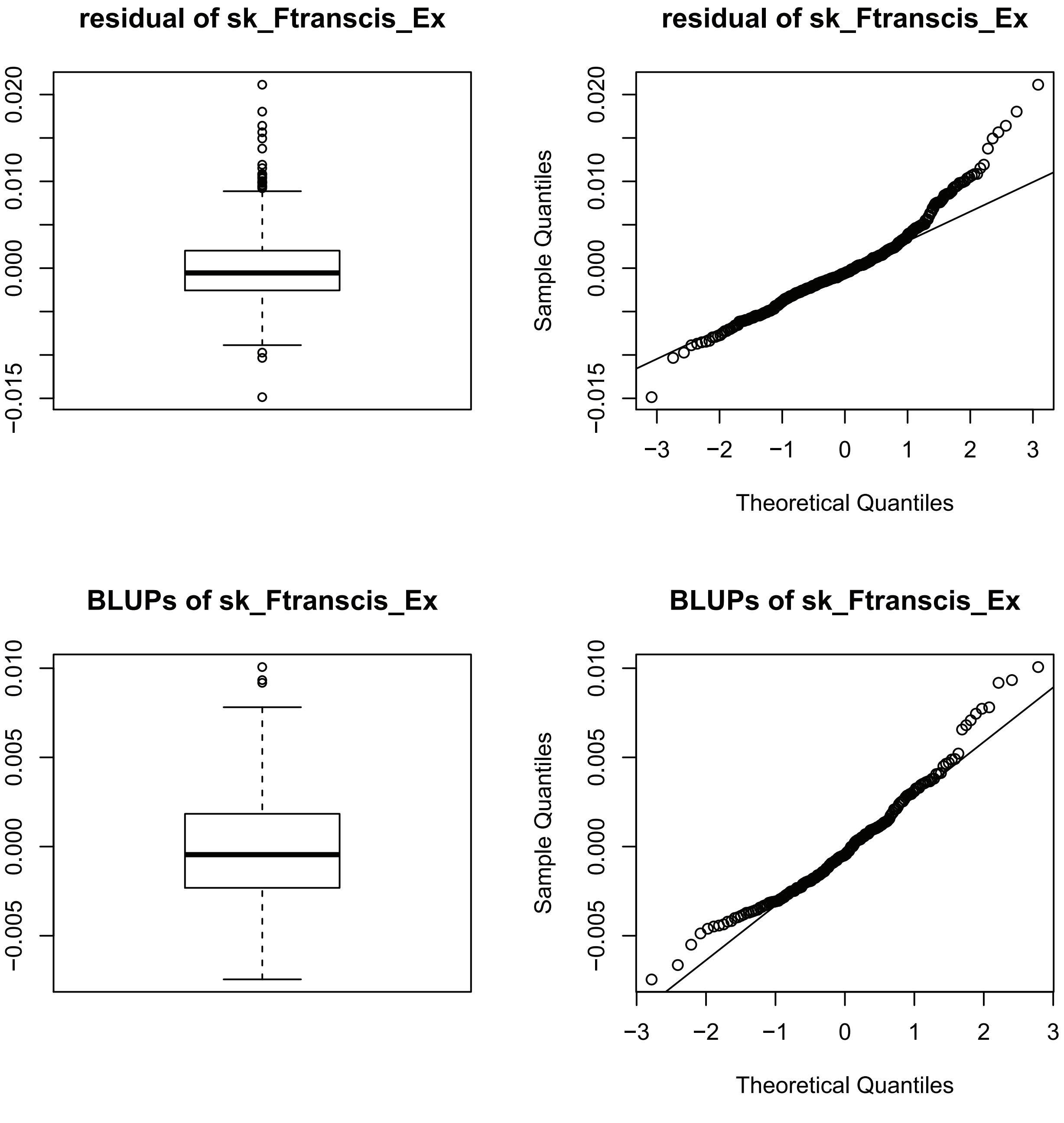
residual of sk_Ftranscis_Ex
residual of sk_Ftranscis_Ex
Sample Quantiles
Theoretical Quantiles
BLUPs of sk_Ftranscis_Ex
BLUPs of sk_Ftranscis_Ex
Sample Quantiles
Theoretical Quantiles

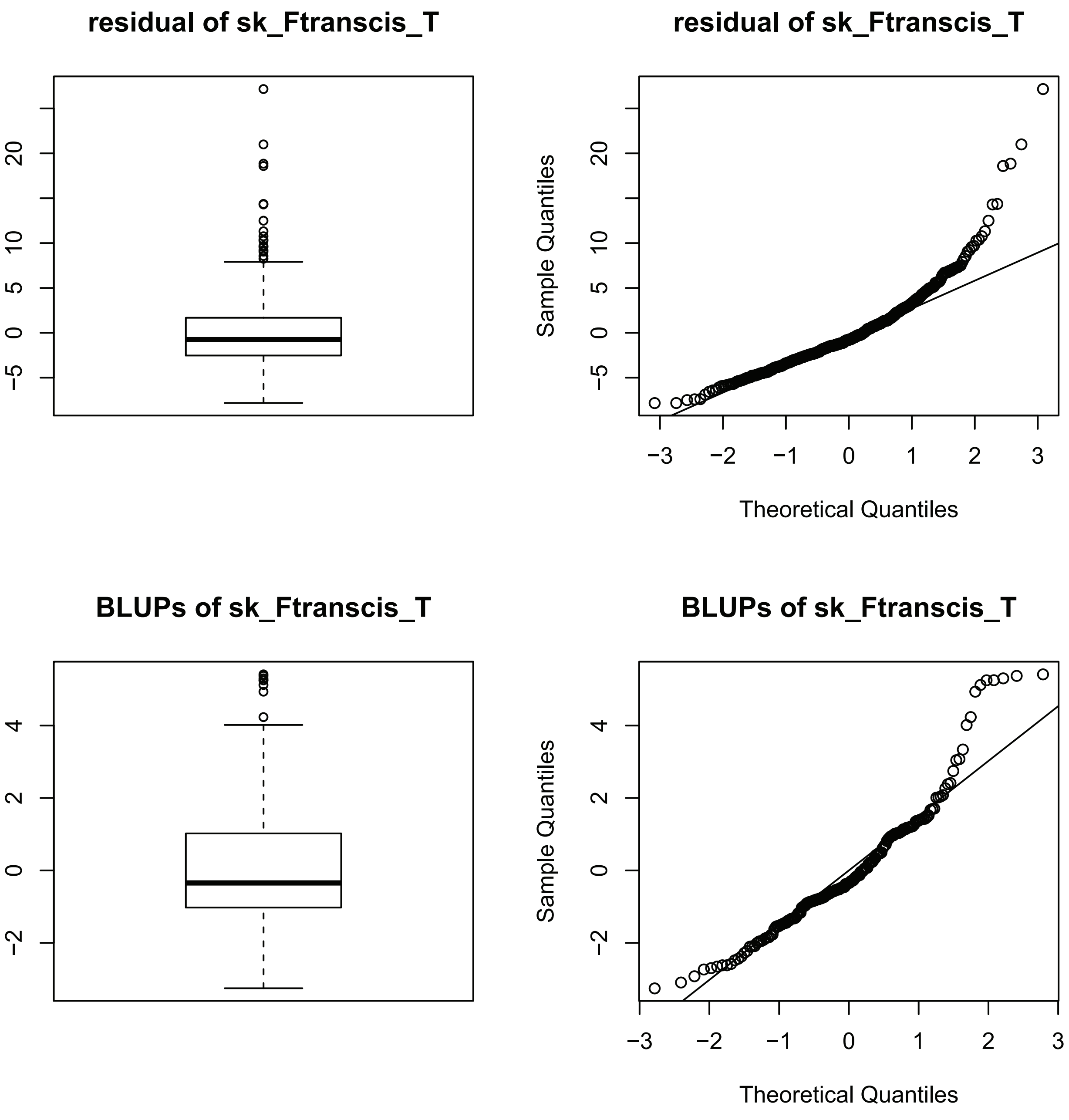
residual of sk_Ftranscis_T
residual of sk_Ftranscis_T
Sample Quantiles
Theoretical Quantiles
BLUPs of sk_Ftranscis_T
BLUPs of sk_Ftranscis_T
Sample Quantiles
Theoretical Quantiles

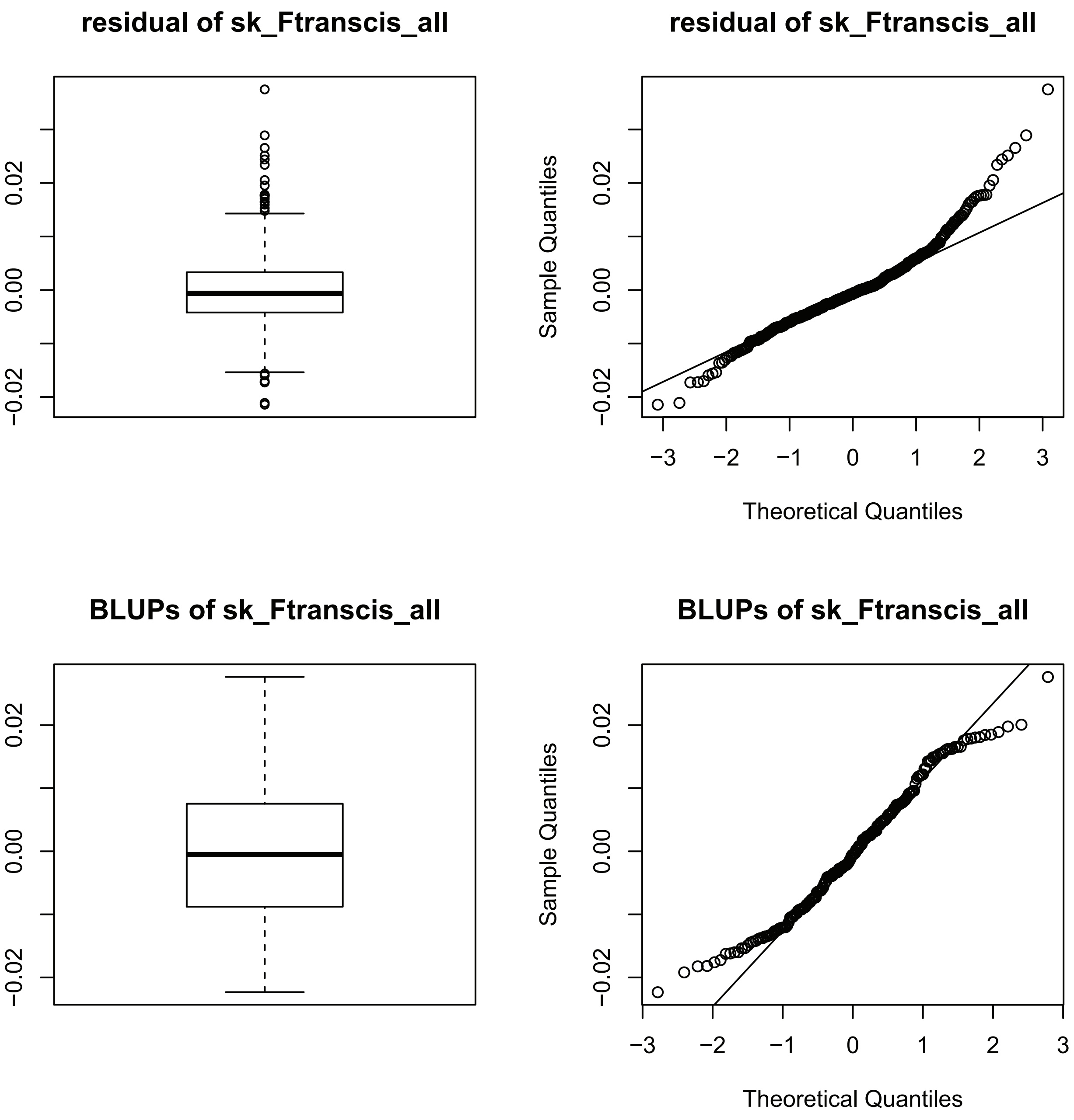
residual of sk_Ftranscis_all
residual of sk_Ftranscis_all
Sample Quantiles
Theoretical Quantiles
BLUPs of sk_Ftranscis_all
BLUPs of sk_Ftranscis_all
Sample Quantiles
Theoretical Quantiles

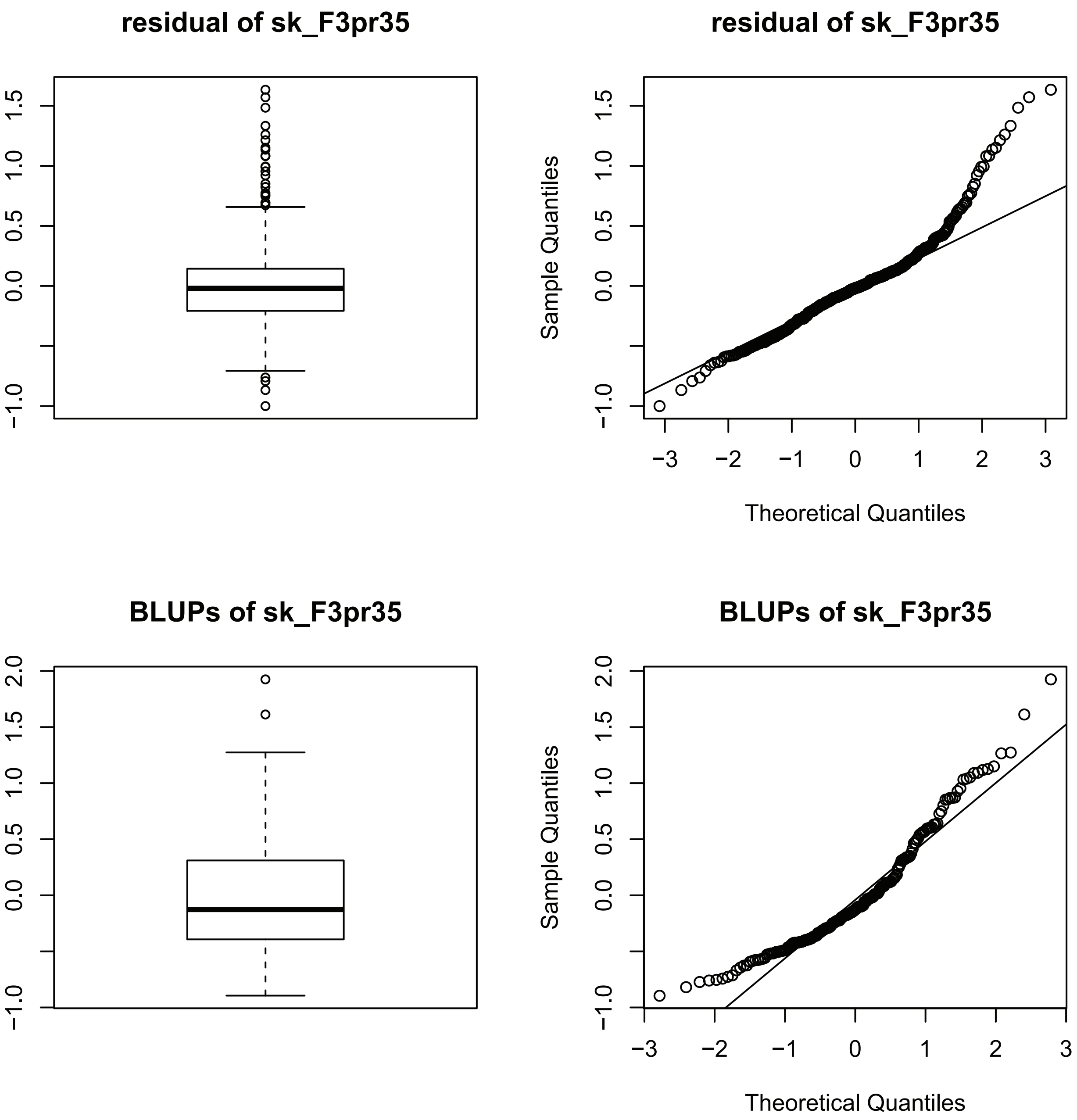
residual of sk_F3pr35
residual of sk_F3pr35
Sample Quantiles
Theoretical Quantiles
BLUPs of sk_F3pr35
BLUPs of sk_F3pr35
Sample Quantiles
Theoretical Quantiles

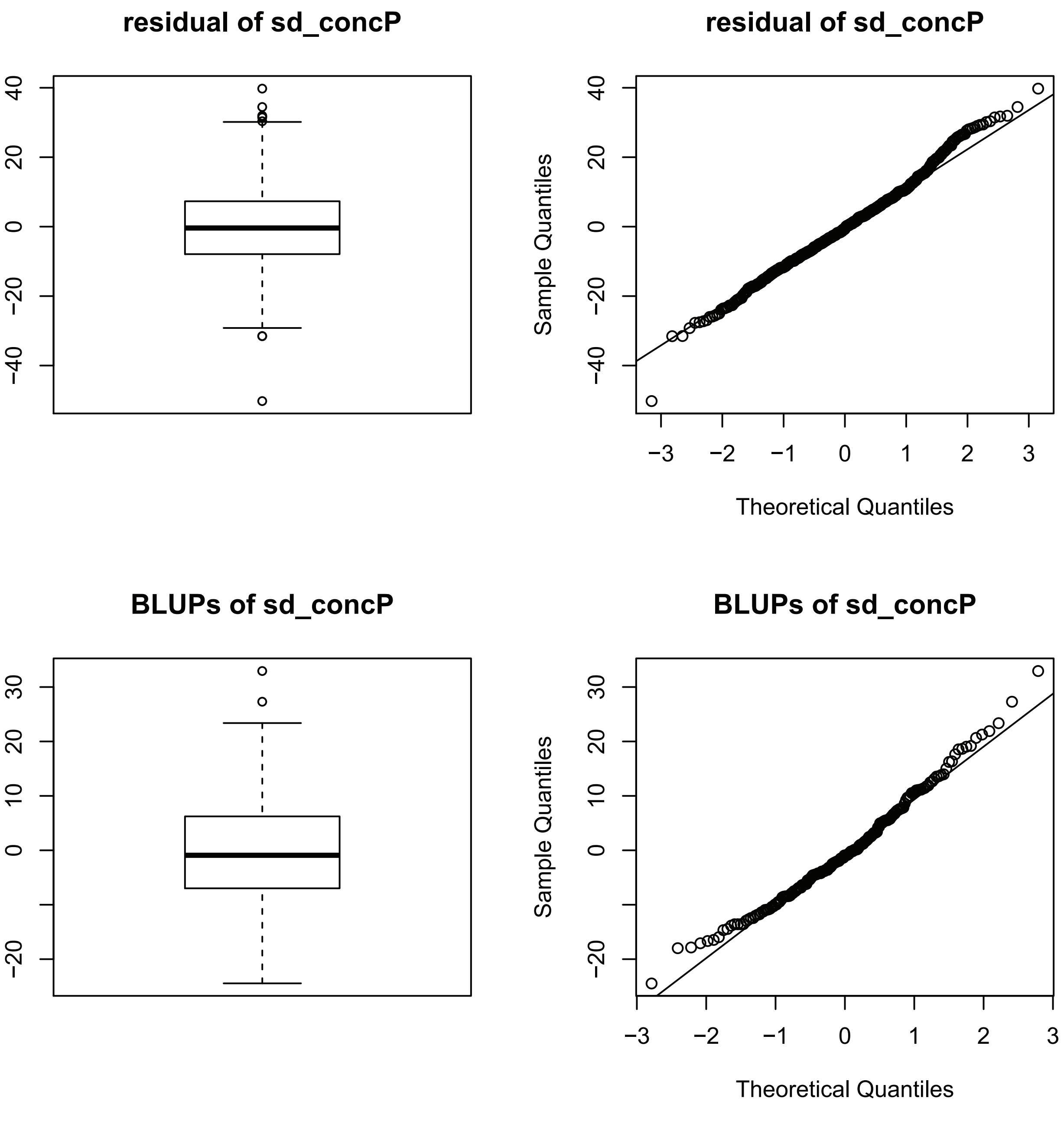
residual of sd_concP
residual of sd_concP
Sample Quantiles
Theoretical Quantiles
BLUPs of sd_concP
BLUPs of sd_concP
Sample Quantiles
Theoretical Quantiles

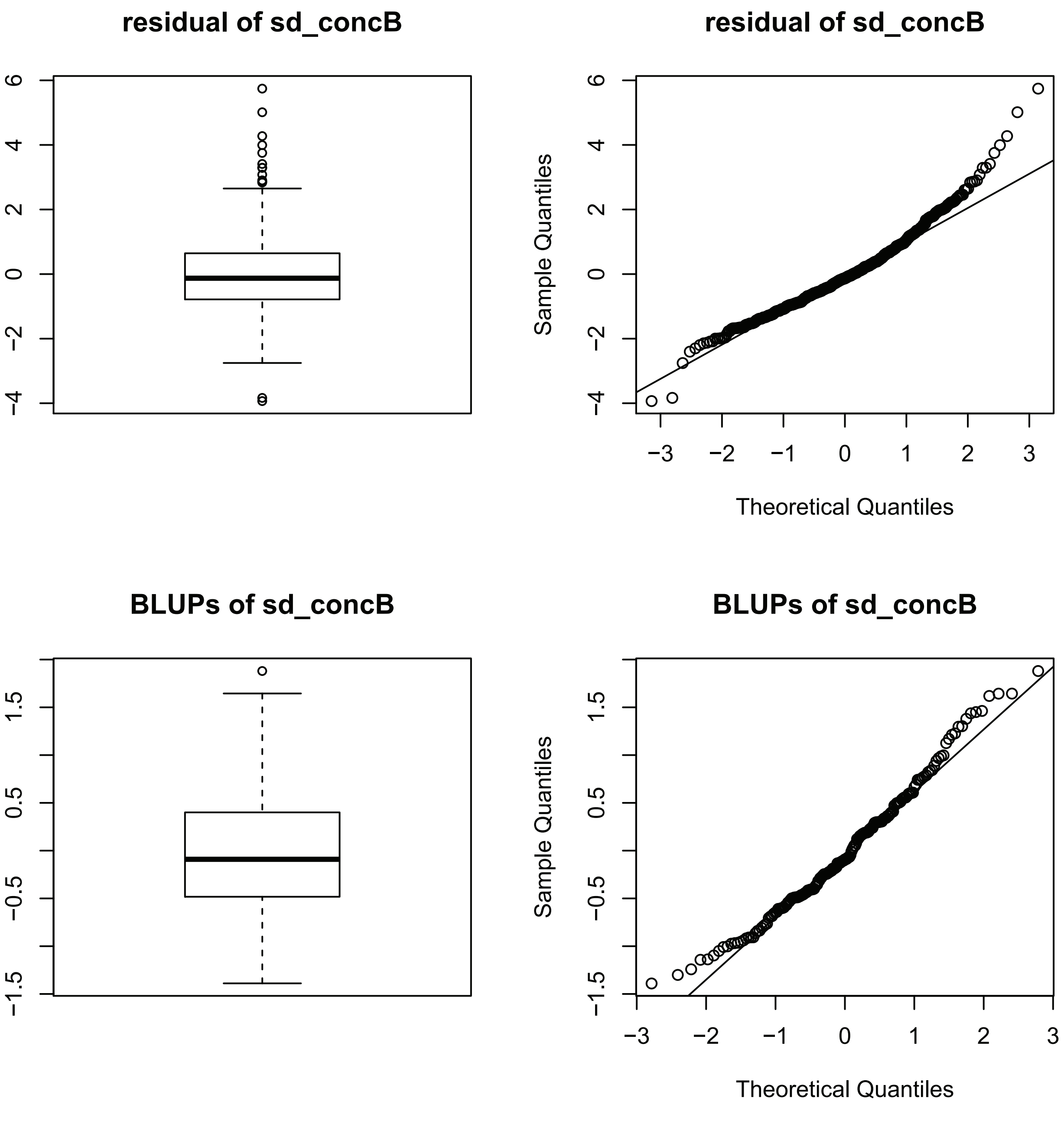
residual of sd_concB
residual of sd_concB
Sample Quantiles
Theoretical Quantiles
BLUPs of sd_concB
BLUPs of sd_concB
Sample Quantiles
Theoretical Quantiles

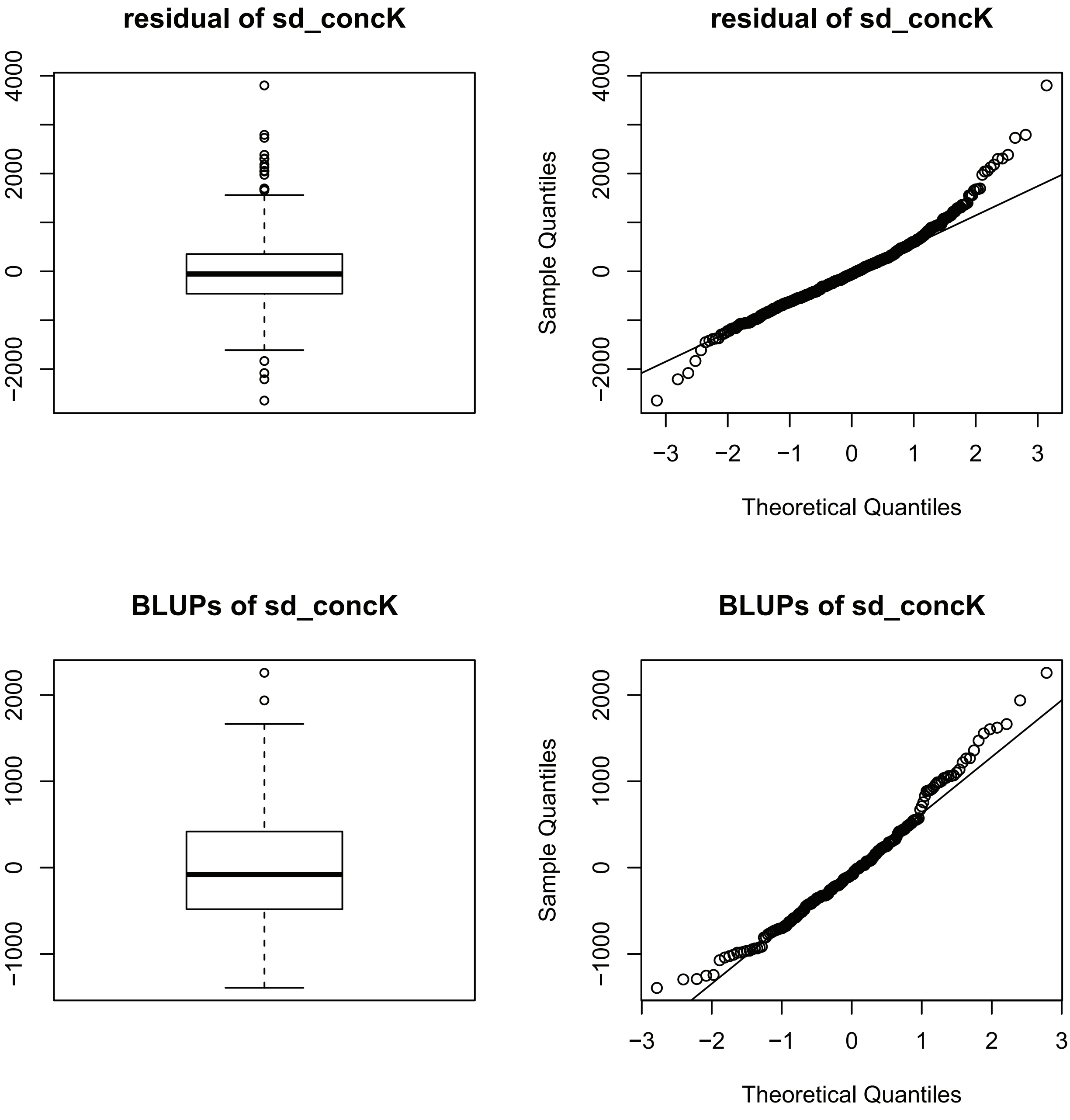
residual of sd_concK
residual of sd_concK
Sample Quantiles
Theoretical Quantiles
BLUPs of sd_concK
BLUPs of sd_concK
Sample Quantiles
Theoretical Quantiles

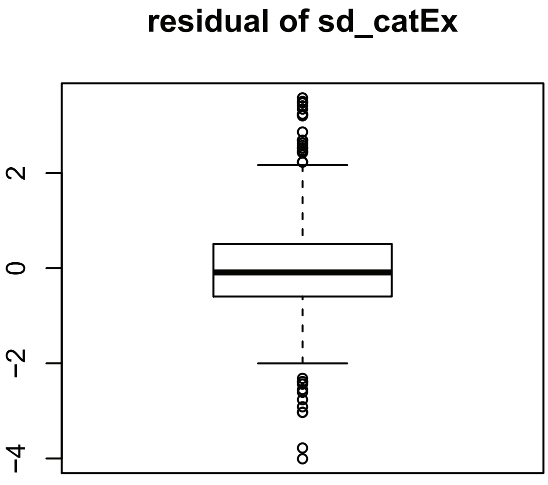
residual of sd_catEx
−4
−2
0
2

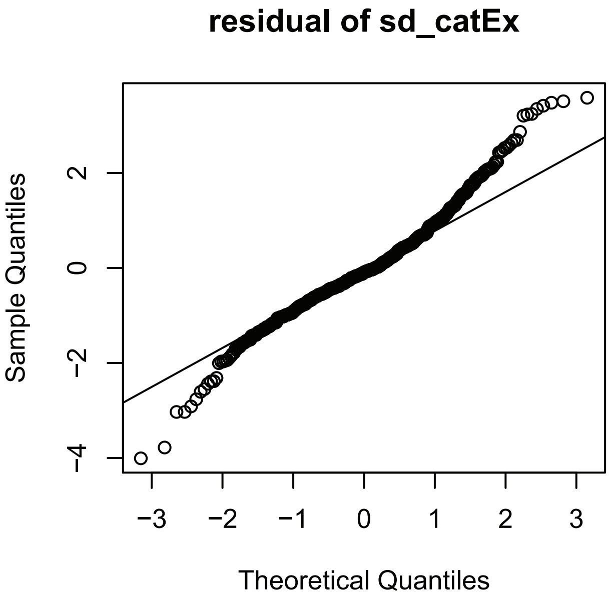
residual of sd_catEx
Sample Quantiles
Theoretical Quantiles
−3 −2 −1 0 1 2 3
−4
−2
0
2

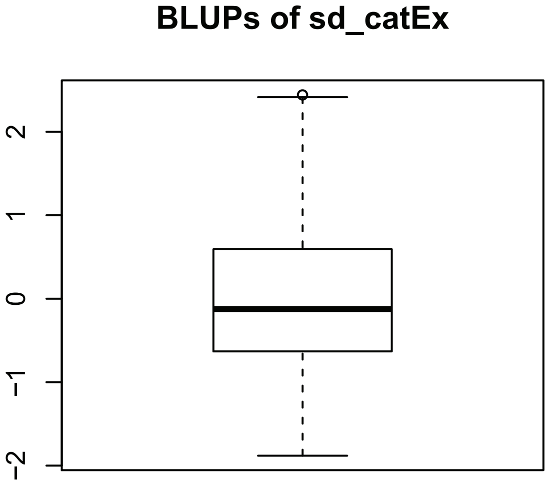
BLUPs of sd_catEx
−2
−1
0
1
2

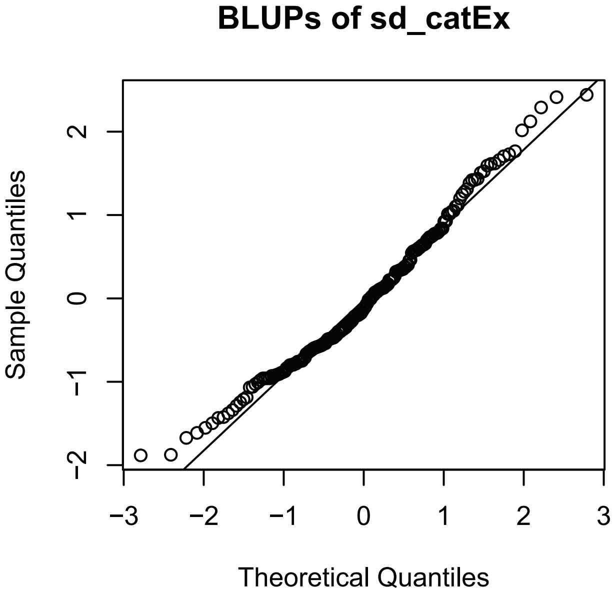
BLUPs of sd_catEx
Sample Quantiles
Theoretical Quantiles
−3 −2 −1 0 1 2 3
−2
−1
0
1
2

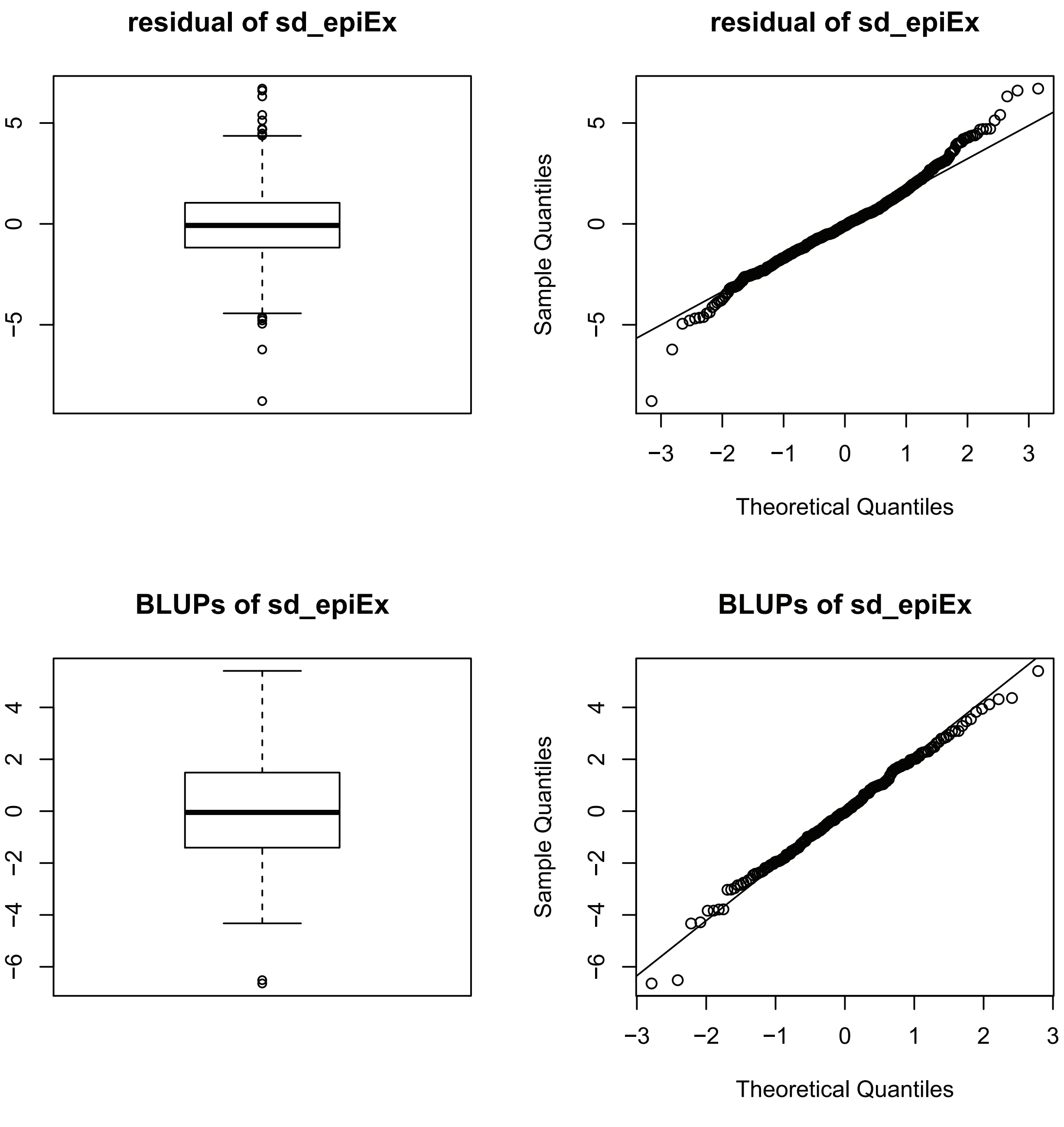
residual of sd_epiEx
residual of sd_epiEx
Sample Quantiles
Theoretical Quantiles
BLUPs of sd_epiEx
BLUPs of sd_epiEx
Sample Quantiles
Theoretical Quantiles

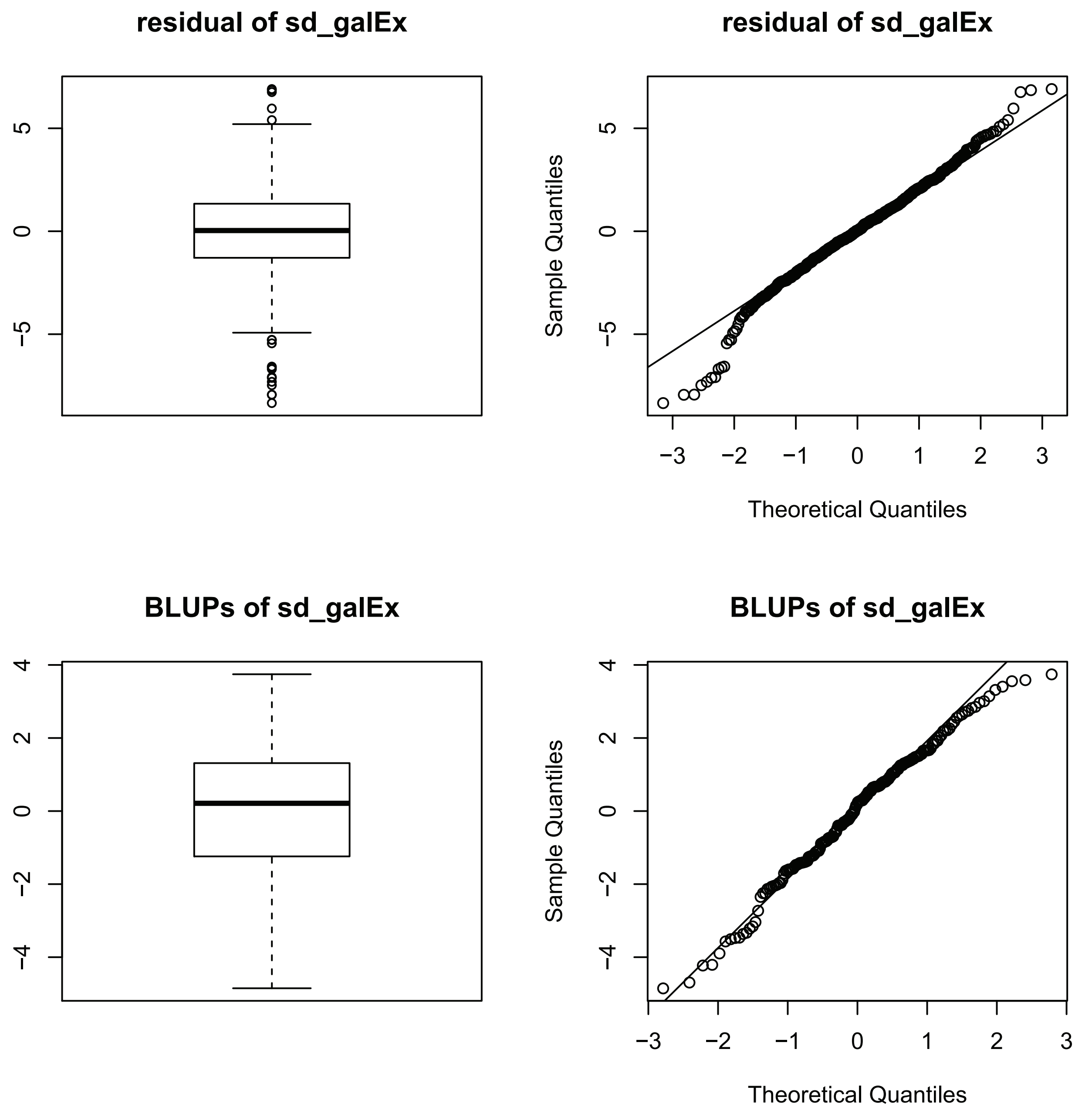
residual of sd_galEx
residual of sd_galEx
Sample Quantiles
Theoretical Quantiles
BLUPs of sd_galEx
BLUPs of sd_galEx
Sample Quantiles
Theoretical Quantiles

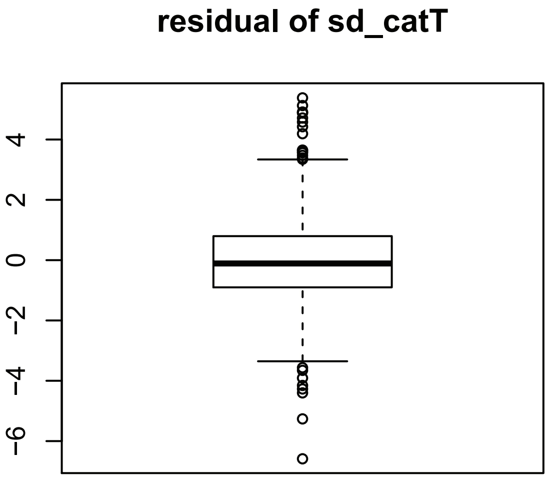
residual of sd_catT
4
2
0
−2
−4
−6

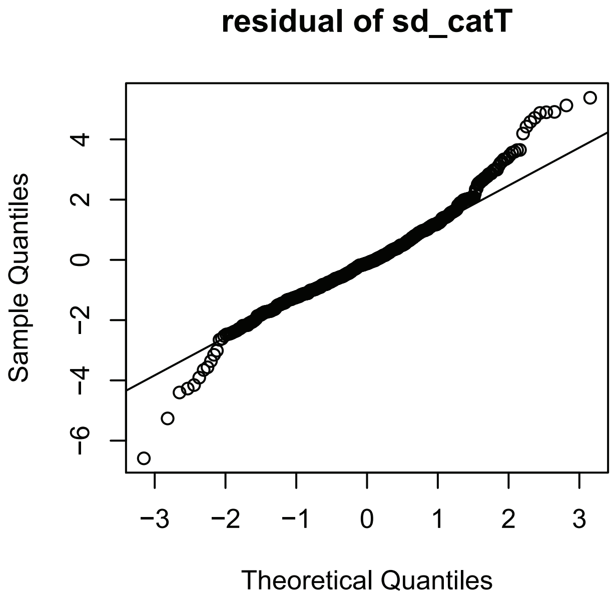
residual of sd_catT
Sample Quantiles
4
2
0
−2
−4
−6
−3
−2
−1
0
1
2
3
Theoretical Quantiles

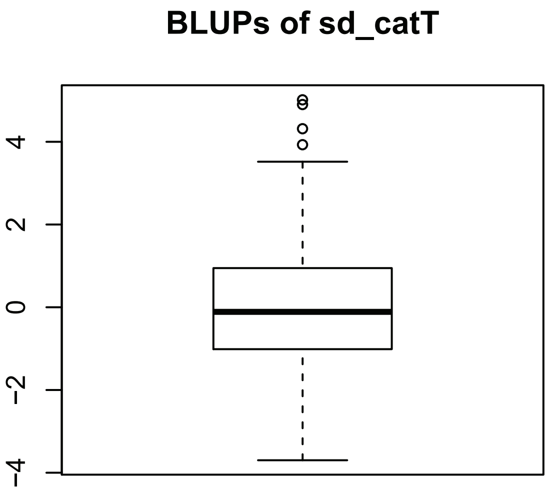
BLUPs of sd_catT
4
2
0
−2
−4

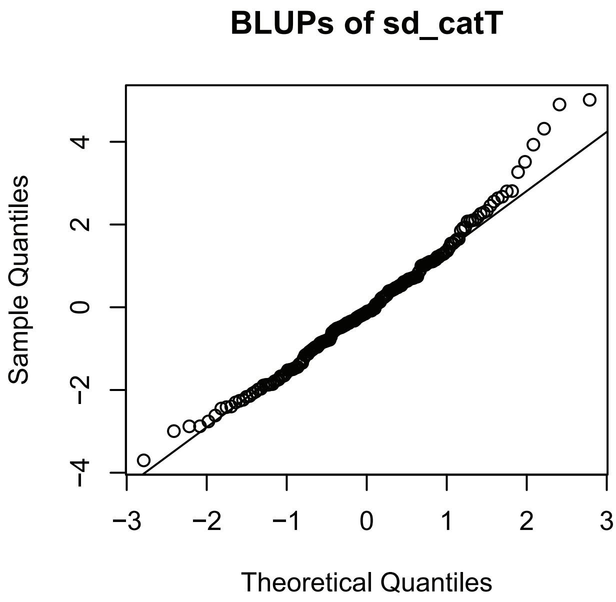
BLUPs of sd_catT
Sample Quantiles
4
2
0
−2
−4
−3
−2
−1
0
1
2
3
Theoretical Quantiles

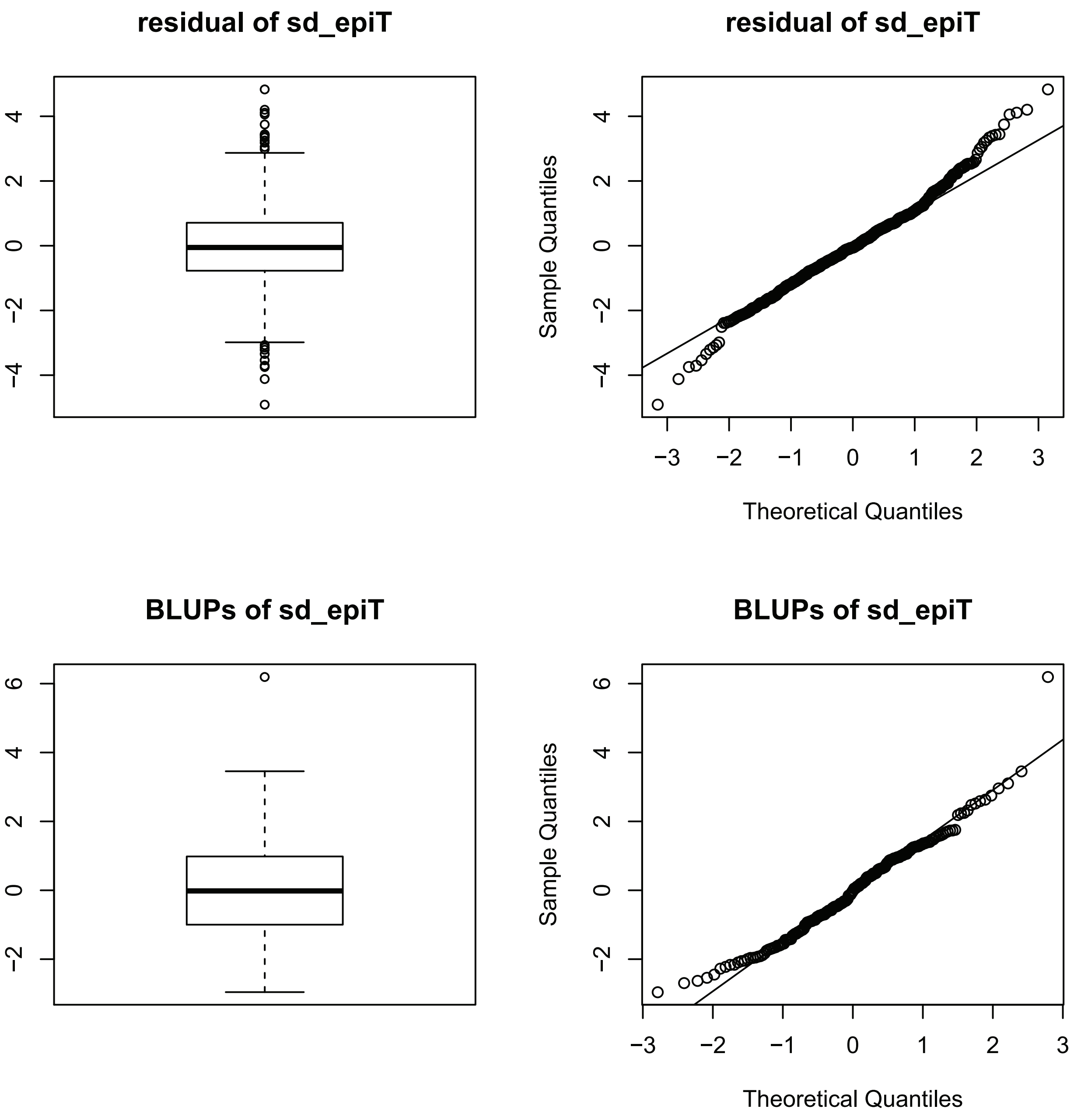
residual of sd_epiT
residual of sd_epiT
Sample Quantiles
Theoretical Quantiles
BLUPs of sd_epiT
BLUPs of sd_epiT
Sample Quantiles
Theoretical Quantiles

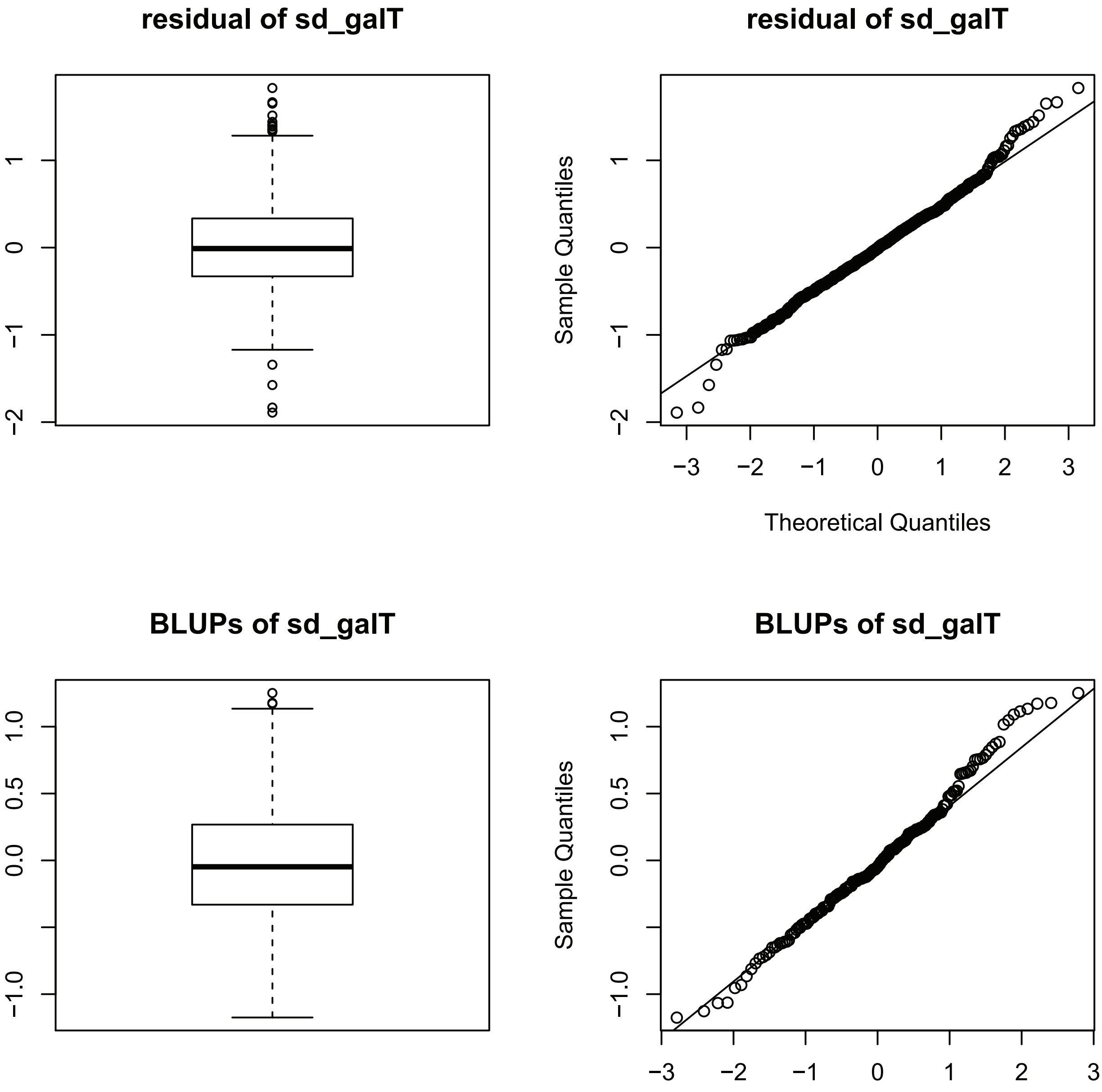

residual of sd_galT
residual of sd_galT
Sample Quantiles
Theoretical Quantiles
BLUPs of sd_galT
BLUPs of sd_galT
Sample Quantiles
Theoretical Quantiles

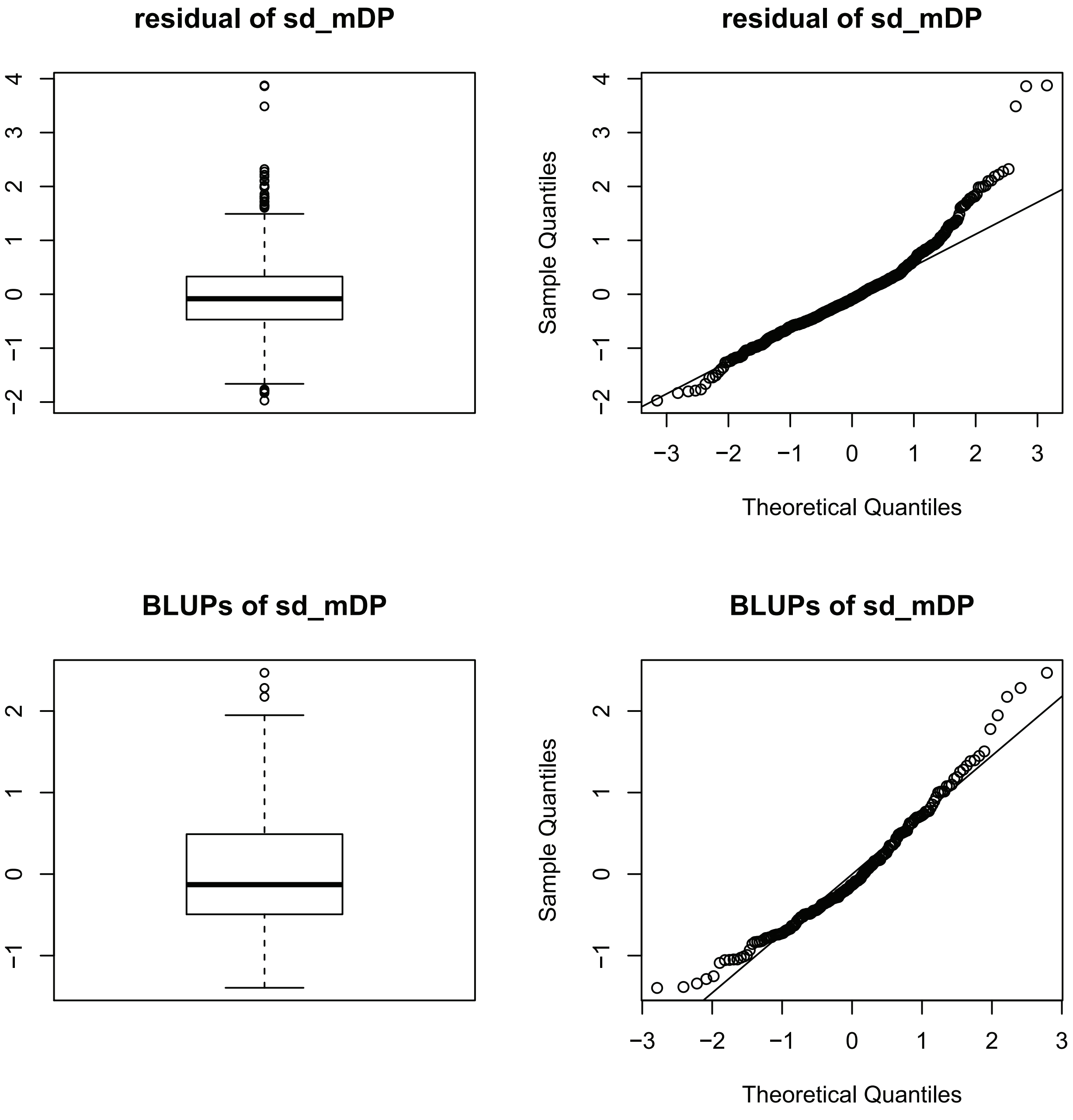
residual of sd_mDP
residual of sd_mDP
Sample Quantiles
Theoretical Quantiles
BLUPs of sd_mDP
BLUPs of sd_mDP
Sample Quantiles
Theoretical Quantiles

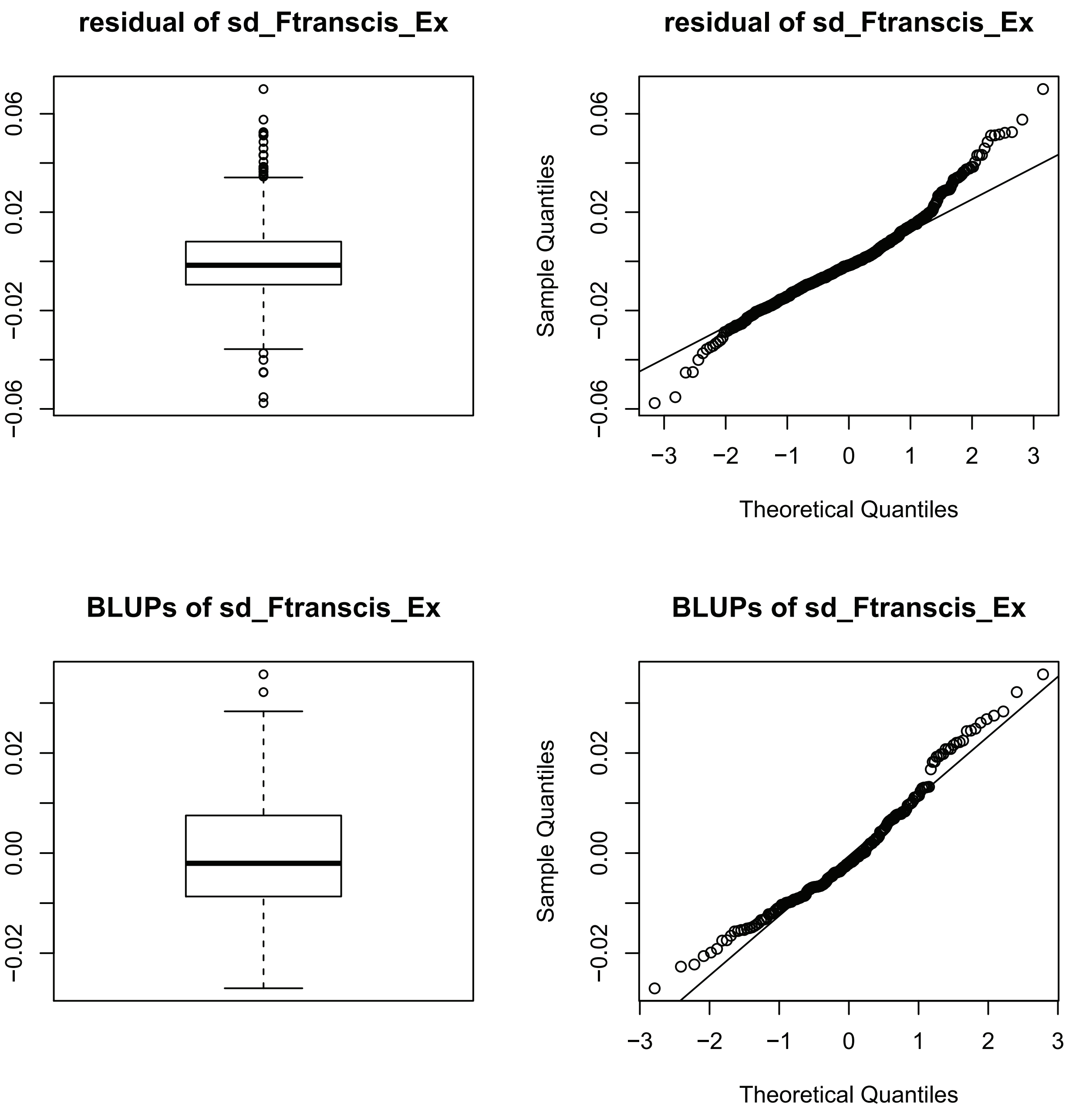
residual of sd_Ftranscis_Ex
0.06
0.02
−0.02
−0.06
residual of sd_Ftranscis_Ex
Sample Quantiles
Theoretical Quantiles
−3
−2
−1
0
1
2
3
BLUPs of sd_Ftranscis_Ex
0.02
0.00
−0.02
BLUPs of sd_Ftranscis_Ex
Sample Quantiles
Theoretical Quantiles

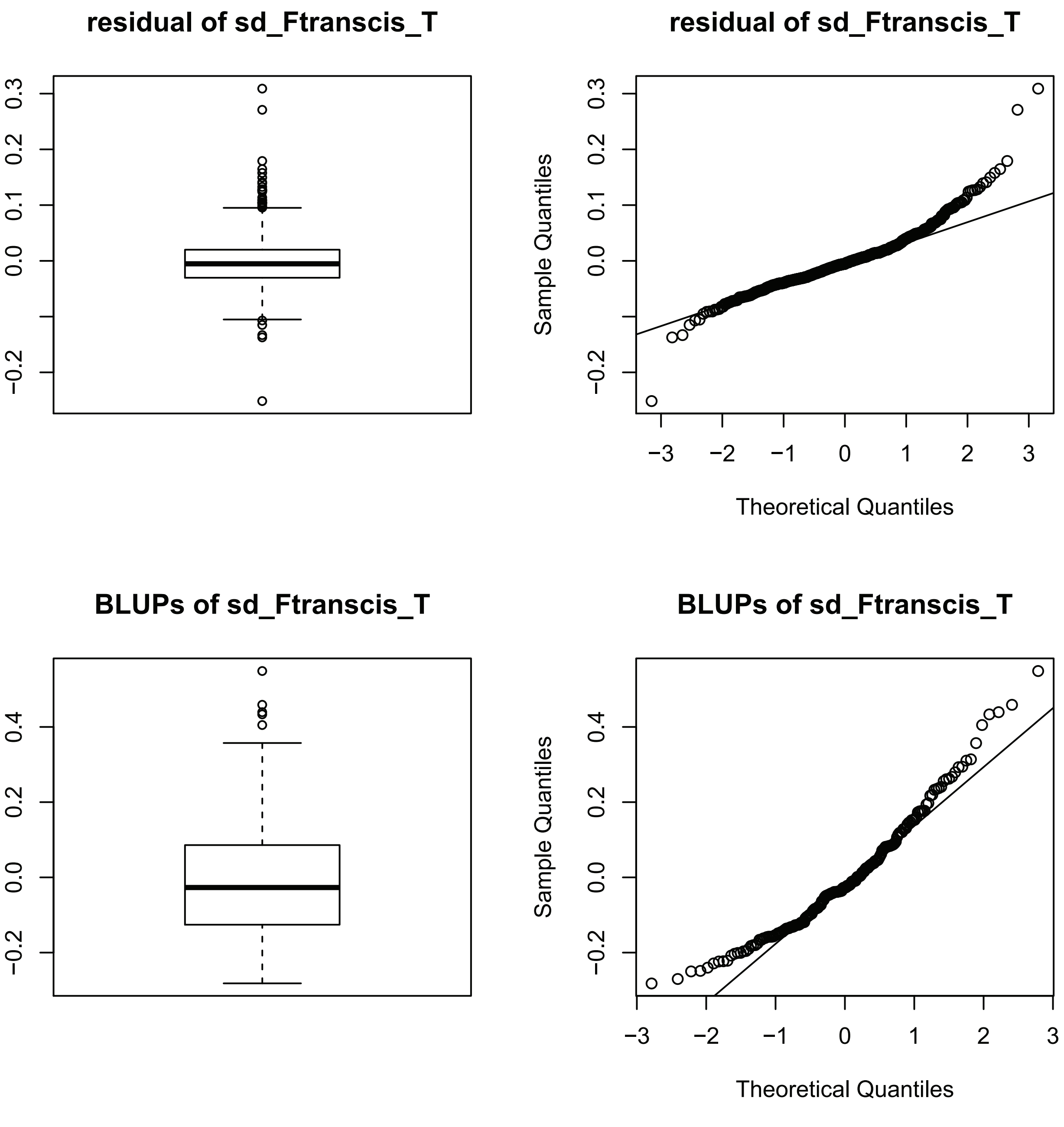
residual of sd_Ftranscis_T
residual of sd_Ftranscis_T
Sample Quantiles
Theoretical Quantiles
BLUPs of sd_Ftranscis_T
BLUPs of sd_Ftranscis_T
Sample Quantiles
Theoretical Quantiles

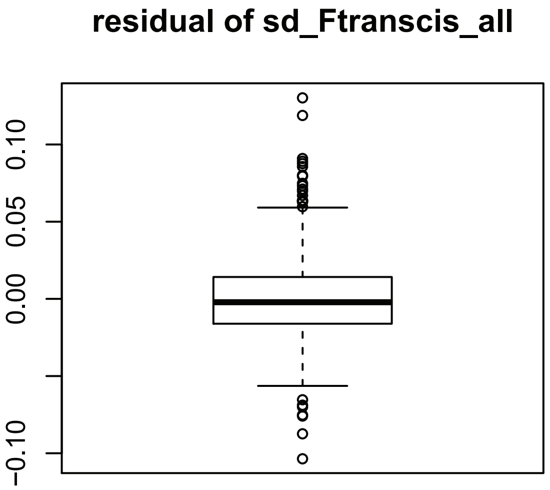
residual of sd_Ftranscis_all
−0.10
0.00
0.05
0.10

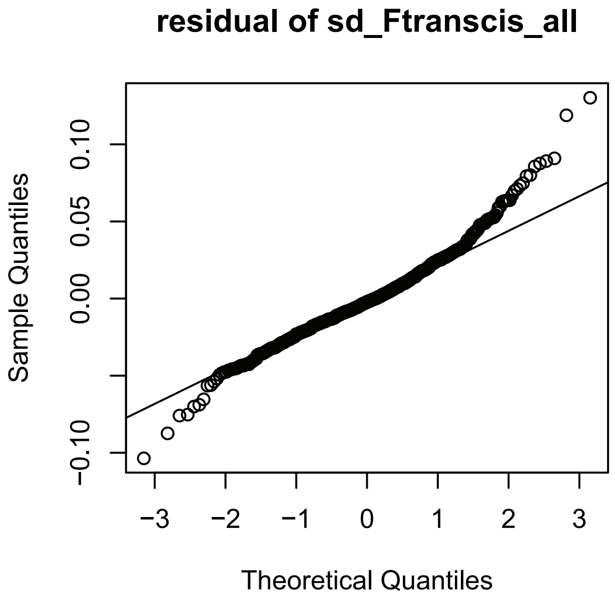
residual of sd_Ftranscis_all
Sample Quantiles
−0.10
0.00
0.05
0.10
−3
−2
−1
0
1
2
3
Theoretical Quantiles

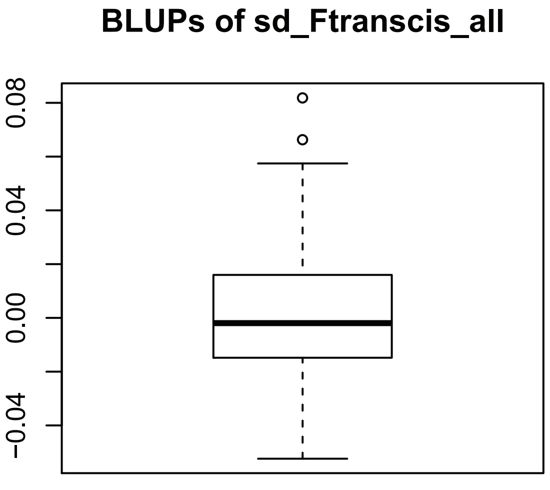
BLUPs of sd_Ftranscis_all
−0.04
0.00
0.04
0.08

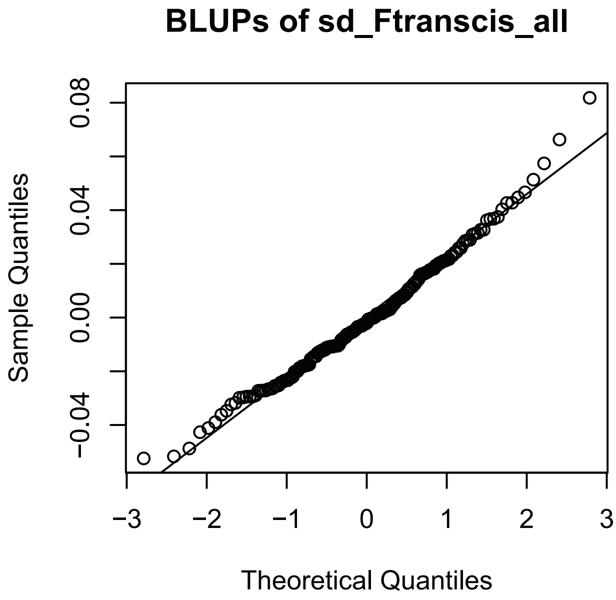
BLUPs of sd_Ftranscis_all
Sample Quantiles
−0.04
0.00
0.04
0.08
−3
−2
−1
0
1
2
3
Theoretical Quantiles
