# Supplementary material for: Dissecting genetic architecture of grape proanthocyanidin composition through quantitative trait locus mapping
Source: BMC Plant Biol. 2012 Feb 27;12:30. doi: 10.1186/1471-2229-12-30 (PMC3312867; doi:10.1186/1471-2229-12-30)
Supplement: Additional file 3 — Phenotypic data analysis and best fitted models for variance component estimation. Analysis method and effects included in the best fitted model. [file 1471-2229-12-30-S3.PDF]

Additional file 3: Phenotypic data analysis and best fitted model for variance component estimation

For the S×G population, 7-16% (2005), 11-17% (2006) and 25-28% (2007) phenotypic data were missing, mainly due to berry rot at harvest. One to four aberrant values were discarded according to variables. For the CC population, 7-16% in 2005 and 19-24% in 2006 phenotypic data were missing, also mainly due to berry rot and inflorescence absence, with one to three data per variable discarded due to aberrant values. All statistical analyses were performed with R software (R-Development-Core-Team 2008). In order to identify the best-fit model for each PA variable, different mixed models were fitted with lme4 package (Bates *et al.* 2008). Model comparison was performed from the simplest model, based only on general mean and random genotypic effect, to the most complete one, based on general mean, random genotypic effect, fixed year effect and random genotype × year effect. For data sets where 2-block data were available, fixed block effect was included and the random genotype × year effect was substituted by a random genotype × block effect nested in a given year. The Bayesian information criterion (BIC) was used for model selection. For all PA variables both in skin and seeds, the best fitted model included both the genotypic and year effects except for concB in skin and mDP in seeds, for which the year effect was not significant (see the table below). We verified ~~the~~ mixed model fitting validity through quantile-quantile plot comparing residual and random effect predictors versus a theoretical normal distribution. No phenotypes required data transformation.

Based on the best fitted model, we estimated broad-sense heritability (H^2) and extracted genotypic best linear unbiased predictors (BLUP) for each PA variable. H^2 was estimated as the proportion of phenotypic variance explained by genetic variance. The variance components were estimated by restricted maximum likelihood method as it is considered the most suitable procedure to estimate variance components for unbalanced data (Dieters *et al.* 1995).

Table S : Best fitted model for variance component estimation. Effects included in the best fitted model are indicated by an asterisk.

(A) S×G population

Tissue	Trait	Genotype	year	block	Genotype×year	(Genotype×block)|year
skin	concP	*				
	concB	*	*			
	concK	*	*	*		
	catEx	*	*			
	epiEx	*	*			
	galEx	*	*			
	egcEx	*	*			
	catT	*	*			
	epiT	*	*			
	mDP	*	*			
	F3pr35	*	*			
	Ftranscis_Ex	*	*		*	
	Ftranscis_T	*	*			
	Ftranscis_all	*	*		*	
seed	concP	*	*			
	concB	*	*		*	
	concK	*	*	*		
	catEx	*	*			
	epiEx	*	*			
	galEx	*	*			
	catT	*	*			
	epiT	*	*			
	galT	*	*			
	mDP	*				
	Ftranscis_Ex	*	*			
	Ftranscis_T	*	*	*		*
	Ftranscis_all	*	*			

(B) CC

Tissue	Trait	Genotype	year
skin	concP	*	*
	concB	*	*
	concK	*	*
	catEx	*	*
	epiEx	*	*
	galEx	*	*
	egcEx	*	
	catT	*	*
	epiT	*	
	mDP	*	*
	Ftranscis_Ex	*	*
	Ftranscis_T	*	*
	Ftranscis_all	*	
	F3pr35	*	

References

Bates, D., Maechler, M. and Dai, B. (2008) lme4: Linear mixed-effects models using S4 classes.

Dieters, M.J., White, T.L., Littell, R.C. and Hodge, G.R. (1995) Application of approximate variances of variance components and their ratios in genetic tests. *TAG Theoretical and Applied Genetics*, **91**, 15-24.

R-Development-Core-Team (2008) R: A Language and Environment for Statistical Computing.
