# Supplementary material for: Dissecting genetic architecture of grape proanthocyanidin composition through quantitative trait locus mapping
Source: BMC Plant Biol. 2012 Feb 27;12:30. doi: 10.1186/1471-2229-12-30 (PMC3312867; doi:10.1186/1471-2229-12-30)
Supplement: Additional file 4 — Effect of minor genotypic frequency and non-normalty of observed phenotype on the association test. Two sections are in this file. 1. Test for the enrichment of low frequency polymorphisms among associated markers. 2. Test for the effect of the non-normalty of the trait in the association tests. [file 1471-2229-12-30-S4.PDF]

1. **Test for the enrichment of low frequency polymorphisms among associated markers**

We tested whether marker frequency had an effect on the results of the association study. We first calculated the minor genotypic frequency since TASSEL package uses genotypic data to perform the association test. We then tested the effect of genotypic frequency on the corresponding *P*-value in the association tests using general linear models and represented the *P*-value of association results by using minor genotypic frequency with bins of 0.1 (Figure S1, see below). For twenty-three out of twenty-seven PA variables assessed in this work, significance of the association tests was not dependent on marker frequency. Four skin variables, concK, galEx, epiT and mDP, showed dependence between *P*-value of association tests and marker frequency (*P*-value of regression coefficient <0.01). For concK, smaller genotypic frequencies tended to have small *P*-value in association tests (Figure S1) and this tendency was observed for markers whose frequency was between 0.1 to 0.5 while regression fitted less well the frequency bin of 0-0.1. For galEx, epiT and mDP, small *P*-value of association results tended to be identified for markers with equilibrium frequency. In summary, this test supported that there was no enrichment of low frequency polymorphisms among associated markers.

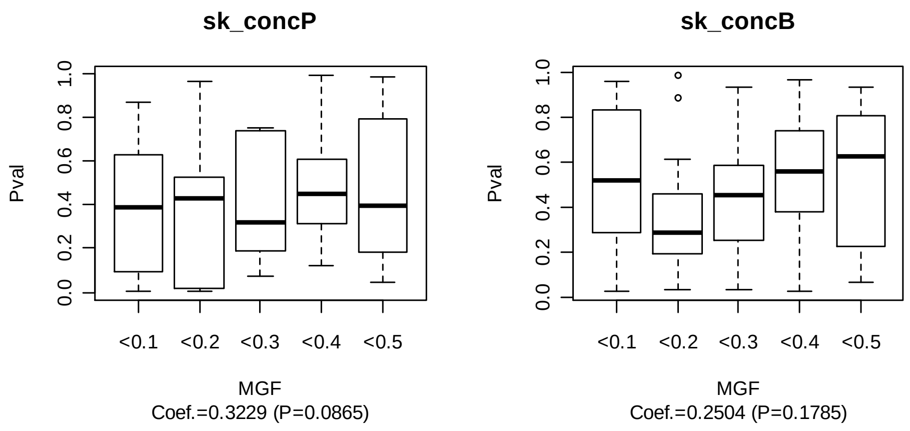
sk_concP
Pval
MGF
Coef.=0.3229 (P=0.0865)
sk_concB
Pval
MGF
Coef.=0.2504 (P=0.1785)
sk_concK
Pval
MGF
Coef.=0.5164 (P=0.0072)
sk_catEx
Pval
MGF
Coef.=0.0545 (P=0.7659)
<0.1 <0.2 <0.3 <0.4 <0.5

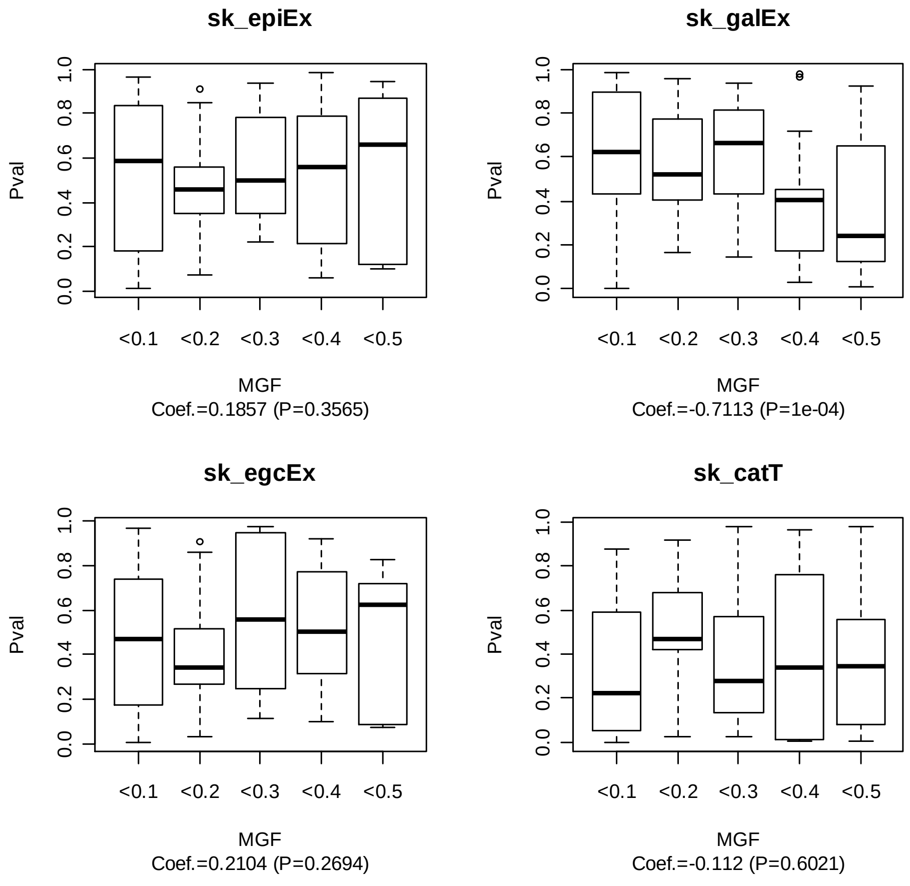
sk_epiEx
Pval
MGF
Coef.=0.1857 (P=0.3565)
sk_galEx
Pval
MGF
Coef.=-0.7113 (P=1e-04)
sk_egcEx
Pval
MGF
Coef.=0.2104 (P=0.2694)
sk_catT
Pval
MGF
Coef.=-0.112 (P=0.6021)
<0.1 <0.2 <0.3 <0.4 <0.5

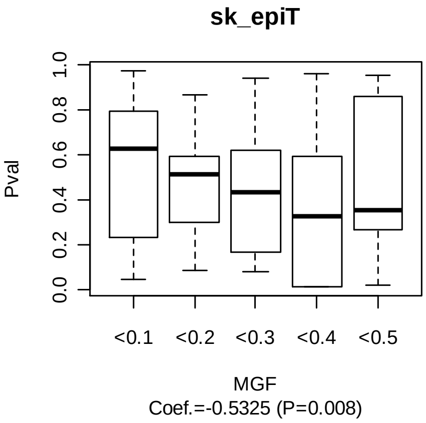
sk_epiT
Pval
0.0 0.2 0.4 0.6 0.8 1.0
<0.1 <0.2 <0.3 <0.4 <0.5
MGF
Coef.=-0.5325 (P=0.008)

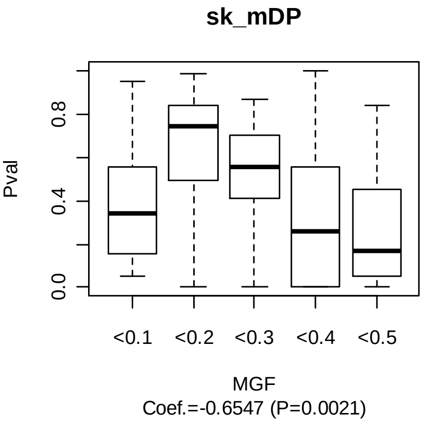
sk_mDP
Pval
0.0 0.4 0.8
<0.1 <0.2 <0.3 <0.4 <0.5
MGF
Coef.=-0.6547 (P=0.0021)

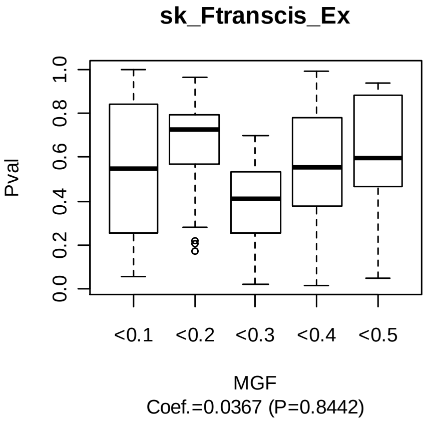
sk_Ftranscis_Ex
Pval
0.0 0.2 0.4 0.6 0.8 1.0
<0.1 <0.2 <0.3 <0.4 <0.5
MGF
Coef.=0.0367 (P=0.8442)

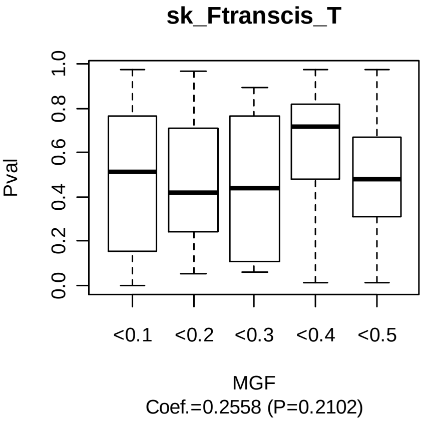
sk_Ftranscis_T
Pval
0.0 0.2 0.4 0.6 0.8 1.0
<0.1 <0.2 <0.3 <0.4 <0.5
MGF
Coef.=0.2558 (P=0.2102)

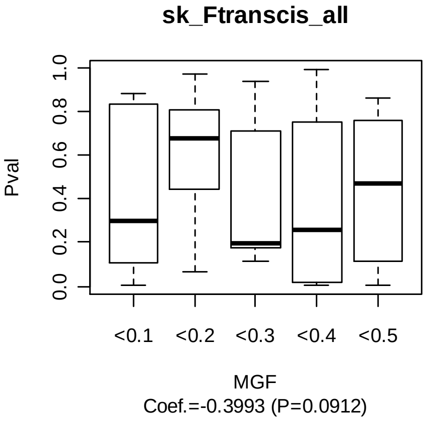
sk_Ftranscis_all
Pval
0.0 0.2 0.4 0.6 0.8 1.0
<0.1 <0.2 <0.3 <0.4 <0.5
MGF
Coef.=-0.3993 (P=0.0912)

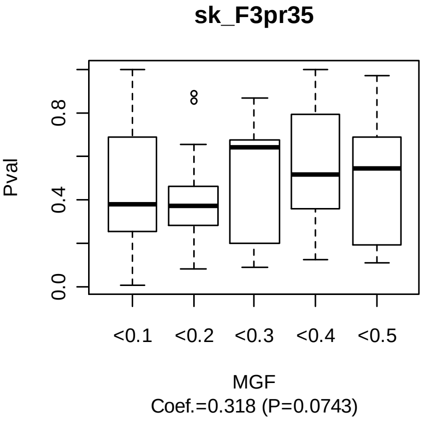
sk_F3pr35
Pval
0.0 0.4 0.8
<0.1 <0.2 <0.3 <0.4 <0.5
MGF
Coef.=0.318 (P=0.0743)

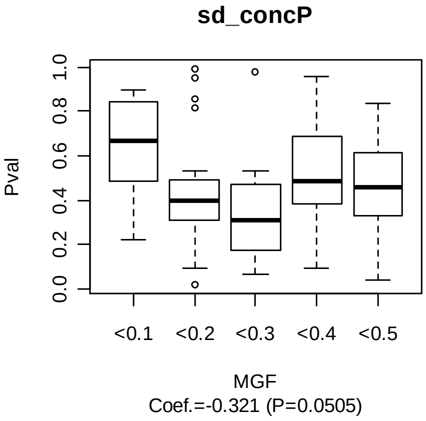
sd_concP
Pval
0.0 0.2 0.4 0.6 0.8 1.0
<0.1 <0.2 <0.3 <0.4 <0.5
MGF
Coef.=-0.321 (P=0.0505)

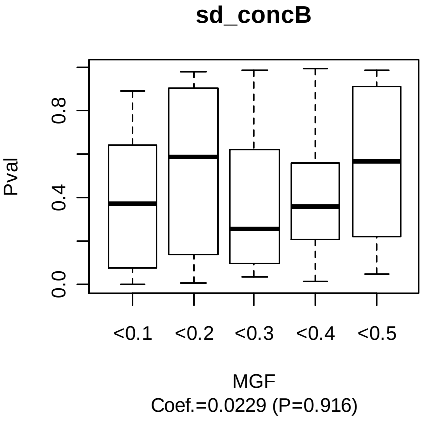
sd_concB
Pval
0.0 0.4 0.8
<0.1 <0.2 <0.3 <0.4 <0.5
MGF
Coef.=0.0229 (P=0.916)

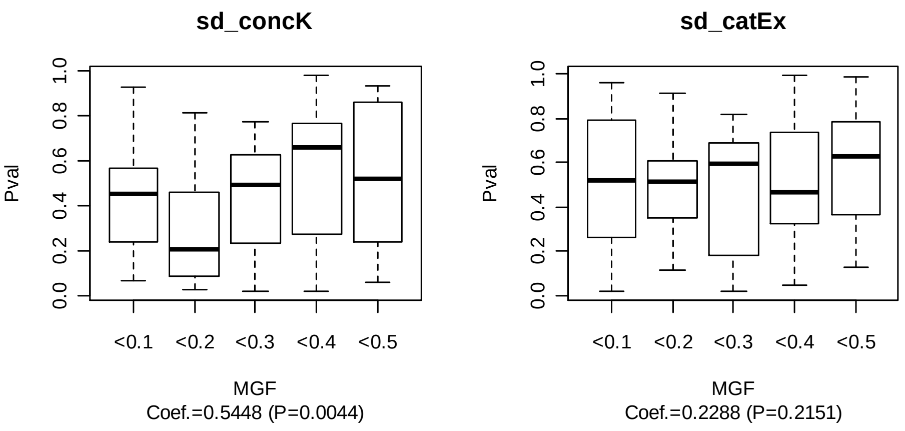
sd_concK
Pval
0.0 0.2 0.4 0.6 0.8 1.0
<0.1 <0.2 <0.3 <0.4 <0.5
MGF
Coef.=0.5448 (P=0.0044)
sd_catEx
Pval
0.0 0.2 0.4 0.6 0.8 1.0
<0.1 <0.2 <0.3 <0.4 <0.5
MGF
Coef.=0.2288 (P=0.2151)
sd_epiEx
Pval
0.0 0.2 0.4 0.6 0.8 1.0
<0.1 <0.2 <0.3 <0.4 <0.5
MGF
Coef.=0.2412 (P=0.186)
sd_galEx
Pval
0.0 0.2 0.4 0.6 0.8 1.0
<0.1 <0.2 <0.3 <0.4 <0.5
MGF
Coef.=-0.0958 (P=0.6089)

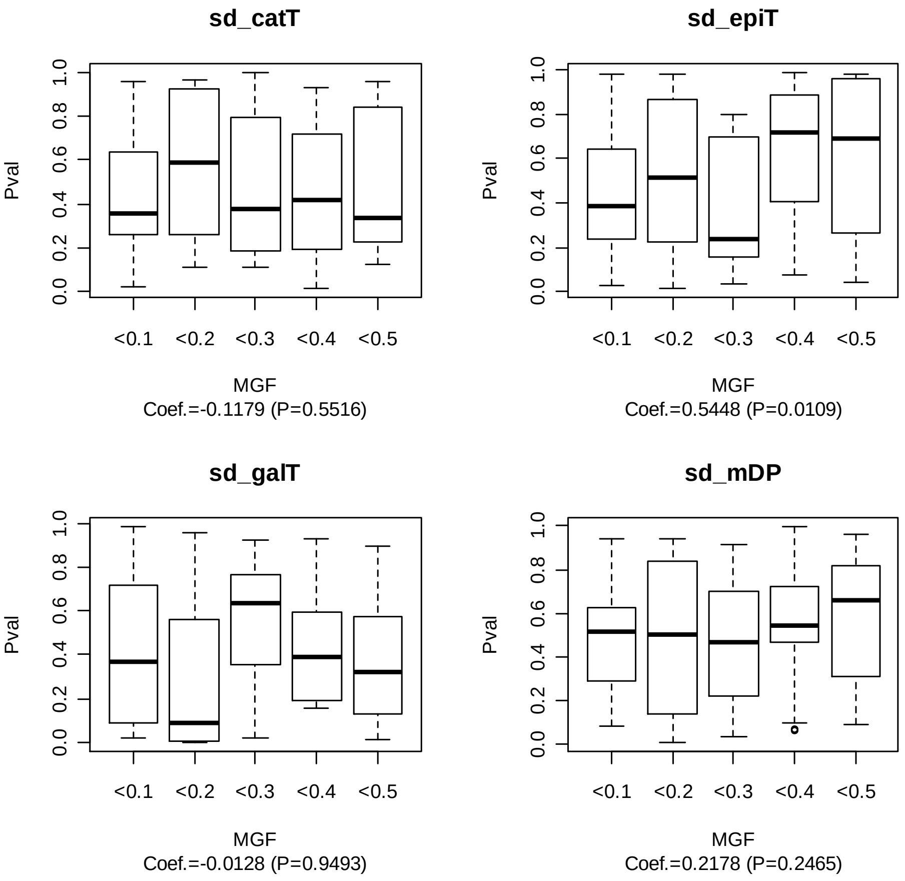
sd_catT
Pval
0.0 0.2 0.4 0.6 0.8 1.0
<0.1 <0.2 <0.3 <0.4 <0.5
MGF
Coef.=-0.1179 (P=0.5516)
sd_epiT
Pval
0.0 0.2 0.4 0.6 0.8 1.0
<0.1 <0.2 <0.3 <0.4 <0.5
MGF
Coef.=0.5448 (P=0.0109)
sd_galT
Pval
0.0 0.2 0.4 0.6 0.8 1.0
<0.1 <0.2 <0.3 <0.4 <0.5
MGF
Coef.=-0.0128 (P=0.9493)
sd_mDP
Pval
0.0 0.2 0.4 0.6 0.8 1.0
<0.1 <0.2 <0.3 <0.4 <0.5
MGF
Coef.=0.2178 (P=0.2465)

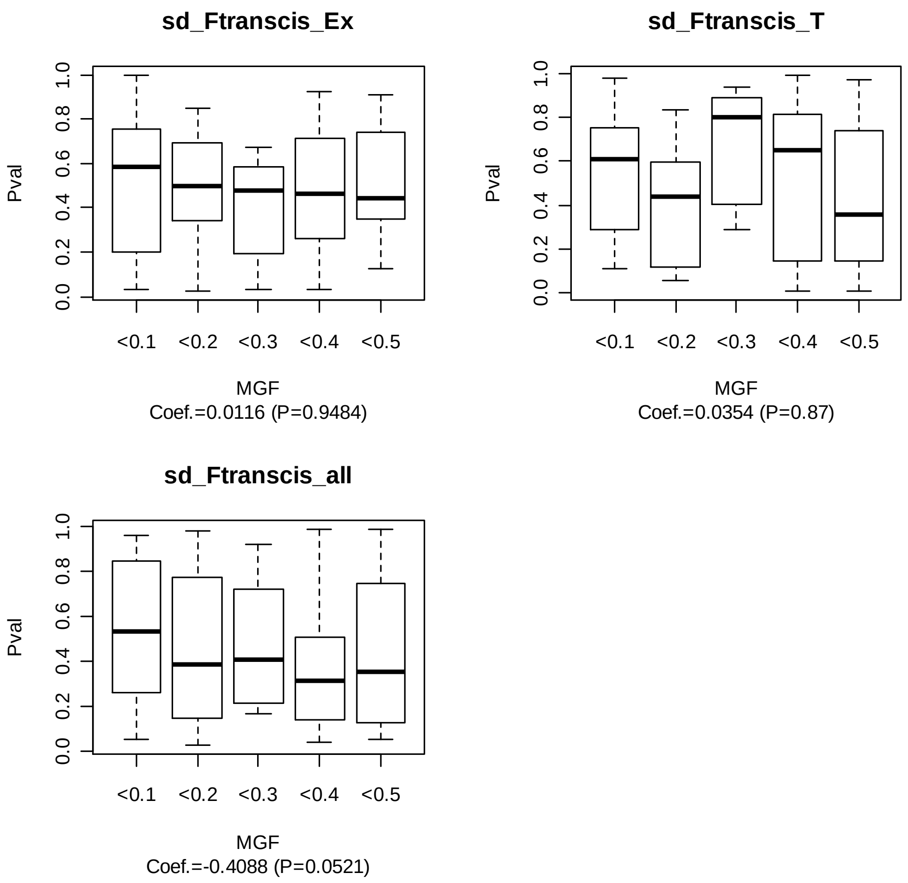

Figure S 1 : Boxplots of association test *P*-values according to minor genotypic frequency (MGF). PA variables and corresponding grape tissues (sk for skin and sd for seed) are shown at the top of each panel. Markers are partitioned into 5 bins according to minor genotypic frequency: <0.1, <0.2, <0.3, <0.4 and <0.5. y-axis indicates the *P*-value of association tests. The coefficient of regression (*P*-value in association test ~MGF) and its related significance (*P*-value) are indicated at the bottom of x-axis.

2. Test for the effect of the non-normality of the trait in the association tests

We performed association tests on BLUPs (for skin variables) and raw data (seed variables). Validity of mixed model to extract BLUPs was verified by plotting residual and BLUPs against a theoretical normal distribution (N(0, σ^2)). On the other hand, raw seed data of several variables studied in this work exhibited skewed distribution (cf. Figure 2). We used directly raw seed data in association test since the use of mixed models assumes the normal distribution of residual and linear predictors. To test the influence of trait distribution on association results, we simulated for each trait 1000 times random samplings from a normal distribution of the same mean and variance as the considered trait. In each simulation, we arranged the simulated phenotype in ascending order. Similarly, the genotypic data was also arranged in ascending order according to the observed phenotypic data. We then merged the two ordered lists and performed association tests using EMMA package (Kang *et al.* 2008). Similarity in distribution between *P*-values obtained from simulated data and *P*-value from observed trait was tested using two-tailed Kolmogorov-Smirnov D test . The *P*-value distribution of Kolmogorov-Smirnov test from 1000 simulations showed non-significant difference in association results between theoretical normal phenotype and initial phenotype, except for skin concK (Table S1, see below).

Table S 1 : 95% lower tail *P* value of 1000 Kolmogorov-Smirnov tests obtained by comparing initial PA variables to simulated normal PA variables with same mean and variance

Tissue	Trait	*P*
skin	concP	0.64
	concB	0.65
	concK	0.001
	catEx	0.54
	epiEx	0.76
	galEx	0.26
	egcEx	0.54
	catT	0.34
	epiT	0.11
	mDP	0.20
	Ftranscis_Ex	0.65
	Ftranscis_T	0.54
	Ftranscis_all	0.76
	F3pr35	0.15
seed	concP	0.26
	concB	0.26
	concK	0.11
	catEx	0.26
	epiEx	0.76
	galEx	0.34
	catT	0.65
	epiT	0.43
	galT	0.20
	mDP	0.54
	Ftranscis_Ex	0.11
	Ftranscis_T	0.11
	Ftranscis_all	0.26

Difference in *P*-value magnitude between simulated data and observed traits was tested using ANOVA (Table S 2). No significant difference in *P*-value was observed between simulated and observed traits.

Table S 2 95% higher tail *P*-value of 1000 ANOVA comparing mean *P* values obtained from initial PA variables and simulated normal PA variables

Tissue	Trait	*P*
skin	concP	0.99
	concB	0.99
	concK	0.17
	catEx	0.98
	epiEx	0.99
	galEx	0.90
	egcEx	0.97
	catT	0.99
	epiT	0.49
	mDP	0.99
	Ftranscis_T	0.99
	Ftranscis_Ex	0.98
	Ftranscis_all	0.93
	F3pr35	0.70
seed	concP	0.98
	concB	0.91
	concK	0.40
	catEx	0.98
	epiEx	0.99
	galEx	0.95
	catT	0.99
	epiT	0.93
	galT	0.87
	mDP	0.99
	Ftranscis_T	0.53
	Ftranscis_Ex	0.94
	Ftranscis_all	0.98

The test for the effect of phenotype non-normality shows that there is no significant difference between results obtained from observed traits and simulated normal traits. Meanwhile, caution should be taken for the interpretation of skin concK results. Although we worked on BLUPs for skin concK, the Kolmogorov-Smirnov test showed significant difference (P=0.001) in P value distribution between observed concK and simulated concK. P-values of observed data tended to be larger than P-values of simulated data (P_{obs}~P_{simul}, coefficient of regression=0.082). This means that we may have lost significant association due to loss of power for this trait.

Reference

Kang, H.M., Zaitlen, N.A., Wade, C.M., Kirby, A., Heckerman, D., Daly, M.J. and Eskin, E. (2008) Efficient Control of Population Structure in Model Organism Association Mapping. *Genetics*, **178**, 1709-1723.
