# Supplementary material for: Dissecting genetic architecture of grape proanthocyanidin composition through quantitative trait locus mapping
Source: BMC Plant Biol. 2012 Feb 27;12:30. doi: 10.1186/1471-2229-12-30 (PMC3312867; doi:10.1186/1471-2229-12-30)
Supplement: Additional file 5 — Summary of PA variable distributions and broad sense heritability (H2) in S × G and CC populations. Two tables inside: Table A, summary of S × G population; Table B, summary of CC population. Skin data were collected in 2005 and 2006 and seed data were collected in 2006 and 2007. Parental values are indicated as mean ± standard error. Broad sense heritability (H2) was estimated based on the best fitted model as the percentage of phenotypic variance explained by the genotypic variance. [file 1471-2229-12-30-S5.PDF]

## Additional file 5: Summary of PA variable distributions and broad sense heritability ($H^2$) in S×G and CC populations.

**Table A: Summary of S×G population**

Skin data were collected in 2005 and 2006 and seed data were collected in 2006 and 2007. Parental values are indicated as mean ± standard error. Broad sense heritability ($H^2$) was estimated based on the best fitted model as the percentage of phenotypic variance explained by the genotypic variance. NA, not available.

| | | Skin | | | | | | Seed | | | | | |
|---|---|---|---|---|---|---|---|---|---|---|---|---|---|
| | | Parents | | SxG | | | | Parent | | SxG | | | |
| Trait | year | Syrah | Grenache | Mean | Min | Max | $H^2$ | Syrah | Grenache | Mean | Min | Max | $H^2$ |
| concP | 2005 | 21.58±1.67 | 12.91±1.13 | 17.08 | 3.14 | 37.92 | | | | | | | |
| | 2006 | 21.22 ±1.19 | 13.43±0.65 | 16.78 | 5.90 | 34.65 | 0.46 | 93.45±3.47 | 90.18±2.06 | 86.08 | 39.14 | 133.49 | 0.42 |
| | 2007 | | | | | | | 76.84±4.75 | 71.38±1.35 | 82.72 | 37.99 | 136.63 | |
| concB | 2005 | 4.30±0.35 | 4.30±0.41 | 4.65 | 0.46 | 11.57 | | | | | | | |
| | 2006 | 3.67±0.23 | 3.48±0.24 | 3.92 | 0.87 | 10.13 | 0.43 | 5.74±0.54 | 5.21±0.31 | 6.14 | 1.72 | 14.98 | 0.26 |
| | 2007 | | | | | | | 4.21±0.42 | 3.74±0.09 | 5.63 | 2.04 | 14.23 | |
| concK | 2005 | 2402±197 | 1974±163 | 2540 | 280 | 6577 | | | | | | | |
| | 2006 | 2296±189 | 1695±146 | 2252 | 651 | 5822 | 0.48 | 3839±342 | 2798±227 | 3580.00 | 940 | 9597 | 0.50 |
| | 2007 | | | | | | | 1867±190 | 1607±44 | 2706.00 | 992 | 6735 | |
| catEx | 2005 | 1.34±0.07 | 2.20±0.19 | 2.35 | 0.85 | 4.68 | | | | | | | |
| | 2006 | 1.19±0.09 | 1.95±0.06 | 1.52 | 0.61 | 3.23 | 0.39 | 6.62±0.19 | 7.68±0.11 | 7.07 | 3.82 | 12.43 | 0.44 |
| | 2007 | | | | | | | 7.10±0.41 | 6.82±0.10 | 6.17 | 3.74 | 11.15 | |
| epiEx | 2005 | 51.27±1.07 | 60.75±1.13 | 55.22 | 35.94 | 74.68 | | | | | | | |
| | 2006 | 50.21±0.70 | 57.03±0.89 | 49.34 | 14.35 | 66.68 | 0.71 | 52.88±0.63 | 50.64±0.21 | 51.61 | 42.47 | 59.97 | 0.53 |
| | 2007 | | | | | | | 50.04±1.06 | 50.06±0.23 | 50.58 | 41.10 | 59.48 | |
| galEx | 2005 | 4.22±0.27 | 4.43±0.22 | 4.74 | 2.30 | 9.28 | | | | | | | |
| | 2006 | 5.24±0.17 | 6.48±0.32 | 5.86 | 2.72 | 10.68 | 0.72 | 20.65±0.37 | 22.37±0.29 | 21.40 | 10.00 | 28.45 | 0.42 |
| | 2007 | | | | | | | 23.84±1.02 | 24.83±0.24 | 23.12 | 10.48 | 31.86 | |
| egcEx | 2005 | 39.76±1.25 | 28.06±1.30 | 34.66 | 14.95 | 57.48 | | | | | | | |
| | 2006 | 40.86±0.78 | 32.84±1.14 | 40.63 | 21.78 | 67.90 | 0.73 | | | | | | |
| | 2007 | | | | | | | | | | | | |
| catT | 2005 | 2.95±0.16 | 4.26±0.16 | 2.70 | 0.78 | 6.07 | | | | | | | |
| | 2006 | 2.26±0.08 | 3.43±0.13 | 2.42 | 0.77 | 6.25 | 0.76 | 8.73±0.22 | 9.45±0.15 | 9.48 | 3.67 | 18.80 | 0.52 |
| | 2007 | | | | | | | 7.01±0.18 | 6.50±0.11 | 7.60 | 3.35 | 14.62 | |
| epiT | 2005 | 0.46±0.04 | 0.31±0.04 | 0.32 | 0.05 | 1.04 | | | | | | | |
| | 2006 | 0.25±0.02 | 0.28±0.03 | 0.23 | 0.02 | 0.58 | 0.35 | 5.58±0.50 | 5.29±0.17 | 5.74 | 1.84 | 12.69 | 0.54 |
| | 2007 | | | | | | | 6.61±0.41 | 6.28±0.17 | 6.84 | 1.87 | 16.02 | |
| galT | 2005 | | | | | | | | | | | | |
| | 2006 | | | | | | | 5.53±0.24 | 4.57±0.07 | 4.70 | 2.83 | 6.90 | 0.48 |
| | 2007 | | | | | | | 5.40±0.32 | 5.52±0.09 | 5.69 | 3.72 | 8.08 | |
| mDP | 2005 | 28.89±1.68 | 21.38±0.71 | 39.67 | 14.49 | 105.62 | | | | | | | |
| | 2006 | 38.86±1.42 | 26.52±1.04 | 42.41 | 13.93 | 108.55 | 0.82 | 5.19±0.21 | 5.18±0.07 | 5.22 | 3.07 | 9.77 | 0.50 |
| | 2007 | | | | | | | 5.28±0.17 | 5.56±0.09 | 5.20 | 3.08 | 10.15 | |
| Ftranscis_Ex | 2005 | 0.01±0.00 | 0.02±0.00 | 0.02 | 0.01 | 0.05 | | | | | | | |
| | 2006 | 0.01±0.00 | 0.02±0.00 | 0.02 | 0.01 | 0.03 | 0.38 | 0.09±0.00 | 0.11±0.00 | 0.10 | 0.05 | 0.19 | 0.39 |
| | 2007 | | | | | | | 0.10±0.01 | 0.09±0.00 | 0.08 | 0.05 | 0.16 | |
| Ftranscis_T | 2005 | 6.53±0.33 | 15.35±2.45 | 9.59 | 1.96 | 33.42 | | | | | | | |
| | 2006 | 9.68±0.72 | 13.46±0.82 | 11.86 | 2.26 | 44.45 | 0.25 | 0.81±0.04 | 0.97±0.02 | 0.93 | 0.41 | 1.82 | 0.32 |
| | 2007 | | | | | | | 0.60±0.04 | 0.56±0.01 | 0.62 | 0.31 | 1.58 | |
| Ftranscis_all | 2005 | 0.04±0.00 | 0.07±0.00 | 0.05 | 0.02 | 0.10 | | | | | | | |
| | 2006 | 0.04±0.00 | 0.06±0.00 | 0.04 | 0.02 | 0.09 | 0.65 | 0.18±0.00 | 0.21±0.00 | 0.20 | 0.10 | 0.35 | 0.43 |
| | 2007 | | | | | | | 0.16±0.01 | 0.15±0.00 | 0.16 | 0.08 | 0.31 | |
| F3pr35 | 2005 | 1.53±0.07 | 2.61±0.16 | 2.13 | 0.74 | 5.67 | | | | | | | |
| | 2006 | 1.46±0.05 | 2.30±0.11 | 1.56 | 0.47 | 3.59 | 0.65 | | | | | | |
| | 2007 | | | | | | | | | | | | |

**Table B: Summary of PA composition from CC population**

Skin data were collected in 2005 and 2006 and seed data ~~were collected~~ in 2006. Broad sense heritability ($H^2$) was estimated based on the best fitted model as the percentage of phenotypic variance explained by the genotypic variance for skin data only (collected over two years).

| | | Skin | | | | Seed | | |
|---|---|---|---|---|---|---|---|---|
| Trait | year | Mean | Min | Max | $H^2$ | Mean | Min | Max |
| concP | 2005 | 24.68 | 5.87 | 49.26 | 0.34 | | | |
| | 2006 | 16.35 | 3.63 | 35.09 | | 98.0 | 70.9 | 150.4 |
| concB | 2005 | 6.01 | 1.11 | 17.07 | 0.47 | | | |
| | 2006 | 3.71 | 0.90 | 7.87 | | 8.30 | 1.52 | 18.79 |
| concK | 2005 | 2204 | 442 | 5131 | 0.41 | | | |
| | 2006 | 1411 | 420 | 3274 | | 3250 | 1230 | 7927 |
| catEx | 2005 | 2.36 | 0.76 | 5.53 | 0.48 | | | |
| | 2006 | 1.88 | 0.56 | 3.44 | | 7.63 | 2.87 | 15.49 |
| epiEx | 2005 | 54.65 | 28.50 | 78.7 | 0.73 | | | |
| | 2006 | 51.43 | 24.93 | 79.37 | | 39.9 | 27.2 | 53.3 |
| galEx | 2005 | 3.43 | 1.00 | 10.51 | 0.75 | | | |
| | 2006 | 4.50 | 0.65 | 10.86 | | 29.61 | 16.87 | 43.09 |
| egcEx | 2005 | 36.24 | 9.48 | 65.78 | 0.77 | | | |
| | 2006 | 37.97 | 7.45 | 67.75 | | | | |
| catT | 2005 | 2.99 | 0.53 | 7.46 | 0.84 | | | |
| | 2006 | 3.89 | 1.17 | 8.75 | | 11.46 | 4.71 | 22.96 |
| epiT | 2005 | 0.33 | 0.08 | 1.00 | 0.62 | | | |
| | 2006 | 0.34 | 0.05 | 1.24 | | 7.10 | 2.03 | 14.02 |
| galT | 2005 | | | | | | | |
| | 2006 | | | | | 4.31 | 0.46 | 8.62 |
| mDP | 2005 | 34.98 | 10.12 | 124.92 | 0.80 | | | |
| | 2006 | 26.53 | 10.55 | 62.58 | | 4.5 | 2.3 | 8.8 |
| Ftranscis_Ex | 2005 | 0.04 | 0.02 | 0.1 | 0.49 | | | |
| | 2006 | 0.02 | 0.01 | 0.04 | | 0.11 | 0.04 | 0.21 |
| Ftranscis_T | 2005 | 10.3 | 2.3 | 27.8 | 0.23 | | | |
| | 2006 | 13.82 | 2.9 | 45.66 | | 1.1 | 0.4 | 2.8 |
| Ftranscis_all | 2005 | 0.06 | 0.02 | 0.12 | 0.78 | | | |
| | 2006 | 0.06 | 0.02 | 0.12 | | 0.24 | 0.12 | 0.57 |
| F3pr35 | 2005 | 2.21 | 0.52 | 9.55 | 0.54 | | | |
| | 2006 | 2.17 | 0.48 | 12.43 | | | | |
