# Supplementary figures and images for: Dissecting genetic architecture of grape proanthocyanidin composition through quantitative trait locus mapping
Source: BMC Plant Biol. 2012 Feb 27;12:30. doi: 10.1186/1471-2229-12-30 (PMC3312867; doi:10.1186/1471-2229-12-30)

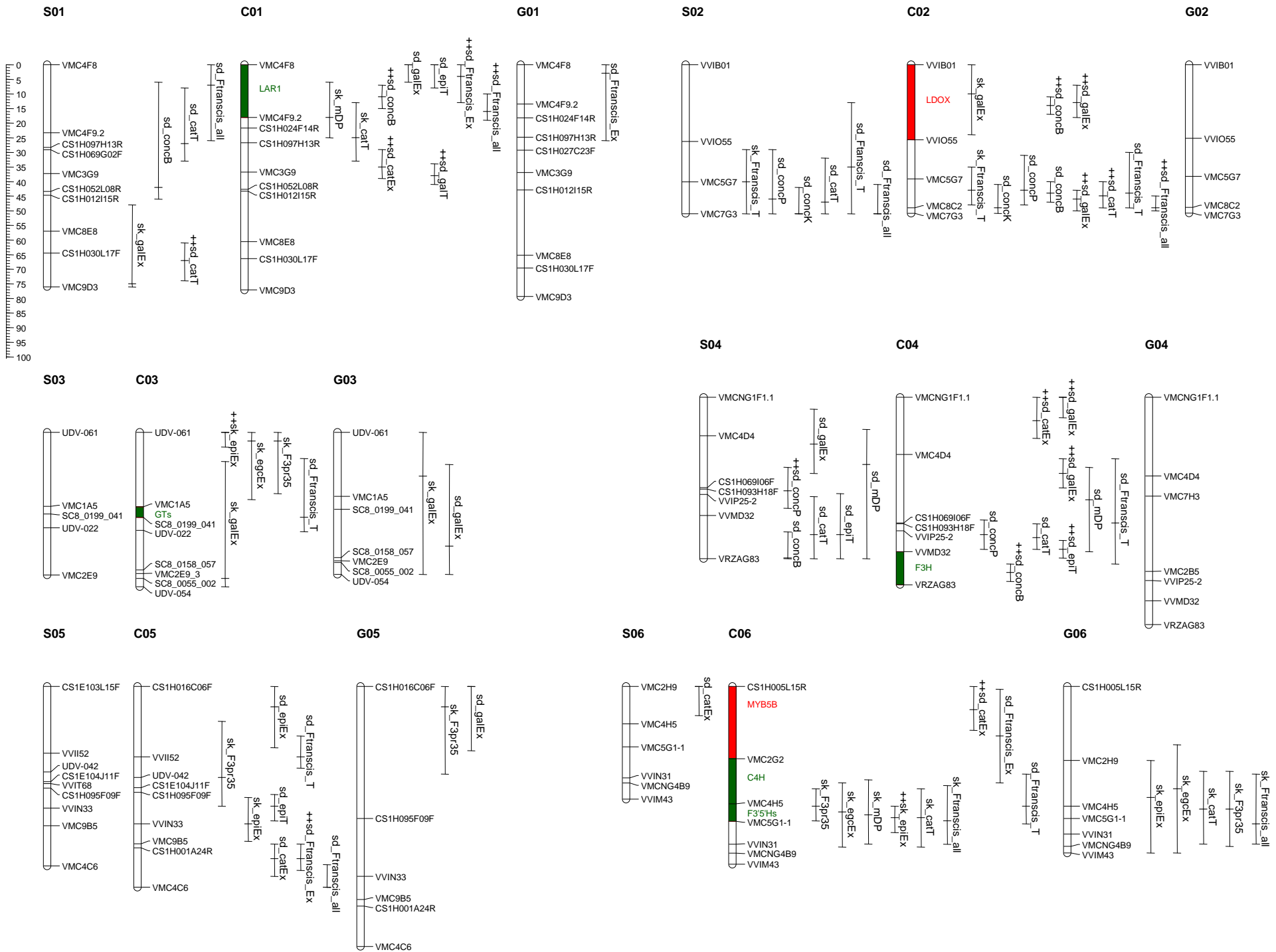

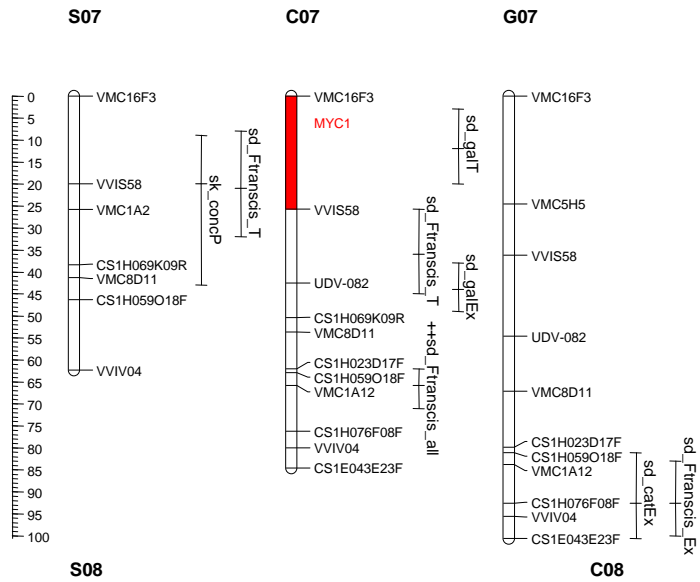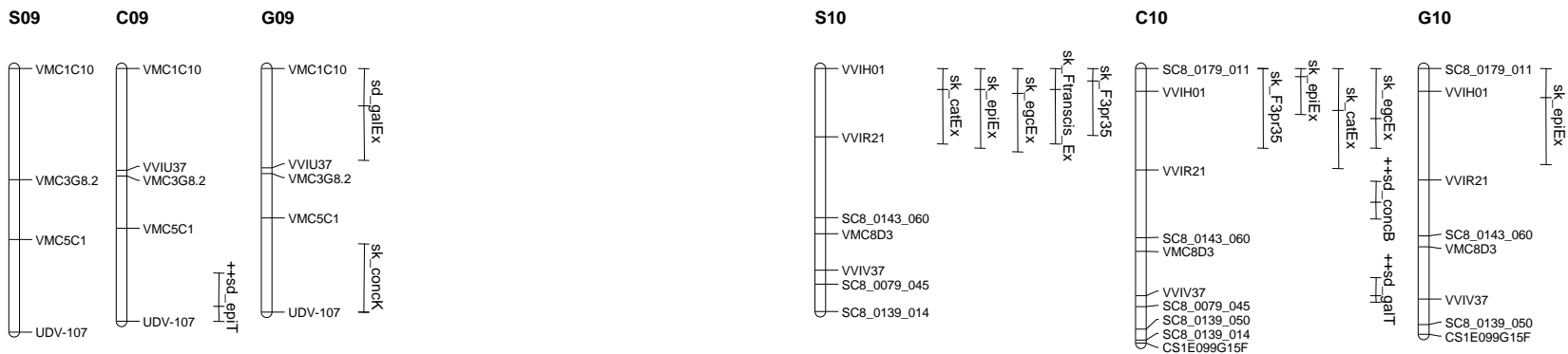

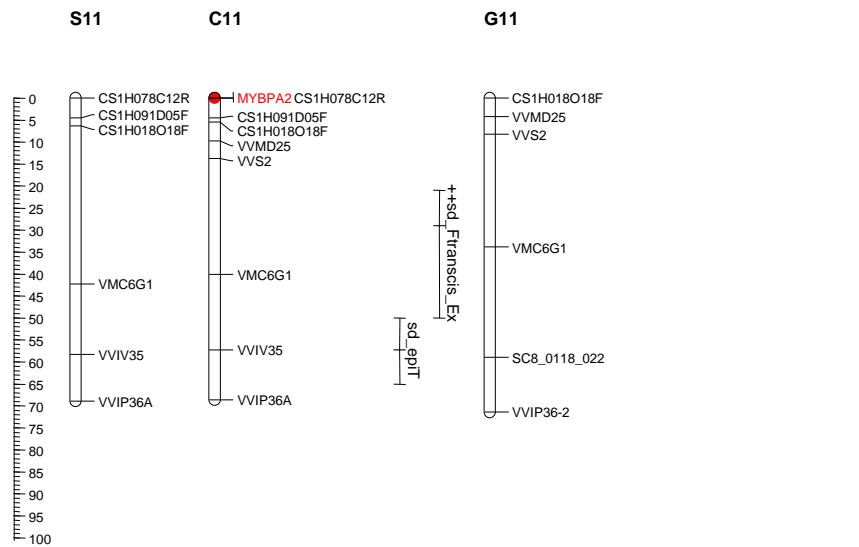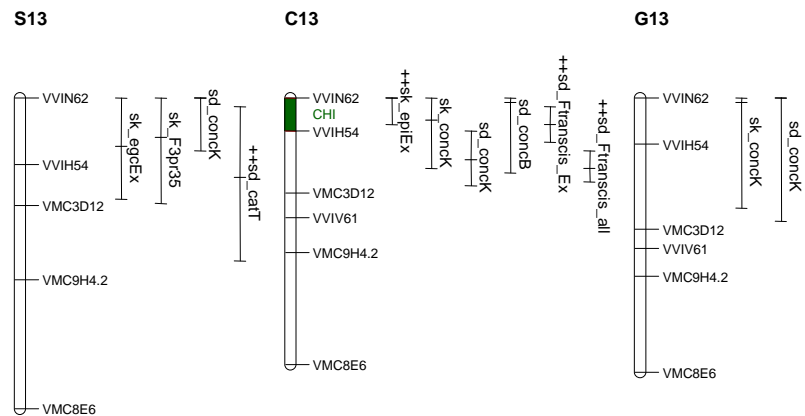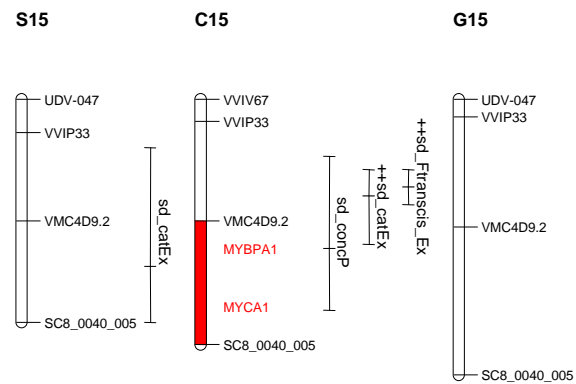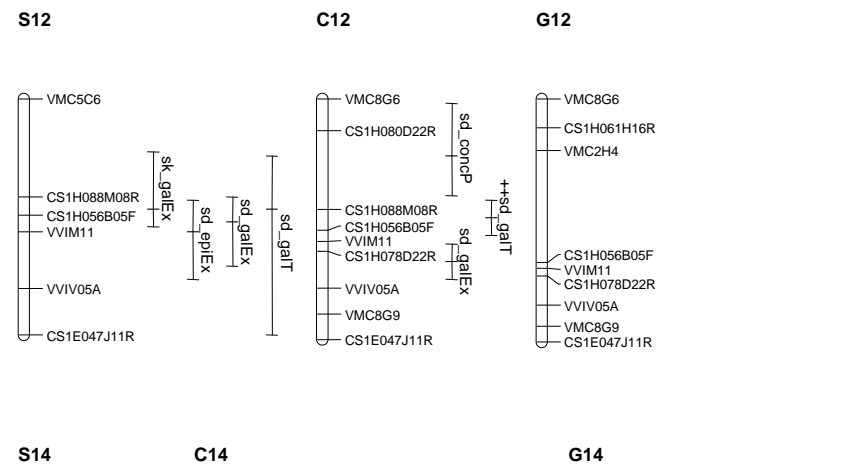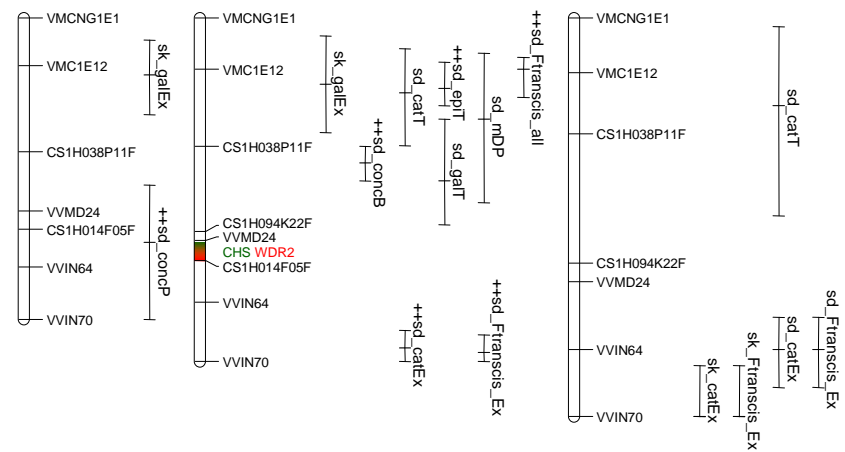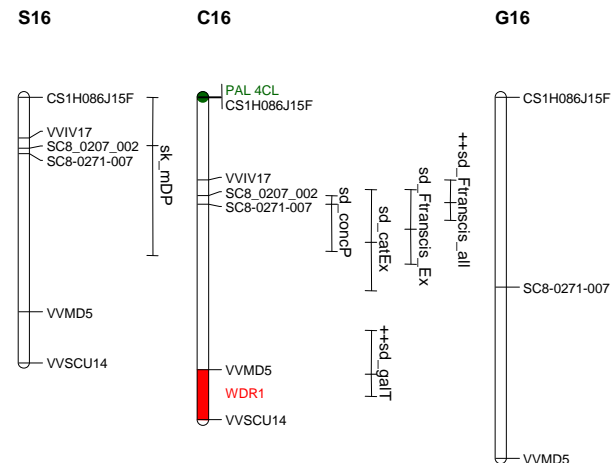

S17

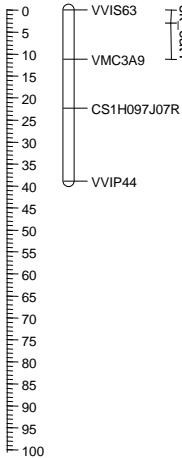

C17

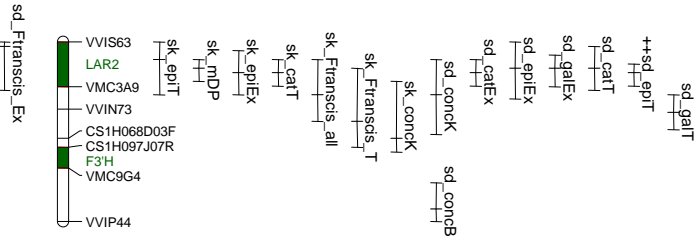

G17

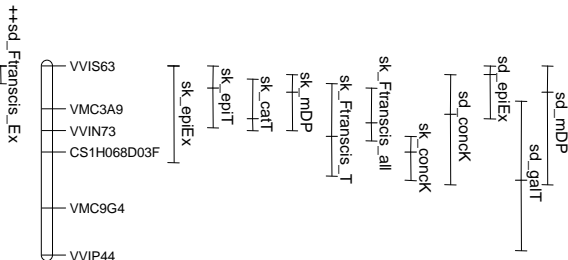

S18

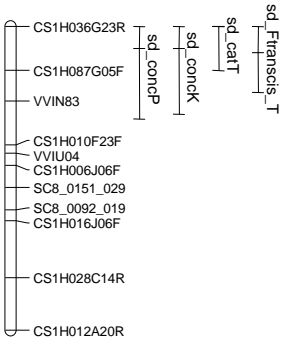

C18

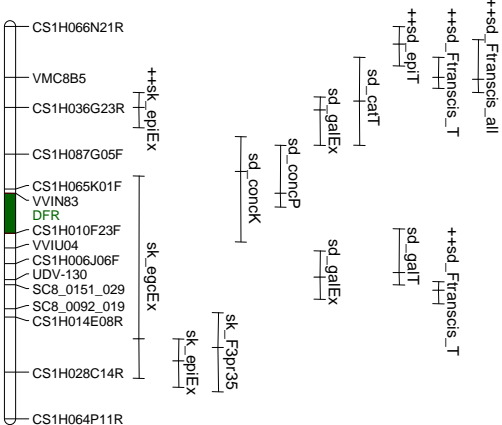

G18

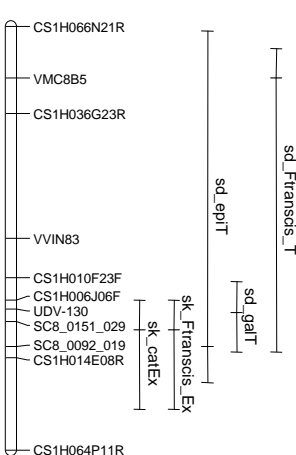

S19

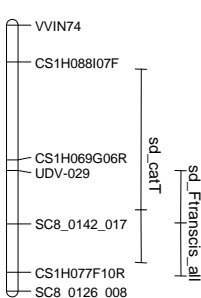

C19

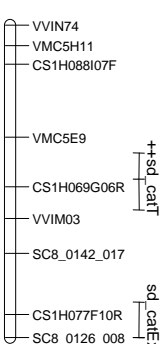

G19

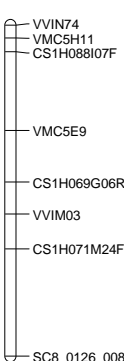

Supplement: Additional file 7 — QTL maps with positioned grape PA candidate genes. Parental and consensus maps are presented in parallel: left, Syrah map, indicated by S; centre, consensus map, indicated by C and right, Grenache map, indicated by G. QTLs are presented as vertical lines at the right side of each map: the line length corresponds to LOD-1 confidence interval and the LOD peak is indicated by a small horizontal bar in the confidence interval. Known candidate genes are positioned between flanking markers, indicated by red-filled bars, according to 12X grape genome sequence (http://www.genoscope.cns.fr). Red-filled bars indicated flanking marker interval of regulatory genes and green-filled bars for enzyme-coding genes. [file 1471-2229-12-30-S7.PDF]
