# Supplementary material for: Dissecting genetic architecture of grape proanthocyanidin composition through quantitative trait locus mapping
Source: BMC Plant Biol. 2012 Feb 27;12:30. doi: 10.1186/1471-2229-12-30 (PMC3312867; doi:10.1186/1471-2229-12-30)
Supplement: Additional file 8 — Model comparison prior to association analyses. -2lnlikelihood is shown. Model comparison was performed by likelihood ratio comparing each model to the most complete model. Significance was assessed using the χ2 distribution with degree of freedom as the difference in the number of parameter between two models. Significance level is indicated as *, P < 0.05, **, P < 0.01, ***, P < 0.001. [file 1471-2229-12-30-S8.PDF]

Additional file 8: Model comparison prior to association tests

Total content	skin			seed		
	concP	concB	concK	concP	concB	concK
Simple	701.4 **	440.4 **	1968.0 ***	913.0 **	548.2 **	1791.0 ***
K	699.8 **	438.4 *	1964.0 ***	913.0 **	547.6 *	1786.7 ***
Q	692.5	433.5	1945.1	904.3	541.4	1760.0
Q+K	692.3	432.6	1943.6	904.3	541.4	1758.3

Subunit	skin						seed					
percentage	catEx	epiEx	galEx	egcEx	catT	epiT	catEx	epiEx	galEx	catT	epiT	galT
Simple	192.4 ***	956.6 ***	392.1 **	981.9 ***	435.3 ***	-380.4	468.3 *	611.9 ***	651.5 ***	575.7 **	514.7 *	329.9
K	189.1 **	953.3 ***	389.3 *	976.8 ***	430.2 **	-380.4	468.3 *	611.3 ***	651.2 ***	575.7 **	514.6 *	329.9
Q	182.2	940.1	385.0	961.1	423.2	-379.3	463.5	597.2	638.3	568.3	508.7	327.5
Q+K	179.8	938.4	384.2	958.7	421.1	-379.3	463.5	597.1	638.3	568.3	508.7	327.5

Composite	skin					seed			
ratio	mDP	F3pr35	Ftranscis_Ex	Ftranscis_T	Ftranscis_all	mDP	Ftranscis_Ex	Ftranscis_T	Ftranscis_all
Simple	1030.0 ***	378.0 **	-1012.0	544.5 **	-683.2 ***	353.6 *	-393.6	93.2 *	-268.0
K	1025.5 ***	374.2 **	-1013.8	544.5 **	-689.5 *	353.6 *	-393.6	93.1 *	-268.0
Q	1011.0	370.3	-1007,3	536.7	-691.4	349.6	-389.7	88.6	-265.4
Q+K	1010.1	367.5	-1008.9	536.7	-694.4	349.6	-389.7	88.6	-265.4

-2lnlikelihood is shown. Model comparison was performed by likelihood ratio comparing each model to the most complete model.

Significance was assessed using the χ^2 distribution with degree of freedom as the difference in the number of parameter between two models.

Significance level is indicated as *, *P*<0.05, **, *P*<0.01, ***, *P*<0.001.
